# Naturally Occurring Chromone Glycosides: Sources, Bioactivities, and Spectroscopic Features

**DOI:** 10.3390/molecules26247646

**Published:** 2021-12-16

**Authors:** Yhiya Amen, Marwa Elsbaey, Ahmed Othman, Mahmoud Sallam, Kuniyoshi Shimizu

**Affiliations:** 1 Department of Agro-Environmental Sciences, Graduate School of Bioresources and Bioenvironmental Sciences, Kyushu University, Fukuoka 819-0395, Japan; yhiaamen@mans.edu.eg (Y.A.); ah.othman@azhar.edu.eg (A.O.); 2 Department of Pharmacognosy, Faculty of Pharmacy, Mansoura University, Mansoura 35516, Egypt; marwaelsebaey1611@mans.edu.eg; 3 Department of Pharmacognosy, Faculty of Pharmacy, Al-Azhar University, Cairo 11371, Egypt; m.sallam@azhar.edu.eg

**Keywords:** chromone glycosides, chemical structure, activity, benzo-γ-pyrone, ^13^C-NMR data

## Abstract

Chromone glycosides comprise an important group of secondary metabolites. They are widely distributed in plants and, to a lesser extent, in fungi and bacteria. Significant biological activities, including antiviral, anti-inflammatory, antitumor, antimicrobial, etc., have been discovered for chromone glycosides, suggesting their potential as drug leads. This review compiles 192 naturally occurring chromone glycosides along with their sources, classification, biological activities, and spectroscopic features. Detailed biosynthetic pathways and chemotaxonomic studies are also described. Extensive spectroscopic features for this class of compounds have been thoroughly discussed, and detailed ^13^C-NMR data of compounds **1**–**192**, have been added, except for those that have no reported ^13^C-NMR data.

## 1. Introduction

Chromone glycosides are a class of secondary metabolites with various medicinal properties. They are widely distributed in many plant genera and, to a lesser extent, in some fungal species and other sources [1]. Several biological activities have been reported for various chromone glycosides. For example, aloesin and its analogues, from Aloe, are used in cosmetic preparations to treat hyperpigmentation induced by UV radiation, owing to their role in inhibition of tyrosinase enzyme [2,3]. Additionally, 8-[C-*β*-d-[2-*O*-(*E*)-cinnamoyl]glucopyranosyl]-2-[(*R*)-2-hydroxypropyl]-7-methoxy-5-methylchromone), isolated from certain Aloe species, was reported to have potent topical anti-inflammatory activity comparable to the effect of hydrocortisone without affecting thymus weight [3]. Macrolobin, from *Macrolobium latifolium*, has a remarkable acetylcholinesterase inhibitory activity with an IC_50_ value of 0.8 µM. Uncinosides A and B, isolated from the Chinese herbal medicine *Selaginella uncinata*, showed potent anti-RSV (respiratory syncytial virus) activity with IC_50_ values of 6.9 and 1.3 µg/mL. Taking into consideration the broad biological activities of chromone glycosides, this review summarizes the naturally occurring chromone glycosides and categorizes these compounds on their structural basis, in addition to their sources, bioactivities and spectroscopic features. Importantly, this review will shed more light toward the NMR features of chromone glycosides to help natural product researchers in the identification of various chemical structures. Scientific databases as SciFinder, PubMed, and Google Scholar were used to collect the relevant literature data.

## 2. Biosynthesis

**Scheme 1:**
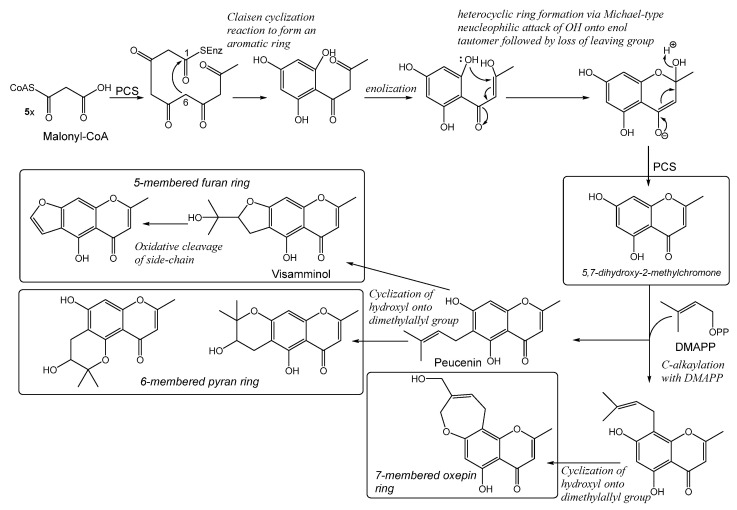
Proposed mechanisms for the enzymatic formation of 5,7-dihydroxy-2-methylchromone and its derivatives.

Chromones are biosynthesized through the acetic acid pathway by the condensation of five acetate molecules. These compounds, generally, have a methyl group at C-2 and are oxygenated at C-5 and C-7 [4]. Pentaketide Chromone Synthase (PCS) is a key enzyme in the biosynthesis process that catalyzes the formation of a pentaketide chromone (5,7-dihydroxy-2-methylchrome) from five-step decarboxylative condensations of malonyl-CoA, followed by the Claisen cyclization reaction to form an aromatic ring. However, it is unclear whether the heterocyclic ring closure of the pentaketide chromone is enzymatic or not, because the ring closure can take place due to spontaneous Michael-like ring closure, as in the case of flavanone formation from chalcone in vitro. PCS also accepts acetyl-CoA, resulting from decarboxylation of malonyl-CoA, as a starter substrate, but it is a poor substrate for PCS [5].The pentaketide chromone has been isolated from several plants and is known to be the biosynthetic precursor of the chromone derivatives with additional heterocyclic rings (e.g., furano-, pyrano- and oxepino-chromone glycosides). Scheme 1 ([6] with modifications) shows the sequence of steps utilized in the biosynthesis of these compounds, fully consistent with the biosynthetic rationale developed above. The key intermediate is 5,7-dihydroxy-2-methylchromone [5,6]. For many years, the cyclization had been postulated to involve an intermediate epoxide, such that nucleophilic attack of the phenol onto the epoxide group might lead to formation of either five-membered furan, six-membered pyran or the seven-membered oxepin heterocycles, as commonly encountered in natural products [6].

**Scheme 2:**
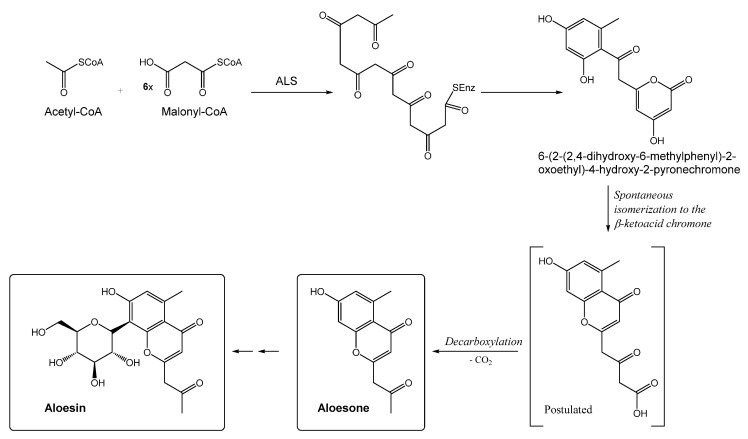
Proposed mechanisms for the enzymatic formation of aloesone and its derivatives.

Aloesone Synthase (ALS) (Scheme 2, [5] with modifications) is a key enzyme in the biosynthesis of heptaketide chromone aloesone derivatives, such as aloesone 7-*O*-β-d-glucopyranoside (**53**) in rhubarb and anti-inflammatory aloesone 8-C-β-d-glucopyranoside (aloesin, **98**) in Aloe (*A. arborescens*). ALS efficiently catalyzes the formation of a heptaketide aromatic pyrone 6-(2-(2,4-dihydroxy-6-methylphenyl)-2-oxoethyl)-4-hydroxy-2-pyronechromone from acetyl-CoA and six molecules of malonyl-CoA through an aldol cyclization. The unstable heptaketide pyrone (or acid form) would then undergo subsequent spontaneous isomerization to the β-ketoacid chromone, which is followed by decarboxylation to produce the heptaketide aloesone [5].

## 3. Taxonomy

We have reviewed the literature concerning the occurrence of chromone glycosides, and we have found that 192 different chromone glycosides have been isolated from different natural sources, including angiosperms, ferns, lichens, fungi and actinobacteria (Table 1). The occurrence of chromone glycosides is mostly confined to botanical families: Apiaceae, Fabaceae, Myrtaceae, Asphodelaceae, Ranunculaceae, Rubiaceae, Hypericaceae, Ericaceae, Amaryllidaceae, Polygonaceae and Araceae. However, few chromone glycosides are also present in Asteraceae, Eucryphiaceae, Saxifragaceae, Smilacaceae, Pentaphylaceae, Salicaceae, Meliaceae, Euphorbiaceae, Staphyleaceae, Amaranthaceae, Aquifoliaceae, Rosaceae, Bignoniaceae, Olacaceae, Pinaceae, Selaginellaceae, Gentianaceae, Cannabaceae, Euphorbiaceae, Cucurbitaceae, Thymelaeaceae and Poaceae. Many of the naturally occurring chromone-8-C-glycosides such as the well-known chromone glycoside aloesin (**98**) were reported from genus *Aloe*. Until now, chromone glycosides with additional heterocyclic moieties such as pyrano-, oxepino- and pyrido-chromone glycosides were only isolated from *Saposhnikovia divaricate*, *Eranthis* species and *Schumanniophyton magnificum*, respectively. Another interesting category comprises hybrids of furano-chromones with cycloartane triterpenes, which were reported from *Cimicifuga foetida.* Actinobacteria also constitute an important source of the chromone alkaloid aminoglycosides, which are isolated from *Streptomyces*, *Saccharothrix* and *Actinomycete* species.

## 4. Chromone Glycosides

Chromone glycosides belong to a group of oxygen-containing heterocyclic compounds with a benzo-γ-pyrone skeleton. Naturally occurring chromone glycosides can be either *O*-glycosides or C-glycosides. For *O*-glycosides, the most frequently encountered group is the 7-*O*-glycosides; however, 2-, 3-, 5-, 8-, 11- and 13-*O*-glycosides also exist but to a lower extent. For an example, only one 6-*O*-glycoside **11** has been reported from nature, and from fungi, not higher plants [1]. Glycosylation can also be detected at side chains for chromones, at C-11 and C-12 as in compounds **56**–**59**, at the hydroxyprenyl and hydroxyisoprenyl side chains as in **123** and **128**, respectively, or at the phenyl ethyl moiety as **139**. The most abundant among chromone glycosides is the glucoside from. However, other sugar moieties such as xylose, arabinose and rhamnose were also detected in 3-, 7- and 11-*O*-glycosides.

### 4.1. Chromone O-glycosides

#### 4.1.1. 2-*O*-Glycosides

**Figure 1:**
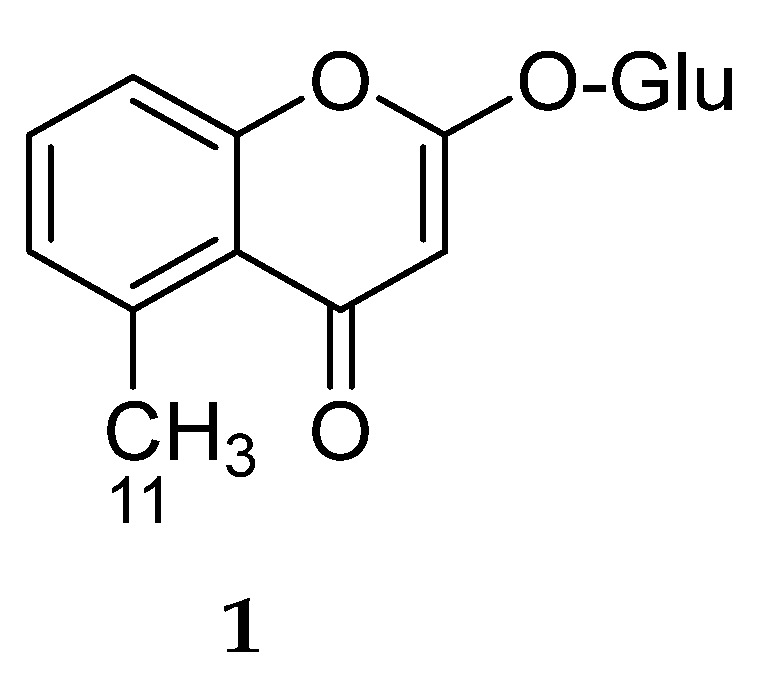
Structure of compound **1**.

This category includes compound **1** (Figure 1), 2-hydroxy-5-methylchromone-*β*-d-glucopyranoside, isolated from the aerial parts of *Mutisia acuminata* var. *hirsuta*, a member of family Asteraceae [7]. The authors did not report biological activity for this compound.

#### 4.1.2. 3-*O*-Glycosides

**Figure 2:**
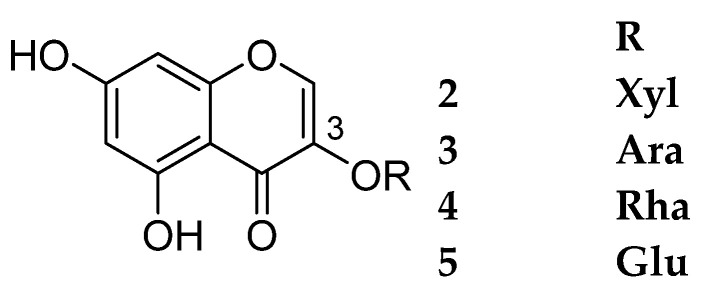
Structures of compounds **2**–**5**.

This category includes compounds **2**–**5**. They share the same aglycone nucleus but with different sugar moieties at C-3. Eucryphin **4** was reported as a new compound in 1979 [8]; however, it was reported again in 1996 as a new compound under the name smiglanin [9]. In addition, 3,5,7-trihydroxychromone 3-*O*-*β*-d-xylopyranoside **2** was first reported in 2005 from *Rhodadendron ovatum* [10], but it was reported again as a new compound in 2013 [11]. Compounds **2–5** are shown in Figure 2. The sources and the reported biological activities are summarized in Table 2.

#### 4.1.3. 5-*O*-Glycosides

**Figure 3:**
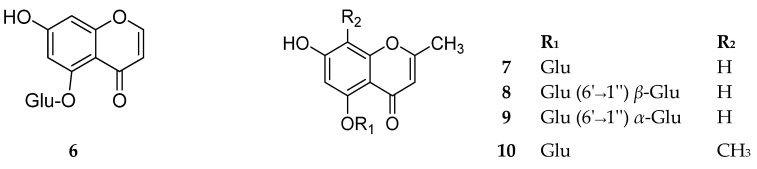
Structures of compounds **6**–**10**.

Among the naturally occurring 5-*O*-glycosides, Staphylosides A and B (**8**–**9**), isolated from *Staphylea bumalda*, are characterized by a presence of a disaccharide moiety attached to C-5. The disaccharide chain in **8** is *β*-d-glucopyranosyl-(1→6)-*β*-d-glucopyranoside while in **9**, is α-d-glucopyranosyl-(1→6)-*β*-d-glucopyranoside. Compounds **6**–**10** are shown in Figure 3. The sources and the reported biological activities (if any) are summarized in Table 3.

#### 4.1.4. 6-*O*-Glycosides

**Figure 4:**
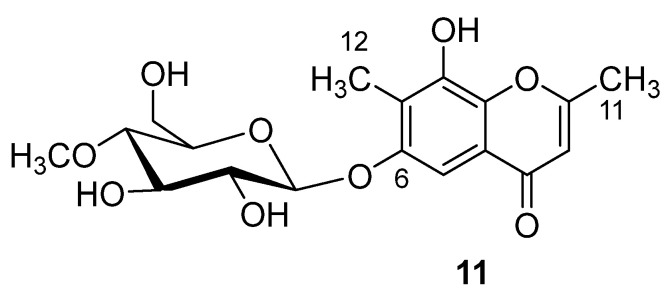
Structure of compound **11**.

Compound **11** (Figure 4) has a unique structure for bearing 4-*O*-methylglucopyranosyl unit. Chemically, it is 6-*O*-(4-*O*-methyl-*β*-d-glucopyranosyl)-8-hydroxy-2,7-dimethyl-4H-benzopyran-4-one, isolated from the rice culture of the fungus *Armillaria tabescens* [1]. Although such compounds are not common in higher plants, several of them have previously been isolated from fungi [1].

#### 4.1.5. 7-*O*-Glycosides

**Figure 5:**
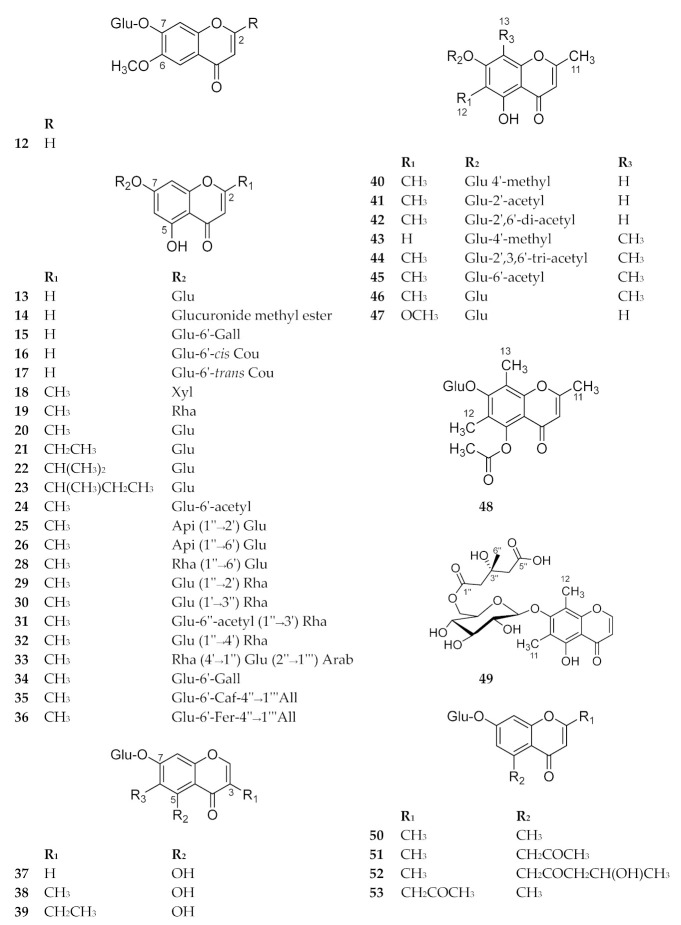
Structures of compounds **12**–**53**.

This subclass is characterized by the presence of sugar at C-7. Hyperimone A is the same as Urachromone A (**22**), reported at nearly the same time from different co-authors from the genus Hypericum. Takanechromone A (**38**) is the same as Hyperimone B, isolated from the same genus by different co-authors. They were reported each time as new compounds. We preferred to add only ^13^C-NMR data of one set of these compounds (Table 23). Several biological activities have been reported to some members of this subclass. Compounds **12**–**53** are shown in Figure 5. The sources and the reported biological activities (if any) are summarized in Table 4.

Drynachromosides C (**30**) and D (**33**) exhibited inhibitory activity on triglyceride accumulation [22]. The effects of these compounds on mRNA expression of the three adipogenesis-related marker genes, PPARγ, C/EBPα and Ap2, in 3T3-L1 were investigated. The mRNA expression levels of PPARγ, C/EBPα and Ap2 were found to be dramatically downregulated. Compounds **40** and **43**, having a unique sugar unit of 4-*O*-methyl-*β*-d-glucopyranose, were isolated from the scale-insect pathogenic fungus *Orbiocrella* sp. BCC 33248 [23]. Uncinosides A (**46**) and B (**48**) [24], isolated from the Chinese herbal medicine *Selaginella uncinata*, showed potent anti-RSV (respiratory syncytial virus) activity with IC_50_ values of 6.9 and 1.3 µg/mL, respectively. Uncinoside B (**48**) was found to have a TI value of 64.0, a large therapeutic index comparable to that of ribavirin with a TI value of 24.0, which is an approved drug for the treatment of RSV infection in humans. They also showed moderate antiviral activities against PIV 3 (parainfluenza type 3 virus) with IC_50_ values of 13.8 and 20.8 µg/mL and TI values of 6.0 and 4.0, respectively. 

#### 4.1.6. 8-*O*-Glycosides

**Figure 6:**
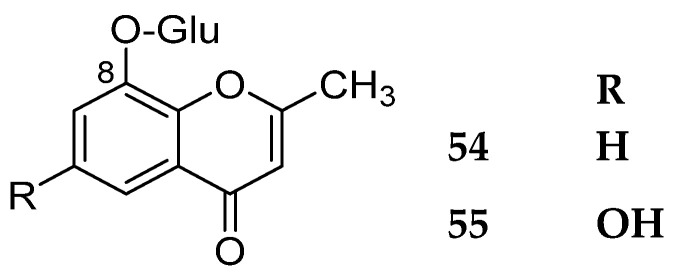
Structures of compounds **54**–**55**.

Only two compounds **54**–**55** were reported in nature. They are shown in Figure 6. The sources and the reported biological activities (if any) are summarized in Table 5.

#### 4.1.7. 11- and 13-*O*-Glycosides

**Figure 7:**
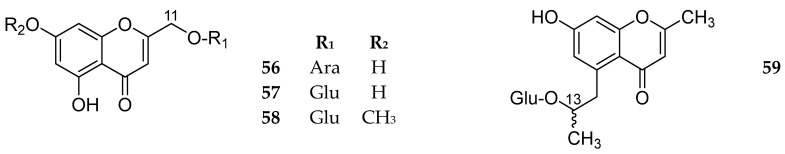
Structures of compounds **56**–**59**.

Compound **57** was reported in 2012 as Monnieriside A [60] and was then reported as Drynachromoside B [22,31,47]. Compounds **56**–**59** are shown in Figure 7. The sources and the reported biological activities (if any) are summarized in Table 6.

#### 4.1.8. Chromanone Glycosides

**Figure 8:**
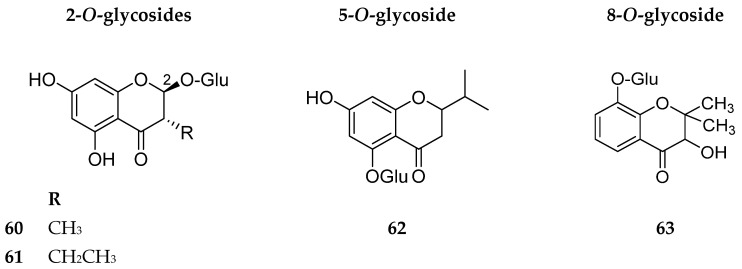
Structures of compounds **60**–**63**.

Chromanone glycosides or 2,3-dihydrochromone glycosides are not abundant in nature. Reviewing the literature, we encountered only four examples **60**, **61**, **62** and **63**. Their structures are shown in Figure 8. The sources and biological activities (if any) of these compounds are summarized in Table 7.

### 4.2. Chromone C-Glycosides

In contrast to chromone *O*-glycosides, which are widely distributed and of common occurrence, C-glycoside derivatives are rarely found out.

#### 4.2.1. 3-C-Glycosides

**Figure 9:**
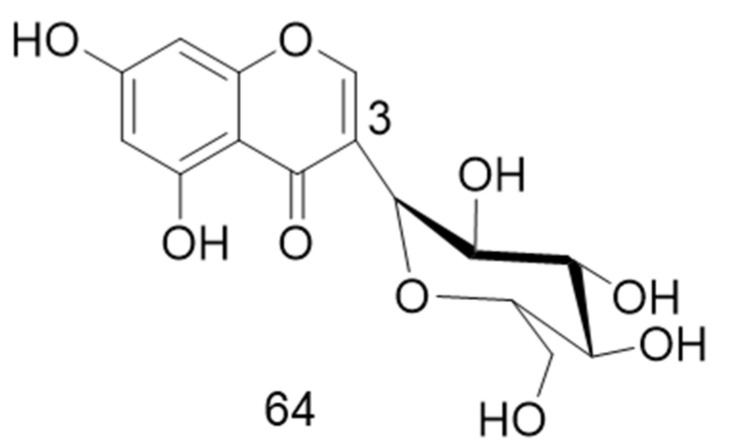
Structure of compound **64**.

This subclass includes the unusual 5,7-dihydroxychromone-3α-d-C-glucoside, named macrolobin, isolated from the aerial parts of *Macrolobium latifolium* [65]. Its structure is shown in Figure 9. Its source and biological activities are summarized in Table 8.

#### 4.2.2. 6-C-Glycosides

**Figure 10:**
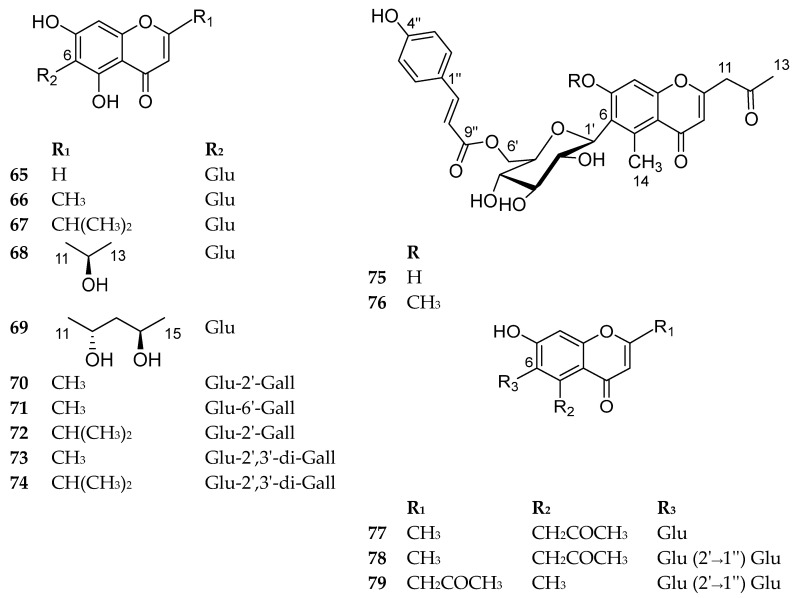
Structures of compounds **65**–**79**.

Compounds **65**–**79** are shown in Figure 10. The sources and the reported biological activities (if any) are summarized in Table 9.

#### 4.2.3. 8-C-Glycosides

**Figure 11:**
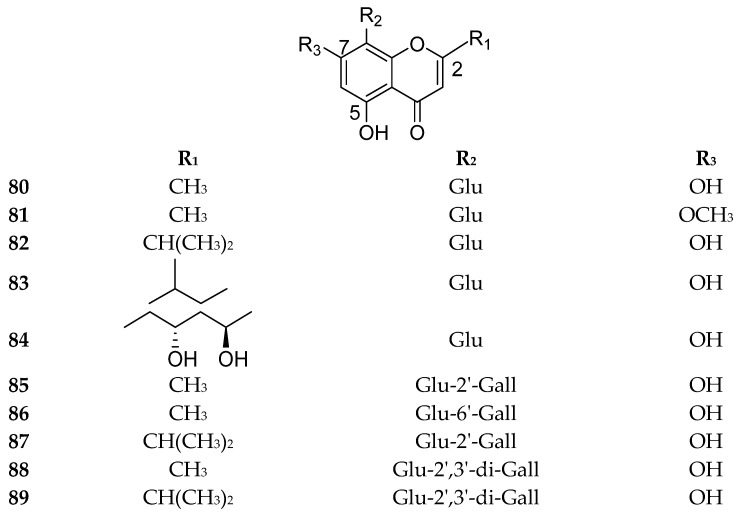
Structures of compounds **80**–**89**.

**Figure 12:**
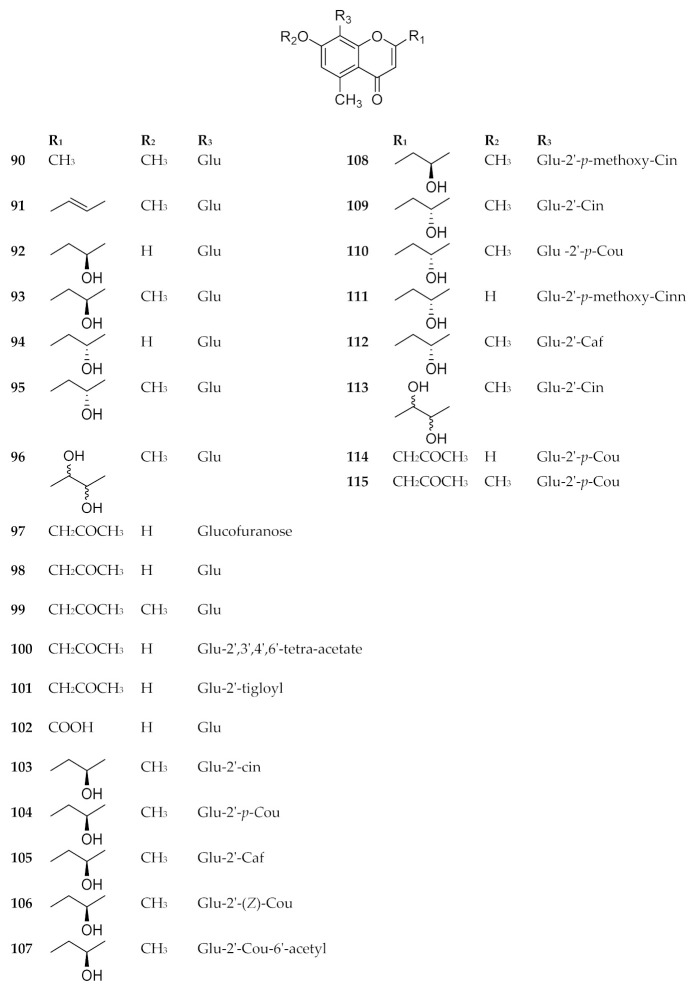
Structures of compounds **90**–**115**.

**Figure 13:**
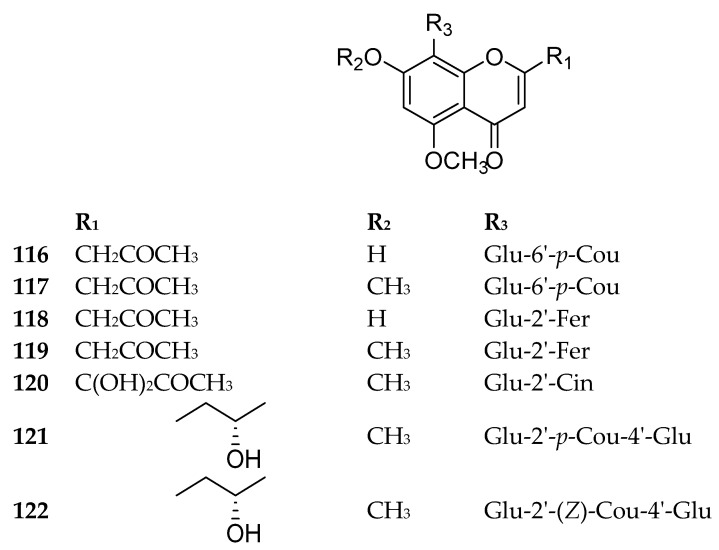
Structures of compounds **116**–**122**.

Many of the naturally occurring chromone-8-C-glycosieds can be found in genus *Aloe*. Approximately 26 chromone-8-C-glycosides were reported in the perennial plant *Aloe vera*, which is a well-known pharmaceutical herb used in traditional Chinese medicine [76]. Some significant bioactive chromone-8-C-glycosides were isolated and identified in *Aloe vera*, including Aloesin (**98**), aloeresin E (**109**), isoaloeresin D (**110**), aloeresin A (**114**) and other derivatives. For instance, aloeresin A (**114**) exhibited a promising therapeutic activity toward α-glucosidase enzyme [79], while the compound isobiflorin (**80**), isolated from the flower buds of *Syzygium aromaticum*, had the capacity to inhibit LPS-induced production of nitric oxide (NO) and prostaglandin E_2_ (PGE2) in RAW 264.7 macrophages [67]. A chromone-8-C-glycoside, 5,7-dihydroxy-2-isopropylchromone-8-*β*-d-glucoside, reported in *Hypericum japonicum*, showed an activity against Epstein–Barr virus [71]. Additionally, BACE1 (*β*-secretase), which is a possible potential target in the treatment of Alzheimer’s disease, was inhibited by some compounds as aloesin (**98**) [80], 7-*O*-methyl-aloeresin A (**115**) [81] and 2′-feruloyl-7-*O*-methylaloesin (**119**) [80]. Furthermore, tyrosinase, which is the key enzyme for controlling the production of melanin, was inhibited by aloeresin E (**109)** and isoaloeresin D (**110**) [82]. The compounds **80**–**122** are shown in Figure 11, Figure 12 and Figure 13. The sources and the reported biological activities (if any) are summarized in Table 10.

### 4.3. Phenyl and Isoprenyl Chromone Glycosides

**Figure 14:**
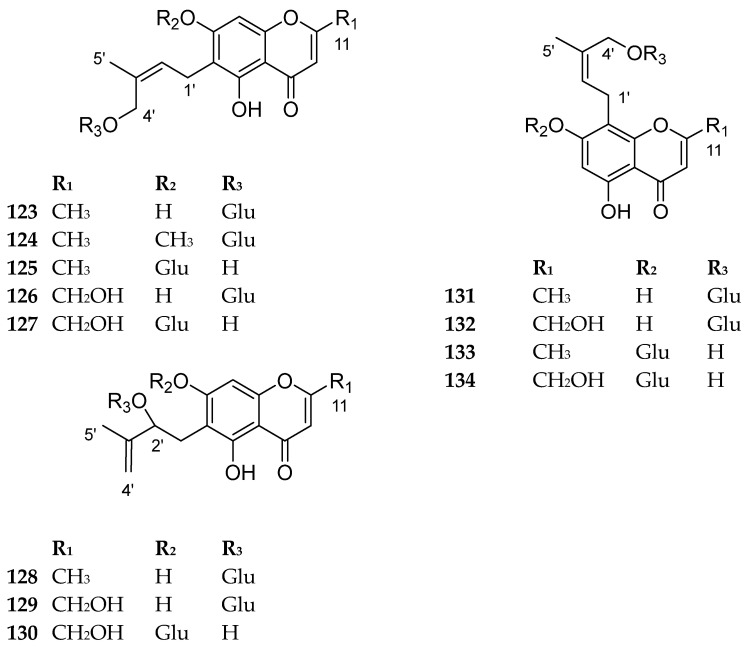
Structures of compounds **123**–**134**.

This category is characterized by a hydroxyl prenyl moiety at C-6 or C-8, or a hydroxyl isoprenyl moiety at C-6 only. The sugar moiety can be either situated at C-7 hydroxyl of the chromone nucleus or C-4’ of the hydroxyl prenyl or C-2’ of the hydroxyl isoprenyl moiety. Most of the compounds in this category were reported from the genus Cnidium, belonging to family Apiaceae. The reported biological activity associated with several compounds in this category is their significant inhibition of fat accumulation in differentiated adipocytes employing 3T3-L1 preadipocyte cells as an assay system [60]. The compounds **123**–**134** are shown in Figure 14. The sources and the reported biological activities (if any) are summarized in Table 11.

### 4.4. Phenyl Ethyl Chromone Glycosides

**Figure 15:**
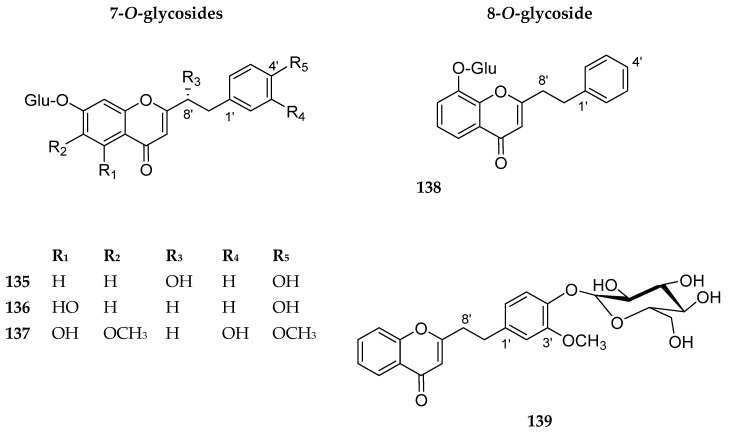
Structures of compounds **135**–**139**.

Reviewing the literature, we encountered five phenyl ethyl chromone glycosides. The phenyl ethyl moiety is usually located at C-2 of the chromone nucleus. The sugar moiety is attached to C-7 of the chromone skeleton in compounds **135**–**137**, while in compound **138**, the sugar is attached to C-8. In compound **139**, the sugar is not attached directly to the basic chromone skeleton. Compounds **135**–**139** are shown in Figure 15. Their sources are summarized in Table 12. There are no reported biological activities of these compounds.

### 4.5. Chromone Glycosides with Additional Heterocyclic Moieties

This category of chromone glycosides is further classified based on the additional heterocyclic moiety into furano-chromone glycosides, pyrano-chromone glycosides, oxepino-chromone glycosides and pyrido-chromone glycosides. 

#### 4.5.1. Furano-Chromone Glycosides

**Figure 16:**
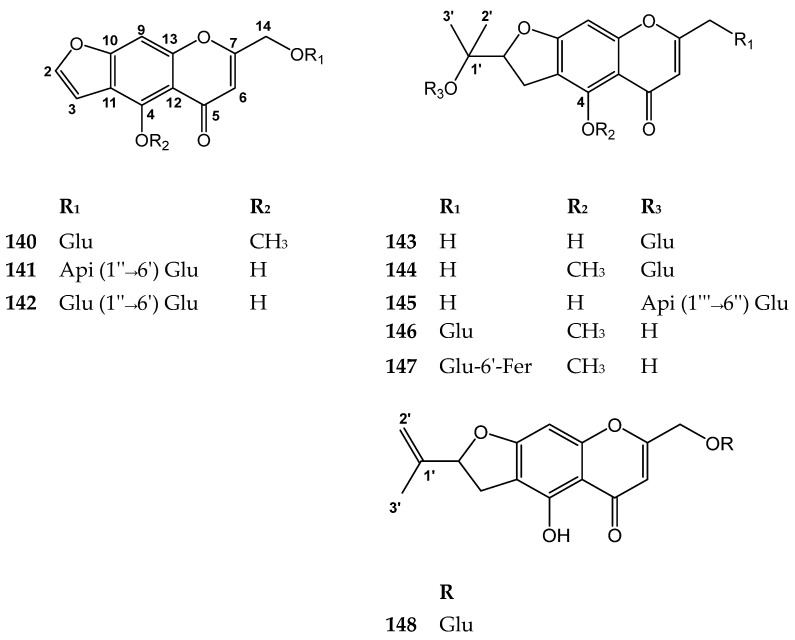
Structures of compounds **140**–**148**.

This subclass of compounds is characterized by presence of an additional furan, or a tetrahydrofuran ring fused with the benzo-δ-pyrone. Khellol glucoside (**140**), isolated from *Ammi visnaga*, is one of the important members in this subclass. It possess potent coronary vasodilator and bronchodilator activities [115]. It was reported to have a significant hypocholesterolemic effect. It lowered low-density lipoprotein cholesterol (LDL-C) by 73%, high-density lipoprotein cholesterol (HDL-C) by 23%, and total-C by 44%, after a single oral dose of 20 mg/kg per day after two weeks [116]. Compounds **140**–**148** are shown in Figure 16. The sources and the reported biological activities (if any) are summarized in Table 13.

#### 4.5.2. Pyrano-Chromone Glycosides

**Figure 17:**
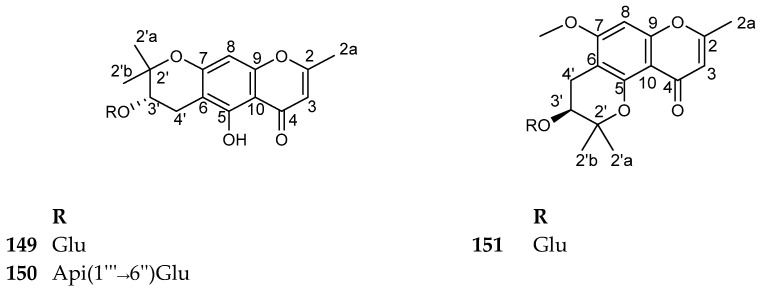
Structures of compounds **149**–**151**.

This subclass of compounds is characterized by the presence of an additional pyran ring fused with the benzo-δ-pyrone. Only three compounds were reported from nature until now. Of them, 3′-*O*-glucopyranosylhamaudol (*Sec*-*O*-glucopyranosylhamaudol) (**149**) and (3’*S*)-3′-*O*-*β*-d-apiofuranosyl-(1→6)-*β*-d-glucopyranosylhamaudol (**150**), isolated from the *Saposhnikovia divaricata*, showed weak anti-cancer activity. Both compounds were screened against three cancer cell lines, namely human prostatic cancer cell (PC-3), human ovarian carcinoma cell (SK-OV-3), and human lung cancer cell (H460) using the conventional MTT assay. Compound **149** showed a weak activity against H460, with an IC_50_ value of 94.25 ± 1.45 µM while compound **150**, showed an activity against SK-OV-3 with an IC_50_ value of 86.21 ± 1.03 µM [119]. Compounds **149**–**151** are shown in Figure 17. The sources and the reported biological activities (if any) are summarized in Table 14.

#### 4.5.3. Oxepino-Chromone Glycosides

**Figure 18:**
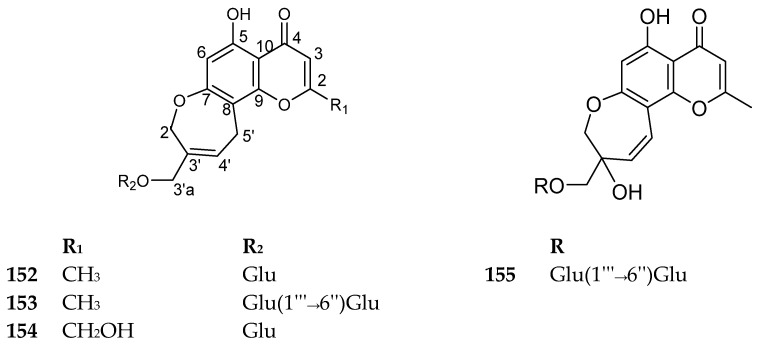
Structures of compounds **152**–**155**.

This subclass of compounds is characterized by the presence of an additional oxepin fused with the benzo-δ-pyrone. Only four compounds were reported from nature until now, and all of them were reported from *Eranthis* species. The compounds **152**–**155** are shown in Figure 18. The sources are summarized in Table 15. There are no reported biological activities for these compounds.

#### 4.5.4. Pyrido-Chromone Glycosides

**Figure 19:**
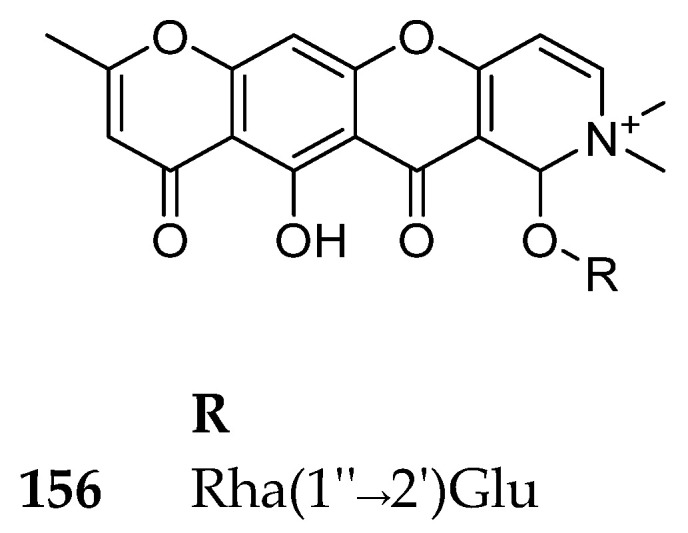
Structure of compound **156**.

This subclass includes only the chromone alkaloidal glycoside; Schumanniofoside. This compound was found to reduce the lethal effect of black cobra (*Naja melanoleuca*) venom in mice [135]. The authors proved that this effect is greatest when the venom is mixed and incubated with the extract or schumanniofoside. They concluded that the mode of action is by oxidative inactivation of the venom. *Schumanniophyton magnificum* is used extensively in African ethno-medicine for the treatment of various diseases and, most commonly, the treatment of snake bites [135]. Its structure is shown in Figure 19. Its source and biological activity are summarized in Table 16.

### 4.6. Hybrids of Chromones with Other Classes of Secondary Metabolites

This is an interesting category, as the chromone skeleton is conjugated to another high molecular weight compound, as shown in the following subclasses.

#### 4.6.1. Hybrids of Furano-Chromones with Cycloartane Triterpenes

**Figure 20:**
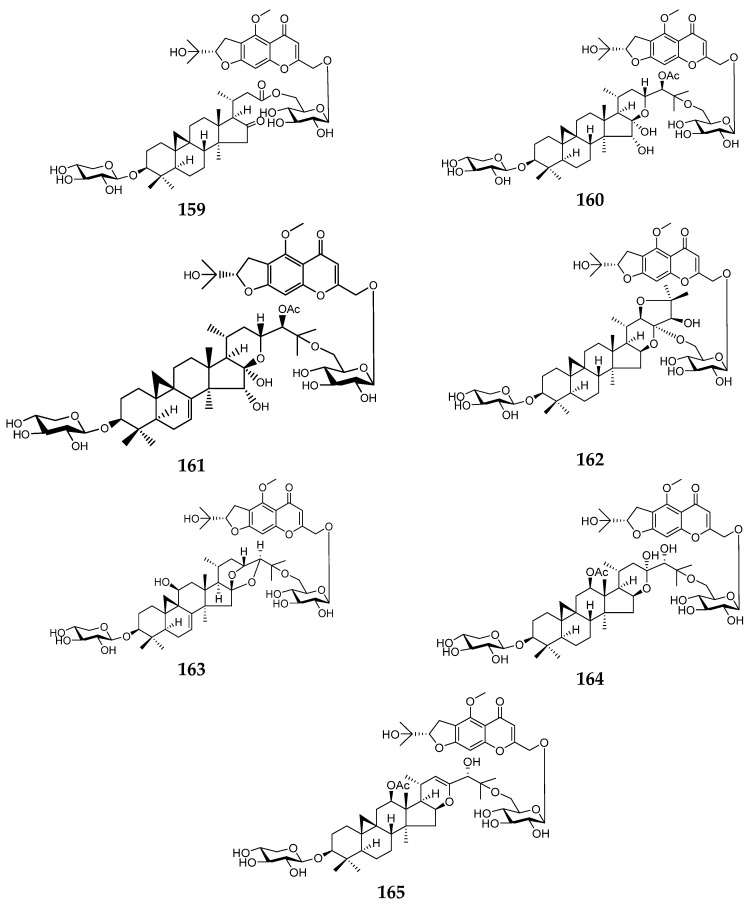
Structures of compounds **157**–**165**.

This subclass of compounds is a hybrid of cycloartane triterpene and chromone. The reported compounds were isolated from the rhizomes of *Cimicifuga foetida*. The compounds **157**–**165** are shown in Figure 20. The sources and the reported biological activities (if any) are summarized in Table 17.

#### 4.6.2. Hybrids of Chromones with Secoiridoids

**Figure 21:**
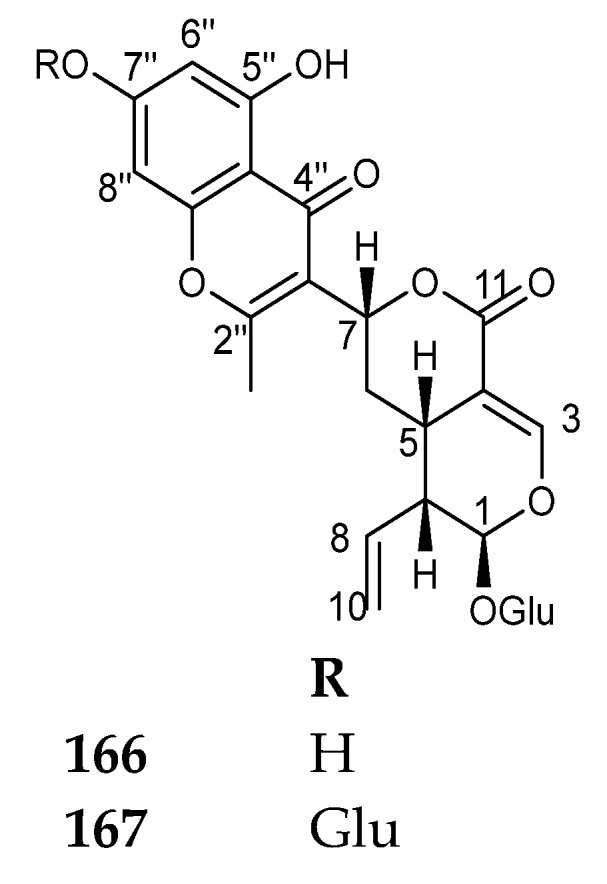
Structures of compounds **166**–**167**.

There are only two compounds (Figure 21) belonging to this class, sessilifoside (**166**) and 7″-*O*-*β*-d-glucopyranosylsessilifoside (**167**). Both compounds were isolated from the roots of *Neonauclea sessilifolia* roots [41]. The authors did not report biological activities for these compounds.

#### 4.6.3. Chromone Alkaloids Aminoglycosides

**Figure 22:**
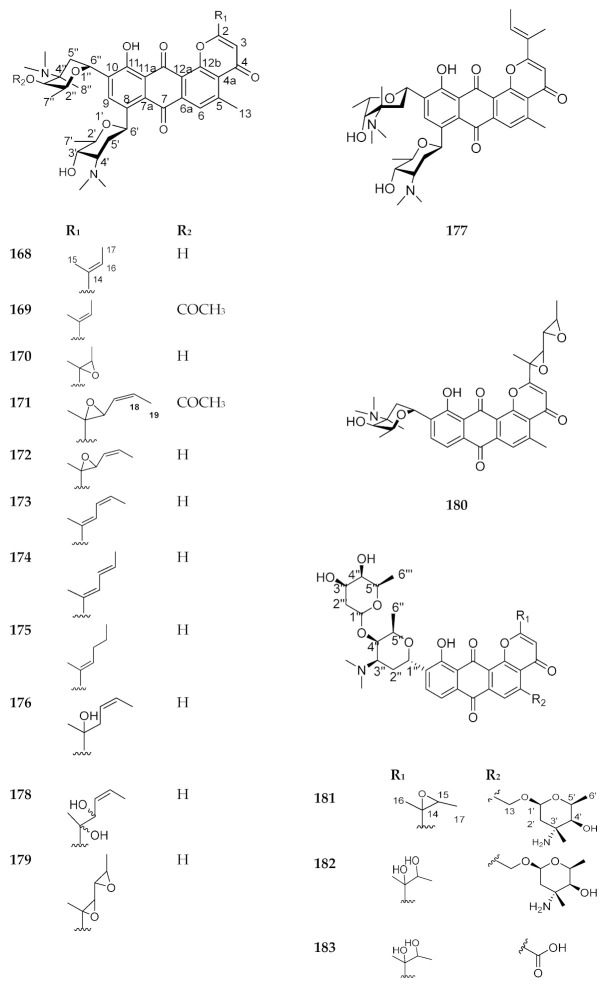
Structures of compounds **168**–**183**.

**Figure 23:**
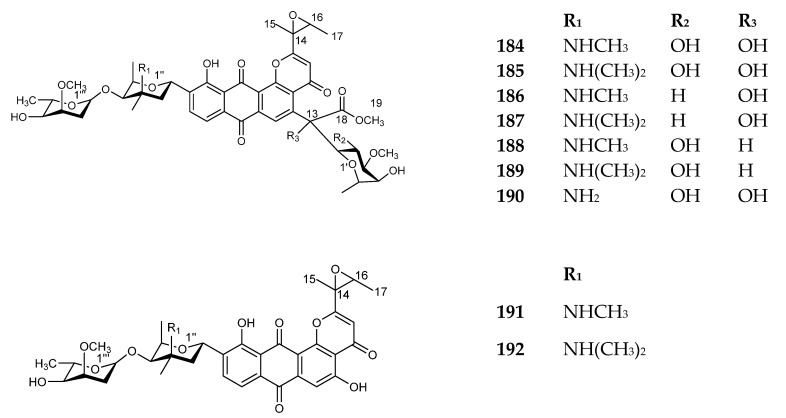
Structures of compounds **184**–**192**.

This category includes compounds **168**–**192**. Compounds **168**–**180** were reported from a strain of Streptomyces, isolated from a soil sample. These compounds showed antimicrobial activity against Gram-positive bacteria, as well as a potent antitumor activity. Conversely, compounds **181**–**183** were isolated from *Saccharothrix* species, while compounds **184**–**192** were reported from *Actinomycete* and exhibited antitumor and antimicrobial activities [137]. Compounds **168**–**192** are shown in Figure 22 and Figure 23. The sources and the reported biological activities (if any) are summarized in Table 18.

## 5. Spectroscopic Features

### 5.1. UV Features

Most of the published work on chromones show several strong bands in the range of 200–320 nm [150,151]. In contrast to chromone, the pyrone ring of 4-chromanone contains no double bond. The ultraviolet absorption spectra of chromones and chromanones are summarized in Table 19 [150].

The UV spectrum of chromones in alcohol shows two strong bands at λ_max_ 245 and 299 nm [152,153,154]. Some data reported three bands at λ_max_ 245, 303 and 297 nm [150]. 2-methyl-5,7-dihydroxy chromone shows bands at λ_max_ 250, 255, 295 and 325 nm, meanwhile 2-methyl-5-hydroxy-7-*O*-glycosyl chromone shows bands at λ_max_ 248, 255 and 290 nm [154]. The presence of an electron attracting group at C-2 resulted in a bathochromic shift in all bands [151]. The information gained from applying spectral shift reagents with flavonoids can be also applied to chromones. In the case of AlCl_3_, a bathochromic shift of 20–70 nm, which is non-reversible with acids, indicates a free hydroxyl group at position 5. Meanwhile, a bathochromic shift with NaOAc can be diagnostic for the presence of a free 7-hydroxyl group [154,155].

### 5.2. IR Features

**Carbonyl region:** The IR carbonyl stretching frequency for a chromone is observed at 1640~1660 cm^−1^, which is slightly higher than that of δ-pyrone (1650 cm^−l^) but is much lower than that of coumarins (1720–1740 cm^−l^) [25,153]. Despite that the OH group attached to C-5 of the chromone nucleus chelates strongly with the CO group, this intramolecular H-bonding has only a slight bathochromic effect on the CO stretching frequency [156]. All 5-hydroxychromones possess three significant maxima in the 1580–1700 cm^−1^ region. The two higher frequencies are intense at 1660 and 1630 cm^−1^, with a constant wavenumber separation of 34 ( ± 5) cm^−1^ in both carbon tetrachloride and chloroform.

**Hydroxyl region:** The IR hydroxyl stretching vibration for a chromone was observed at 2500–3650 cm^−1^. A strong chelation in 5-hydroxychromones does not produce a considerable bathochromic shifts in both the OH and CO stretching bands [156]. Chelated 5-hydroxychromones produce no absorption maxima in the 3300–3600 cm^−1^ region, but a weak absorption envelope extends from 2400 to 3300 cm^−1^. The entire envelope is associated with various stretching modes of the chelated 5-OH group [156]. For 7-hydroxychromones, a steric buttressing effect is observed when the 7-OH group is flanked by a bulky substituent in the ortho-position (6 or 8). The free OH band appears as a doublet centered at 3615 cm^−1^, the separation of the components being ~26 cm^−1^. When a prenyl moiety is located in the ortho-position to the 7-OH group, an intramolecular OH interaction occurs, resulting in two OH stretching frequencies. When a 7-OH group is flanked by an OMe group, intense intramolecularly bonded OH stretching frequencies are found at ~3513 and 3517 cm^−1^, respectively [156]. The 2-hydroxymethyl group exhibits a free stretching frequency at ≈3615 cm^−1^. At concentrations higher than 0.15 M, a broad-bonded OH frequency at 3400 cm^−1^ occurs due to intermolecular H-bonding, and it consequently disappears on dilution [156].

### 5.3. ^1^H-NMR Features

**Figure 24:**
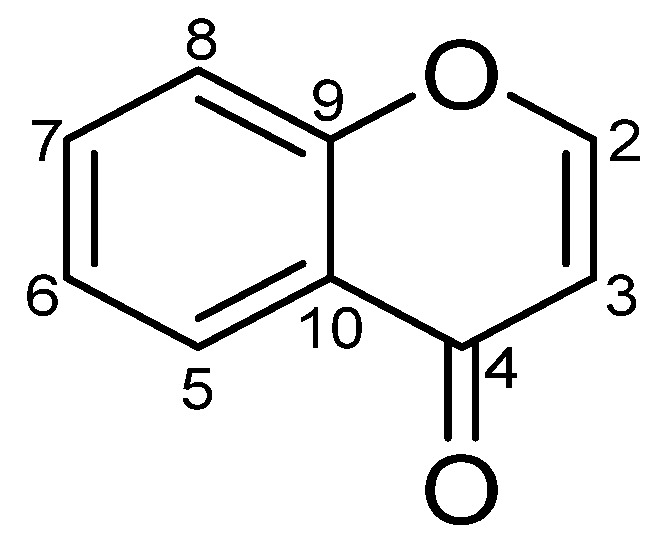
Basic skeleton of Chromone.

In the following text, we try to give insight about the most characteristic ^1^H-NMR features of the benzo-δ-pyrone skeleton (Figure 24) and its glycosides. The δ-pyrone ring has two olefinic protons assigned as H-2 and H-3. In 2, 3 unsubstituted chromones, for example compound **12,** the ^1^H-NMR spectrum shows two ortho-coupled doublets (*J =* 6.0 Hz), located downfield at δ_H_ 8.19 (H-2) and upfield at δ_H_ 6.26 (H-3) [25]. For 2-alkyl and 2-*O*-glycosyl chromones (compounds **21** and **1**, respectively), they are characterized by an upfield singlet proton (H-3) at δ_H_ 6.11 and 5.98, respectively [7,40]. Meanwhile, 3-alkyl and 3-*O*-glycosyl chromones (compounds **39** and **2**, respectively) are characterized by a downfield singlet proton (H-2) at δ_H_ 7.93 and 8.07, respectively [10,50].

**Chromanone glycosides** or 2,3-dihydrochromone glycosides are characterized by an oxygenated proton (H-2) at δ_H_ 4.12 and 5.44 as in compounds **62** [63] and **60 [50]**, respectively. The splitting pattern of H-2 can be either d, dd or ddd, depending on the number of neighboring protons. A small coupling constant between H-2 and H-3 (*J=* 2.8 Hz) can determine that they are located in the equatorial–equatorial position [50]. Further information on the detailed configuration can be clarified from observing NOESY correlations. Unsubstituted chromanones at C-3, as in **62,** show two geminal protons at δ_H_ 2.50 and 2.70. Their splitting pattern shows geminal (*J_3a_*_-*3b*_ = 16.2 Hz) and vicinal (*J_ax_*_-*eq*_ = 2.7 Hz or *J_ax_*_-*ax*_ = 12.6 Hz ) couplings [63]. In 3-alkyl substituted chromanones, as in **60** and **61**, H-3 is also detected at δ_H_ 2.79 [50].

Naturally occurring chromones often bear a hydroxyl or methoxy group at C-5 and/or C-7 and a methyl group at C-2 and/or C-5 [153]. The C-5 methyl is usually observed in 6-C and 8-C glycosides. In aprotic solvents such as DMSO-d_6_, the chelated 5-OH is detected as a singlet at δ_H_ 12.57; meanwhile, the 7-OH is detected at δ_H_ 10.00, as in compound **56 [31]**. The C-2 methyl in Schumaniofioside A **7** can be detected at δ_H_ 2.33 (3H, s) [17]. Meanwhile, those located at C-5, can be detected more downfield at δ_H_ 2.64 (3H, s) as in **79 [78]**.

For the phenyl part of the benzo-δ-pyrone skeleton, the protons show chemical shift and coupling constant values similar to those observed for protons in substituted benzenes.

**Sugar moiety:** Xylosyl, arabinosyl and glucosyl chromones show an anomeric proton signal at δ_H_ ~4.73 (d, *J =* 7–7.6 Hz) [10,11,12]. The former moieties can be differentiated by the number of the oxygenated protons at the δ_H_ 3-5 region, in addition to the difference in ^13^C-NMR values. Rhamnosyl chromones show a distinct signal at δ_H_ ~1.25 (3H, d, *J =* 6.0 Hz) corresponding to CH_3_-6’ of α-l-rhamnose [8]. The most abundant chromone of C-glycosides is the 8-C-glycoside form, followed by 6-C-glycosides. However, we encountered a unique 3-C-glycoside named macrolobin **64 [65]**. The anomeric proton in macrolobin is detected at δ_H_ 5.32 (d, *J =* 1.5 Hz), the small coupling constant being indicative of the α-anomer [65]. Biflorin **66** and isobiflorin **80**, as representative for 6-C and 8-C-glycosides, respectively, show the anomeric proton signal at δ_H_ 4.55 (d, *J =* 9.8 Hz), and 4.63 (d, *J =* 9.8 Hz), respectively [69]. The former coupling constant value is higher than that observed in case of O-glycosides (*J =* 7–7.6 Hz) [11,12]. In 5-*O*, 7-*O*, 6-C, furano-, pyrano-, oxepino-chromone glycosides, the sugar moiety can be further substituted with another sugar, as in **8**–**9, 25**–**32**, **78**–**79**, **141**–**142**, **150** and **153,** respectively. In 6-C, 8-C and, to a lesser extent, 7-*O*-glycosides, the sugar moiety can be mono- or disubstituted with a phenolic acid moiety, commonly at C-2’ or C-6’ or C-2’ and C-3’. The most commonly occurring phenolic moiety is gallic acid, but other phenyl propanoids such as cinnamoyl, coumaroyl, feruloyl and coniferoyl moieties also exist. The galloyl moiety is characterized by a singlet aromatic signal integrated for two protons at δ_H_ 6.75 [27]. The cinnamoyl moiety is confirmed by two trans-coupled olefinic protons at δ_H_ 7.43 (d, *J =* 15.8 Hz) and 6.25 (d, *J =* 15.8 Hz), in addition to the aromatic signals of the benzene ring [30]. The presence of coumaroyl substitution is characterized by AA’ BB’ system for two pairs of ortho-coupled aromatic protons at δ_H_ 7.31 (2H, d, *J =* 8.6 Hz) and δ_H_ 6.74 (2H, d, *J* = 8.6 Hz), a trans-olefinic proton signals at δ_H_ 7.35 (1H, d, *J =* 16.1 Hz) and δ_H_ 6.03 (1H, d, *J =* 15.9 Hz) [29].

**Prenyl and Isoprenyl chromone glycosides:** In the case of 7-*O*-glycoside **127**, hydroxyl prenyl moiety can be easily characterized by the allylic methylene protons at δ_H_ 3.53 (2H, m, H-1’), an olefinic proton at δ_H_ 5.39 (t, *J =* 6.4, H-2’), oxygenated methylene protons δ_H_ 4.20 and 4.46 (1H each, d, *J* = 12.0 Hz, H-4’), and an olefinic methyl at δ_H_ 1.75 (3H, d, *J =* 1.2, H-5″) [60]. Its isomeric 4′-*O*-glycoside **126** showed similar signals; however, the hydroxyl methylene protons (H-4’) were slightly downfield at δ_H_ 4.34 and 4.65 due to *O*-glycosidation [106]. Meanwhile, the hydroxyl isoprenyl group in the 7-*O*-glycoside **130** is characterized by the methylene protons at δ_H_ 2.96 (2H, m, H-1’), an oxygenated methine proton at δ_H_ 4.35 (H-2’, m), exomethylene protons at δ_H_ 4.74 and 4.91 (H-4’) and an olefinic methyl at δ_H_ 1.87 (3H, s, H-5’). Its isomeric 2′-*O*-glycoside **129** showed similar signals, but the oxygenated methine proton (H-2’) is shifted downfield at δ_H_ 4.74 due to *O*-glycosidation [60].

**Phenyl ethyl chromone glycosides:** The presence of the phenyl ethyl moiety in compound **136** can be detected by the methylene proton signals at δ_H_ 2.75 (2H each, dd, *J* = 14.8, 6.4 Hz, H-7’) and 3.19 (2H each, *J =* 14.8, 3.1 Hz, H-8’), in addition to the aromatic protons of the phenyl ring. The 8′-hydroxy phenyl ethyl moiety in **135** shows an oxygenated proton signal δ_H_ 5.85 (dd, *J =* 3.5, 5.9 Hz, H-8’) [110].

### 5.4. ^13^C-NMR Features

**Figure 25:**
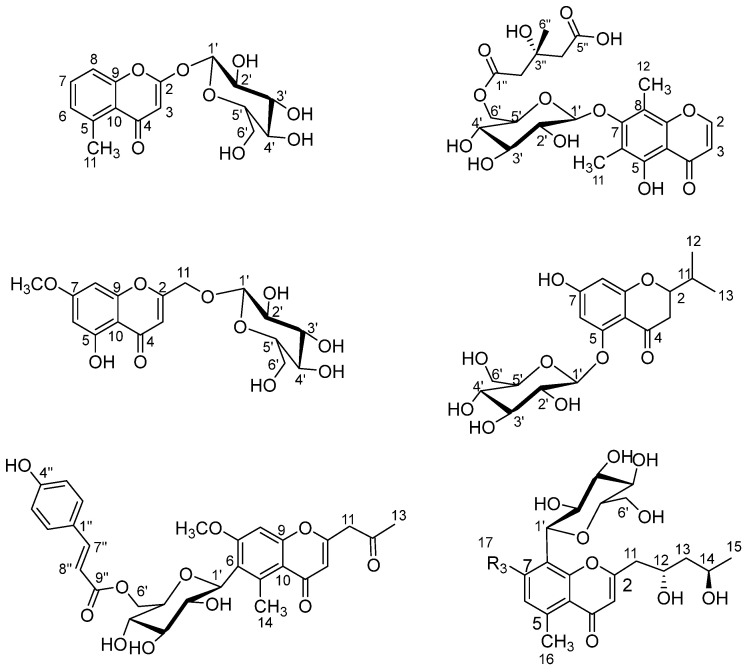
Representative guide figure for numbering of chromone glycosides attached to different substituents.

For better understanding of the differences in chemical shifts related to the substituents on the chromone moiety, we preferred to add the ^13^C-NMR data in Table 20, Table 21, Table 22, Table 23, Table 24, Table 25, Table 26, Table 27, Table 28, Table 29, Table 30, Table 31, Table 32, Table 33, Table 34, Table 35, Table 36, Table 37, Table 38, Table 39 and Table 40. For the numbering of the skeleton, the following figure (Figure 25) gives few examples for the numbering system of the skeleton with multiple substituents. Briefly, the basic chromone nucleus was assigned numbers 1–10. In the case of a substitution at C-2, numbers 11, 12…etc. were given to the substituents, followed by substitution at C-3 and so on. Sugar moiety, and substituents attached to it, were assigned numbers 1’, 2’, … and then 1″, 2″, … etc. For better understanding, the following figure shows representative examples for the numbering system. Some complicated structures have their own numbering system, shown on them within the review.

## 6. Conclusions

Chromone glycosides are one of the most important classes of secondary metabolites. In this review, we summarized 192 naturally occurring chromone glycosides with their sources, reported activities, and spectroscopic features. Basically, they were categorized into several classes: chromone-*O*-glycosides including compounds **1**–**59**, among them, four chromanone glycosides (**60**–**63**), chromone-*C*-glycosides including compounds **64**–**122**, prenyl and isoprenyl chromone glycosides including compounds **123**–**134**, phenyl ethyl chromone glycosides including compounds **135**–**139**, furano-chromone glycosides including compounds **140**–**148**, pyrano-chromone glycosides including compounds **149**–**151**, oxepino-chromone glycosides including compounds **152**–**155**, Pyrido-chromone glycoside including compound **156**, furanochromones with cycloartane triterpenes including compounds **157**–**165**, glycoside derivatives of chromones with secoiridoids including compounds **166** and **167**, and chromone alkaloids aminoglycosides including compounds **168**–**192**. Diverse bioactivities were discovered for most of the reported chromone glycosides. Several chromone glycosides show potent biological activities as anti-viral, acetylcholinesterase inhibition, anti-tumor, anti-inflammatory, etc. This review directs the attention for further deep investigation of chromone glycosides for drug discovery.

## Figures and Tables

**Table 1:** The distribution of chromone glycosides reported through this review.

		Family	Genus	Species	Compounds
Plants (Angiosperms)	1	Ericaceae	*Rhododendron*	*ovatum*	**2**
				*spinuliferum*	**3**
				*collettianum*	**55**
			*Calluna*	*vulgaris*	**13**
	2	Rubiaceae	*Schumanniophyton*	*magnificum*	**7**, **25**, **156**
			*Knoxia*	*corymbosa*	**20**, **24**, **35**, **36**
			*Adina*	*rubescens*	**20**
			*Neonauclea*	*sessilifolia*	**26**, **166**
	3	Amaryllidaceae	*Gethyllis*	*ciliaris*	**10**
			*Pancratium*	*biflorum*	**20**, **66**
				*maritimum*	**20**, **47**
	4	Polygonaceae	*Polygonum*	*capitatum*	**15**
			*Rheum*	*austral*	**50**
				sp.	**52**, **53**
	5	Apiaceae	*Ammi*	*visnaga*	**20**, **140**, **146**
			*Peucedanum*	*austriacum*	**20**
				*japonicum*	**149**
			*Cnidium*	*monnieri*	**57**, **58**, **123**–**130**
				*juponicum*	**123**, **124**
			*Bupleurum*	*chinense*	**58**
			*Angelica*	*archangelica*	**125**
				*genuflexa*	**146**, **149**
				*japonica*	**146**, **149**
			*Archangelica*	*litoralis*	**125**
			*Saposhnikovia*	*divaricata*	**143**–**146**, **149**, **150**
			*Ledebouriella*	*seseloides*	**144**
			*Diplolophium*	*buchananii*	**144**, **146**, **151**
			*Sphallerocarpus*	*gracilis*	**144**
			*Glehnia*	*littoralis*	**149**
	6	Hypericaceae	*Hypericum*	*henryi*	**22**, **23**
				*erectum*	**22**, **38**
				*sikokumontanum*	**38**, **39**, **60**, **61**
				*japonicum*	**82**, **83**
	7	Ranunculaceae	*Delphinium*	*hybridum*	**28**
			*Cimicifuga*	*heracleifolia*	**141**
				*foetida*	**146**, **147**, **157**–**165**
			*Eranthis*	*hyemalis*	**131**, **132**, **152**, **153**, **154**
				*cilicica*	**133**, **134**, **153**, **155**
	8	Myrtaceae	*Myrtus*	*communis*	**34**
			*Syzygium*	*aromaticum*	**66**, **71**, **80**, **86**
			*Baeckea*	*frutescens*	**66**, **67**, **70**, **72**, **85**, **87**
			*Kunzea*	*ambigua*	**67**, **70**, **72**–**74**, **80**, **85**, **88**, **89**
			*Eucalyptus*	*globulus*	**80**
				*maidenii*	**82**, **83**
				*grandis*	**83**
				*urograndi*	**83**
	9	Fabaceae	*Cassia*	*multijuga*	**51**, **77**
				*siamea*	**59**
				*obtusifolia*	**68**, **69**
				*spectablis*	**78**
				*obtusifolia*	**84**
			*Macrolobium*	*latifolium*	**64**
			*Aspalathus*	*linearis*	**65**
			*Abrus*	*mollis*	**80**
			*Ononis*	*vaginalis*	**135**
	10	Asphodelaceae	*Aloe*	*vera*	**75**, **76**, **90**–**98**, **103**, **104**–**110**, **112**–**117**, **120**–**122**
				*barbadensis*	**97**, **98**, **103**, **107**, **108**
				*rupestris*	**99**
				*cremnophila*	**101**
				*nobilis*	**111**, **118**, **119**
	11	Araceae	*Scindapsus*	*officinalis*	**18**–**20**, **29**, **31**, **32**, **56**, **57**
	12	Asteraceae	*Mutisia*	*acuminate*	**1**
	13	Eucryphiaceae	*Eucryphia*	*cordifolia*	**4**
	14	Saxifragaceae	*Astilbe*	*thunbergii*	**4**
	15	Smilacaceae	*Smilax*	*glabra*	**4**
	16	Pentaphylaceae	*Eurya*	*japonica*	**5**
	17	Salicaceae	*Salix*	*matsudana*	**6**
	18	Meliaceae	*Dysoxylum*	*binectariferum*	**7**
	19	Euphorbiaceae	*Acalypha*	*fruticose*	**7**
	20	Staphyleaceae	*Staphylea*	*bumalda*	**7**
	21	Amaranthaceae	*Salicornia*	*europaea*	**12**
	22	Aquifoliaceae	*Ilex*	*hainanensis*	**13**
	23	Rosaceae	*Dasiphora*	*parvifolia*	**16**, **17**
	24	Bignoniaceae	*Tecomella*	*undulata*	**20**, **27**
	25	Olacaceae	*Scorodocarpus*	*borneensis*	**26**
	26	Pinaceae	*Pseudotsuga*	*sinensis*	**37**
	27	Selaginellaceae	*Selaginella*	*uncinata*	**46**, **48**
	28	Gentianaceae	*Swertia*	*punicea*	**54**
	29	Cannabaceae	*Humulus*	*lupulus*	**62**
	30	Euphorbiaceae	*Chrozophora*	*prostrata*	**79**
	31	Cucurbitaceae	*Cucumis*	*melo*	**136**
	32	Thymelaeaceae	*Aquilaria*	*sinensis*	**137**, **139**
	33	Poaceae	*Imperata*	*cylindrical*	**138**
Ferns	1	Polypodiaceae	*Drynaria*	*fortunei*	**7**, **28**, **30**, **32**, **33**, **57**
	2	Dryopteridaceae	*Dryopteris*	*fragrans*	**20**, **21**
	3	Onocleaceae	*Matteuccia*	*intermedia*	**49**
Lichens			*Roccellaria*	*mollis*	**41**, **42**, **45**
			*Schismatomma*	*accedens*	**41**, **42**, **45**
			*Roccella*	*galapagoensis*	**41**, **42**, **45**
			*Lobodirina*	*cerebriformis*	**44**
Fungi			*Armillaria*	*tabescens*	**11**
			*Orbiocrella*	sp.	**40**, **43**
			*Stemphylium*	*botryosum*	**63**
Actinobacteria			*Streptomyces*	*phaeoverticillatus* var. *takatsukiensis*	**168**
				*pluricolorescens*	**169**–**171**
				sp.	**172**–**178**, **180**
				*griseoruber*	**179**
			*Saccharothrix*	sp.	**181**–**183**
			*Actinomycete*		**184**–**192**

**Table 2:** 3-*O*-Chromone glycosides with their sources and biological activities.

No.	Compound	Source	Biological Activity
**2**	3,5,7-trihydroxychromone-3-*O*- *β*-d-xylopyranoside	*Rhododendron ovatum* roots [10] *Eurya japonica* stems [11]	Inhibitory effects on LPS (Lipopolysaccharide)-induced NO (Nitric Oxide) production with inhibition rate 36.24 ± 1.29% at 20 μg/mL [11]
**3**	3,5,7-trihydroxylchromone-3-*O*- α-l-arabinopyranoside	*Rhododendron spinuliferum* aerial parts [12]	Inhibition of NO production in LPS-stimulated RAW 264.7 cells with an IC_50_ value more than 100 mM [12]
**4**	Eucryphin (5,7-dihydroxy-3-(α-*O*-l-rhamnopyranosyl)- 4H-l-benzopyran-4-one)	*Eucryphia cordifolia* bark [8] *Astilbe thunbergii* rhizomes [13]	Norepinephrine-enhancing lipolytic effect 6.432 ± 0.014 FFA µmol/mL at 1000 µg [13] Enhancing effect on burn wound repair at 100 mg ointment per mouse [14]
	Smiglanin (3,5,7-trihydroxychromone-3-*O*- α-l-rhamnopyranoside)	*Smilax glabra* roots [9]	No reported biological activity
**5**	5,7-Dihydroxy-4H-chromen-4-one- 3-*O*-*β*-d-glucopyranoside	*Eurya japonica* stems [11]	Inhibitory effects on LPS-induced NO production with inhibition rate 53.79 ± 1.78% at 20 μg/mL [11]

**Table 3:** 5-*O*-Chromone glycosides with their sources and biological activities.

No.	Compound	Source	Biological Activity
**6**	Matsudoside A (5-*β*-d-glucosyloxy-7- hydroxychromone)	*Salix matsudana* leaves [15]	No reported biological activity
**7**	Schumaniofioside A (2-methyl-5,7-dihydroxychromone 5-*O*-*β*-d-glucopyranoside)	*Schumanniophyton magnificum* root bark [16] *Dysoxylum binectariferum* fruits [17]. *Acalypha fruticose* aerial parts [18,19] *Drynaria fortune* rhizomes [13]	Inhibition of proinflammatory cytokines TNF-α (39.51 ± 1.21%) and IL-6 (22.21 ± 0.58%) at 5 μM [17] Inhibition of NF-kB transcriptional activity and iNOS with IC_50_ value of 29.5 ± 6.5 µg/mL [19]
**8**	Staphyloside A	*Staphylea bumalda* leaves [20]	No reported biological activity
**9**	Staphyloside B		
**10**	Isoeugenitol glucoside	*Gethyllis ciliaris* underground parts [21]	No reported biological activity

**Table 4:** 7-*O*-Chromone glycosides with their sources and biological activities.

No.	Compound	Source	Biological Activity
**12**	7-*O*-*β*-d-glucopyranosyl-6-methoxychromone	*Salicornia europaea* leaves and stems [25]	No reported biological activity
**13**	5,7-dihydroxychromone 7-*O*-*β*-d-glucopyranoside	*Ilex hainanensis* leaves [26] *Calluna vulgaris* flowers [27]	No reported biological activity
**14**	5,7-dihydroxychromone 7-*O*-*β*-d-glucuronide methyl ester	*Davallia mariesii* rhizomes [28]	No reported biological activity
**15**	7-*O*-(6′-galloyl)-*β*-d-glucopyranosyl-5-hydroxychromone	*Polygonum capitatum* aerial parts [29]	No reported biological activity
**16**	5-hydroxy-7-*O*-(6-*O*-*p*-*cis*-coumaroyl-*β*-d-glucopyranosyl)-chromone	*Dasiphora parvifolia* aerial parts [30]	No reported biological activity
**17**	5-hydroxy-7-*O*-(6-*O*-*p*-*trans*-coumaroyl-*β*-d-glucopyranosyl)-chromone	*Dasiphora parvifolia* aerial parts [30]	No reported biological activity
**18**	Officinaliside A	*Scindapsus officinalis* stems [31]	No reported biological activity
**19**	7-*O*-*α*-l-rhamnosyl-nereugenin	*Scindapsus officinalis* stems [31]	No reported biological activity
**20**	Undulatoside A (2-methyl-5,7-dihydroxychromone 7-*O*-*β*-d-glucopyranoside)	*Scindapsus officinalis* stems [31] *Ammi visnaga* fruits [32] *Knoxia corymbosa* [33] *Pancratium biflorum* roots [34] *Panacratium biflorum* flowering bulbs [35] *Pancratium maritimum* L. fresh bulbs [36] *Peucedanum austriacum* [37] *Tecomella undulata* bark [38] *Adina rubescens* leaves [39] *Dryopteris fragrans* [40] *Staphylea bumalda* leaves [20]	Immunomodulatory activity inhibited the proliferation of murine B lymphocytes in vitro at 10^−5^ M [33] Inhibition of nitric oxide production in lipopolysaccharide induced RAW 264.7 macrophages with an IC_50_ value of 49.8 μM [40] Weak antimigratory activity against human metastatic prostate cancer cells (PC-3M) at 50 µM [36]
**21**	Frachromone C (5-hydroxy-2-ethylchromone-*7*-*O*-*β*-d-glucopyranoside)	*Dryopteris fragrans* whole plant [40]	Inhibition of nitric oxide production in lipopolysaccharide induced RAW 264.7 macrophages with an IC_50_ value of 45.8 μM [40]
**22**	Urachromone A (5-hydroxy-2-isopropylchromone-7-*O*-*β*-d-glucopyranoside)	*Hypericum henryi* aerial parts [41] *Hypericum erectum* [42]	No reported biological activity
	Hyperimone A		
**23**	Urachromone B	*Hypericum henryi* aerial parts [41]	No reported biological activity
**24**	Corymbosin K_2_ (7-*O*-*β*-d-6- acetylglucopyranosyl-5-hydroxy-2-methylchromone)	*Knoxia corymbosa* [33]	Immunomodulatory activity inhibited the proliferation of murine B lymphocytes in vitro at 10^−5^ M [33]
**25**	Schumanniofioside B	*Schumanniophyton magnificum* root bark [16]	No reported biological activity
**26**	5-hydroxy-2-methylchromone-7-*O*-*β*-d-apiofuranosyl-(1→6)- *β*-d-glucopyranoside	*Neonauclea sessilifolia* roots [43] *Scorodocarpus borneensis* leaves [44] *Staphylea bumalda* leaves [20]	No reported biological activity
**27**	Undulatoside B	*Tecomella undulata* [45]	No reported biological activity
**28**	2-methyl-chromone-5,7-diol 7-*O*-*α*-l-rhamnopyranosyl-(1-6)-*β*-d-glucopyranoside	*Delphinium hybridum* aerial parts [46] *Drynaria fortunei* rhizomes [22]	No reported biological activity
**29**	Officinaliside C (7-*O*-[*β*-d-glucopyranosyl-(1-2)-*α*-l-rhamnopyranosyl]-5-hydroxy-2-methyl-4H-1-benzopyran-4-one)	*Scindapsus officinalis* stems [31]	No reported biological activity
**30**	Drynachromoside C (5-hydroxy-2-methyl chromone-7-*O*-*β*-d-glucopyranosyl (1-3)-*α*-l-rhamnopyranoside)	*Drynaria fortunei* rhizomes [22]	Inhibitory activity on triglyceride accumulation at 10 μM [22]
**31**	Officinaliside B (7-*O*-[6-acetyl-*β*-d-glucopyranosyl-(1-3)-*α*-l-rhamnopyranosyl]-5-hydroxy- 2-methyl-4H-1-benzopyran-4-one)	*Scindapsus officinalis* stems [31]	Inhibition of NO production in LPS-stimulated RAW 264.7 cells with an IC_50_ value of 16.1 μM [31]
**32**	Drynachromoside A (5-hydroxy-2-methyl-4H-benzopyran-4-one-7-*O*-*β*-d-glucopyranosyl-(1-4)-*α*-l-rhamnopyranoside)	*Scindapsus officinalis* stems [31] *Drynaria fortunei* rhizomes [47]	Proliferative activity 10.1% on MC3T3-E1 (Mouse osteoblast) cells at 25 μg/mL [47]
**33**	Drynachromoside D (5-hydroxy-2-methyl chromone-7-*O*-*α*-l-arabinopyranosyl(1-2)-*β*-d-glucopyranosyl(1-4)-*α*-l-rhamnopyranoside)	*Drynaria fortunei* rhizomes [22]	Inhibitory activity on triglyceride accumulation (inhibited PPARγ, C/EBPα and aP2 expression by 50%, 43% and 37% at 10 mM) [22]
**34**	Undulatoside A 6′-*O*-gallate	*Myrtus communis* leaves [48]	
**35**	Corymbosin K3 (7-*O*-[6-*O*-(4-*O*-*trans*-caffeoyl-*β*-d-allopyranosyl)]-*β*-d-glucopyranosyl-5-hydroxy-2- methylchromone)	*Knoxia corymbosa* [33]	Immunomodulatory activity inhibited the proliferation of murine B lymphocytes in vitro at 10^−5^ M [33]
**36**	7-*O*-[6-*O*-(4-*O*-*trans*-feruloyl-*β*-d-allopyranosyl)]- *β*-d-glucopyranosyl-5-hydroxy-2-methylchromone	*Knoxia corymbosa* [33]	No reported biological activity
**37**	5-hydroxy-6-methylchromone-7-*O*-*β*-d-glucopyranoside	*Pseudotsuga sinensis* [49]	No reported biological activity
**38**	Takanechromone A (5,7-dihydroxy-3-methylchromone- 7-*O*-*β*-d-glucopyranoside)	*Hypericum sikokumontanum* aerial parts [50] *Hypericum erectum* [42]	No reported biological activity
	Hyperimone B		
**39**	Takanechromone B (5,7-dihydroxy-3-ethylchromone- 7-*O*-*β*-d-glucopyranoside)	*Hypericum sikokumontanum* aerial parts [50]	No reported biological activity
**40**	7-*O*-(4-*O*-Methyl-*β*-d-glucopyranosyl)eugenitol	The scale-insect pathogenic fungus *Orbiocrella* sp. [23]	No reported biological activity
**41**	Mollin	Lichens (*Roccellaria mollis*, *Schismatomma accedens*, *Roccella galapagoensis*) [51]	No reported biological activity
**42**	Roccellin	Lichens (*Roccellaria mollis*, *Schismatomma accedens*, *Roccella galapagoensis*) [51]	No reported biological activity
**43**	7-*O*-(4-*O*-Methyl-*β*-d-glucopyranosyl)isoeugenitol	The scale-insect pathogenic fungus *Orbiocrella* sp. [23]	No reported biological activity
**44**	Lobodirin	*Lobodirina cerebriformis* lichen [51]	No reported biological activity
**45**	Galapagin	Lichens (*Roccellaria mollis*, *Schismatomma accedens*, *Roccella galapagoensis*) [51]	No reported biological activity
**46**	Uncinoside A (5-hydroxy-2,6,8- trimethylchromone 7-*O*-*β*-d-glucopyranoside)	*Selaginella uncinate* Herb [24]	Antiviral activity against respiratory syncytial virus (RSV) with an IC_50_ value of 6.9 μg/mL, against parainfluenza type 3 virus (PIV 3) with an IC_50_ value of 13.8 μg/mL [24]
**47**	Pancrichromone	*Pancratium maritimum* L. fresh bulbs [36]	No reported biological activity
**48**	Uncinoside B (5-acetyoxyl-2,6,8-trimethylchromone 7-*O*-*β* -d-glucopyranoside)	*Selaginella uncinate* herb [24]	Antiviral activity against respiratory syncytial virus (RSV) with an IC_50_ value of 1.3 μg/mL, against parainfluenza type 3 virus (PIV 3) with an IC_50_ value of 20.8 μg/mL [24]
**49**	Matteuinterin B	*Matteuccia intermedia* rhizomes [52]	
**50**	2,5-dimethylchromone-7-*O*-*β*-d-glucopyranoside	*Rheum austral* D. Don underground parts [53] *Rumex gmelini* Turcz. roots [54]	Anti-oxidant activity (DPPH radical scavenging capacity with an IC_50_ value of 66.9 ± 1.3 μM) [53]
**51**	5-acetonyl-7-*β*-d-glucopyranosyl-2-methylchromone	*Cassia multijuga* leaves [55,56]	No reported biological activity
**52**	2-methyl-5-(2′-oxo-4′-hydroxyphenyl)-7-hydroxychromone 7-*O*-*β*-d-glucopyranoside	Chinese rhubarb (Rhei Rhizoma) [57]	No reported biological activity
**53**	Aloesone 7-*O*-*β*-d-glucopyranoside	Chinese rhubarb (Rhei Rhizoma) [57]	No reported biological activity

**Table 5:** 8-*O*-Chromone glycosides with their sources and biological activities.

No.	Compound	Source	Biological Activity
**54**	8-*O*-*β*-d-Glucopyranosyl-2-methylchromone	*Swertia punicea* whole herb [58]	No reported biological activity
**55**	8-*O*-*β*-d-Glucopyranosyl-6-hydroxy-2-methyl-4H-1-benzopyrane-4-one	*Rhododendron collettianum* aerial parts [59]	Inhibitory activity against tyrosinase enzyme with an IC_50_ value of 256.97 μM [59]

**Table 6:** 11, 13-*O*-chromone glycosides with their sources and biological activities.

No.	Compound	Source	Biological Activity
**56**	Officinaliside D (2-hydroxymethyl-5,7-dihydroxy-4H-benzopyran-4- one-1′-*O*-α-l-arabinopyranoside)	*Scindapsus officinalis* stems [31]	Inhibition of NO production in LPS-stimulated RAW 264.7 cells with an IC_50_ value of 19.1 µM [31]
**57**	Drynachromoside B	*Drynaria fortune* rhizomes [22,47] *Scindapsus officinalis* stems [31]	Mild inhibitory activity against MC3T3-E1 (mouse osteoblast) cells at 3.125 to 100 μg/ml [47] Triglyceride accumulation inhibitory effect at 0.1 to 10 μM [22]
	Monnieriside A	*Cnidium monnieri* fruits [60]	No reported biological activity
**58**	Saikochromoside A	*Bupleurum chinense* [61] *Cnidium monnieri* fruits [60]	No reported biological activity
**59**	2-Methyl-5-propyl-7,12- dihydroxychromone-12-*O*-*β*-d-glucopyranoside	*Cassia siamea* stem [62]	No reported biological activity

**Table 7:** Chromanone glycosides with their sources and biological activities.

No.	Compound	Source	Biological Activity
**60**	Takanechromanone A	*Hypericum sikokumontanum* aerial parts [50]	Anti-*Helicobacter pylori* at 100 µg/disc [50]
**61**	Takanechromanone B		
**62**	5-*β*-d-glucopyranosyloxy-7-hydroxy-2-isopropyl-chromanone	*Humulus lupulus* L. bracts [63]	No reported biological activity
**63**	Stemphylin (3-hydroxy-2, 2-dimethyl-5-α-d-glucopyranoside-2, 3-dihydrochromone)	The liquid culture of the fungus *Stemphylium botryosum* [64]	Phytotoxic activity [64]

**Table 8:** 3-C-Chromone glycoside with its source and biological activities.

No.	Compound	Source	Biological Activity
**64**	Macrolobin (5,7-dihydroxychromone- 3α-d-C-glucoside)	*Macrolobium latifolium* aerial parts [65]	Inhibition of acetylcholinesterase enzyme with an IC_50_ value of 0.8 μM Antimicrobial activity against *P. aeruginosa* and *Salmonella* at 0.73 and 0.44 μM, respectively [65]

**Table 9:** 6-C-Chromone glycosides with their sources and biological activities.

No.	Compound	Source	Biological Activity
**65**	5,7-dihydroxy-6-C-glucosyl-chromone	*Aspalathus linearis* fermented rooibos (red-brownish dry leaves) [66]	No reported biological activity
**66**	Biflorin (6-*β*-C-glucopyranosyl-5,7-dihydroxy-2-methylchromone)	*Pancratium Biflorum* roots [34] *Syzygium aromaticum* L. flower buds [67,68] *Baeckea frutescens* leaves [69,70]	Inhibitory activity to phosphodiesterase and spared cyclic nucleotides at 10^−9^ M [34] Inhibition of LPS-induced production of nitric oxide (NO) and prostaglandin E_2_ (PGE2) in RAW 264.7 macrophages with IC_50_ values of 51.7 and 37.1 μM, respectively [67]
**67**	6-*β*-C-glucopyranosyl- 5,7-dihydroxy-2-isopropylchromone	*Baeckea frutescens* leaves [69,70] *Kunzea ambigua* leaves [71]	Inhibitory activity 70.4% against EBV-EA (Epstein–Barr virus early antigen) activation induced by 12-*O*-tetradecanoylphorbol 13-acetate (TPA) at 500 mol ratio/TPA [71]
**68**	Obtusichromoneside C	*Cassia obtusifolia* seeds [72]	Weak inhibitory activity against human organic anion/cation transporters (OATs/OCTs) and organic anion transporting polypeptides (OATPs) at 50 μM [72]
**69**	Obtusichromoneside A		
**70**	Kunzeachromone C	*Kunzea ambigua* leaves [71] *Baeckea frutescens* leaves [70]	Inhibition of copper-induced LDL oxidation with an IC_50_ value of 3.35 ± 0.36 μM [70]
**71**	6-C-*β*-d-(6′-*O*-galloyl)glucosylnoreugenin	*Syzygium aromaticum* flower buds [68,73]	Cytotoxicity against human ovarian cancer cells (A2780) with an IC_50_ value of 66.78 ± 5.49 μM [68] Prolyl endopeptidase inhibitory effects with an IC_50_ value of 1.74 ± 0.03 μM [73]
**72**	6-*β*-C-(2′-*O*-galloylglucopyranosyl)-5,7-dihydroxy- 2-isopropylchromone	*Baeckea frutescens* leaves [69,70] *Kunzea ambigua* leaves [71]	Inhibitory activity 68.4% against EBV-EA activation induced by TPA at 500 mol ratio/TPA [71] Inhibition of copper-induced LDL oxidation with an IC_50_ value of 3.90 ± 0.24 μM [70]
**73**	Kunzeachromone D	*Kunzea ambigua* leaves [71]	No reported biological activity
**74**	Kunzeachromone A		
**75**	Aloeveraside B	*Aloe vera* resin [74,75,76]	Inhibition of urease enzyme (55% and 62%, respectively) at 1 mg/mL concentration, significant growth inhibition (70.5 and 76.4%) of the breast cancer cell line MDA-MB-231 at 100 μM, and antioxidant (80% and 60%) at 1 mg/mL [74] Anti-lipid peroxidation activity with IC_50_ values of 432.1 ± 0.6 and 469.5 ± 0.4 µmol/L, respectively [75]
**76**	Aloeveraside A		
**77**	Acetonyl-6-glycosyl -7-hydroxy -2-methylchromone	*Cassia multijuga* leaves [55,56]	No reported biological activity
**78**	5-acetonyl-7-hydroxy-6-C-glucopyranosyl-2-methyl chromone 2″-*O*-glucopyranoside	*Cassia spectablis* seeds [77]	No reported biological activity
**79**	2-acetonyl-5-methyl-7-hydroxy-6-C-glucopyranosyl chromone 2″-*O*-glucopyranoside	*Chrozophora prostrata* roots [78]	No reported biological activity

**Table 10:** 8-C-Chromone glycosides with their sources and biological activities.

No.	Compound	Source	Biological Activity
**80**	Isobiflorin	*Abrus mollis* Hance. aerial parts [83] *Syzygium aromaticum* L. flower buds [67] *Kunzea ambigua* (SM.) Druce. leaves [71] *Eucalyptus globulus* leaves [84,85] *Eugenia caryophyllata* flower buds [86]	Inhibition of LPS-induced production of nitric oxide (NO) with an IC_50_ > 60 μM and prostaglandin E2 (PGE2) ) with an IC_50_ value of 46.0 μM [67]
**81**	7-methoxy-isobiflorin	Zhuyeqing Liquor; a famous traditional Chinese functional health liquor [87]	No reported biological activity
**82**	5,7-Dihydroxy-2-isopropylchromone- 8-*β*-d-glucoside	*Hypericum japonicum* aerial parts [71,88,89] *Eucalyptus maidenii* bark [84]	Inhibit Epstein–Barr virus early antigen induced by 12-*O*-tetradecanoylphorbol 13-acetate (TPA) in Raji cells (70.4%) at 500 mol ratio/TPA [71]
**83**	5,7-Dihydroxy-2-(1-methylpropyl) chromone-8-*β*-d-glucoside	*Hypericum japonicum* aerial parts [71,88] *Eucalyptus grandis*, *Eucalyptus urograndi*, and *Eucalyptus maidenii* bark [90]	No reported biological activity
**84**	Obtusichromoneside B	*Cassia obtusifolia* seeds [72]	Inhibitory activity against human organic anion/cation transporters (OATs/OCTs) and organic anion transporting polypeptides (OATPs) at 50 μM [72]
**85**	Kunzeachromone E [8-*β*-C-(2′-galloylglucopyranosyl)- 5,7-dihydroxy-2-methylchromone]	*Kunzea ambigua* leaves [71] *Baeckea frutescens* leaves [70]	Inhibition activity toward copper-induced LDL oxidation with IC_50_ value of 3.98 ± 0.24 µM [70]
**86**	8-C-*β*-d-(6′-*O*-galloyl)glucosylnoreugenin [2-Methyl-5,7-dihydroxy-chromone-8-*β*-d-(6′-*O*-galloyl)-glucopyranoside]	*Syzygium aromaticum* L. leaves [91] *Syzygium aromaticum* L. flower buds [68,73]	Cytotoxicity against human ovarian cancer cells (A2780) with an IC_50_ value of 87.50 ± 1.56 µM [68] Significant inhibition capacity against Prolyl Endopeptidase with IC_50_ value of 1.48 ± 0.02 µM [73]
**87**	8-*β*-C-(2′-galloylglucopyranosyl)-5,7-dihydroxy-2-isopropylchromone	*Baeckea frutescens* leaves [70]	Active against copper-induced LDL oxidation with an IC_50_ value of 3.91 ± 0.18 µM [70]
**88**	Kunzeachromone F [2-Methyl-5,7-dihydroxy-chromone-8-*β*-d-(2’,3′-di-*O*-galloyl)-glucopyranoside]	*Kunzea ambigua* leaves [71]	No reported biological activity
**89**	Kunzeachromone B [2-Isopropyl-5,7-dihydroxy-chromone-8-*β*-d-(2’,3′-di-*O*-galloyl)-glucopyranoside]	*Kunzea ambigua* leaves [71]	No reported biological activity
**90**	2,5-dimethyl-8-C-*β*-d-glucopyranosyl-7-hydroxy-chromone	*Aloe vera* [92]	No reported biological activity
**91**	2-(*E*)-propenyl-7- methoxy-8-C-*β*-d-glucopyranosyl-5-methylchromone	*Aloe vera* [76,80]	BACE1 (*β*-secretase) inhibitory activity with an IC_50_ value of 20.5 µM [80]
**92**	8-C-*β*-d-glucosyl-(*R*)-aloesol	*Aloe vera* [76,80]	BACE1 (*β*-secretase) inhibitory activity (39.2%) at 100 μM [80]
**93**	8-C-*β*-d-glucosyl-7-*O*-methyl-(*R*)-aloesol	*Aloe vera* [76,80] and anerobic incubation of aloesin with bacterial mixture [93]	BACE1 (*β*-secretase) inhibitory activity (26.8%) at 100 μM [80]
**94**	8-C-*β*-d-glucosyl-(*S*)-aloesol	*Aloe vera* [76] and anerobic incubation of aloesin with bacterial mixture [93]	No reported biological activity
**95**	8-C-*β*-d-glucosyl-7-*O*-methyl-(*S*)-aloesol	*Aloe vera* [76] and anerobic incubation of aloesin with bacterial mixture [93]	No reported biological activity
**96**	8-C-*β*-d-glucosyl-7-*O*-methylaloediol	*Aloe vera* [76,80]	No reported biological activity
**97**	Neoaloesin A	*Aloe vera* [76] *Aloe barbadensis* leaves [94]	No reported biological activity
**98**	Aloesin	*Aloe vera* [76,80] *Aloe barbadensis* leaves [95]	Antioxidant activity (50 ± 1 μM trolox equivalent) at 100 mg of soluble solid/L solution [95] BACE1 inhibitory activity (37.5%) at 100 μM [80] Suppresses hyperpigmentation (40%) at 100 mg⁄g polyethylene glycol [2]
**99**	7-*O*-methylaloesin	*Aloe rupestris* leaves exudate [96]	No reported biological activity
**100**	Aloesin-2″,3″,4″,6″-tetra-*O*-acetate	Anerobic incubation of aloesin with bacterial mixture [93]	No reported biological activity
**101**	2″-*O*-tigloylaloesin	*Aloe cremnophila* leaves exudate [97]	No reported biological activity
**102**	8-*C*-*β*-d-glucopyranosyl-7-hydroxy-5-methylchromone-2-carboxylic acid	Herbal tea “*muti*” [98]	No reported biological activity
**103**	8-[C-*β*-d-[2-*O*-(*E*)-cinnamoyl]glucopyranosyl]-2-[(*R*)-2-hydroxypropyl]-7-methoxy-5-methylchromone	*Aloe vera* [76] *Aloe barbadensis* leaves [99]	Topical anti-inflammatory activity at 200 µg/ear [99]
**104**	Aloeresin D	*Aloe vera* [76]	No reported biological activity
**105**	Rabaichromone	*Aloe vera* [76]	No reported biological activity
**106**	Allo-aloeresin D	*Aloe vera* [76]	No reported biological activity
**107**	Aloeresin K	*Aloe vera* [76] *Aloe barbadensis* leaf skin [100]	No reported biological activity
**108**	Aloeresin J	*Aloe vera* [76] *Aloe barbadensis* leaf skin [100]	No reported biological activity
**109**	Aloeresin E	*Aloe vera* leaves [76,82]	Inhibition of tyrosinase enzyme (40% and 80% at 50 and 100 ppm, respectively) [82]
**110**	Isoaloeresin D	*Aloe vera* leaves [76,82]	Inhibition of tyrosinase enzyme (20% and 40% at 50 and 100 ppm, respectively) [82] Antiviral activity against Pepper mild mottle virus; PMMoV (37.5 ± 6.5% at 1.5 mg/mL) [81]
**111**	2′-*O*-[*p*-methoxy-(*E*)-cinnamoyl]-(*S*)-aloesinol	*Aloe nobilis* leaves [12]	BACE1 inhibitory activity (34.1%) at 100 μM [80]
**112**	Iso-rabaichromone	*Aloe vera* [76,82]	No reported biological activity
**113**	8-C-glucosyl-(2′-*O*-cinnamoyl)-7-*O*-methylaloediol B	*Aloe vera* leaves [76]	No reported biological activity
**114**	Aloeresin A	*Aloe vera* [76]	Antioxidant activity [101] α-glucosidase inhibitory activities, with IC_50_ values of 11.94 and 2.16 mM against rat intestinal sucrase and maltase [79]
**115**	7-*O*-methyl-aloeresin A	*Aloe vera* [76]	Tyrosinase inhibitory activity with an IC_50_ value of 9.8 μM [81]
**116**	6′-*O*-coumaroyl-aloesin	*Aloe vera* [76]	Anti-lipid peroxidation activity with an IC_50_ value of 476.4 ± 0.9 µM [75]
**117**	7-Methoxy-6′-*O*-coumaroyl-aloesin	*Aloe vera* [76]	Weak anticancer activity against breast cancer cell line, MDA-MB-231 (induce 30% decline in cell survival at 25 μM ) [102]
**118**	2′-Feruloylaloesin	*Aloe nobilis* leaves [80]	Inhibition activity against *β*-secretase (36.4%) at 100 μM [80] Inhibition effect against mushroom tyrosinase (27 ± 0.57%) at 0.4 μM [103]
**119**	2′-Feruloyl-7-*O*-methylaloesin	*Aloe nobilis* leaves [80]	Inhibition activity against BACE1 (*β*-secretase) (48.7%) at 100 μM [80]
**120**	9-Dihydroxyl-2′-*O*-(*Z*)-cinnamoyl-7-methoxy-aloesin	*Aloe vera* [76]	Inhibition of tyrosinase enzyme (9.5 ± 9.0%) at 100 μM Antiviral against Pepper mild mottle virus; PMMoV (31.5 ± 4.2% inhibition at 1.5 mg/mL) [81]
**121**	4′-*O*-*β*-d-glucosyl-isoaloeresin DI	*Aloe vera* [76]	No reported biological activity
**122**	4′-*O*-*β*-d-glucosyl-isoaloeresin DII	*Aloe vera* [76]	No reported biological activity

**Table 11:** Prenyl and isoprenyl chromone glycosides with their sources and biological activities.

No.	Compound	Source	Biological Activity
**123**	Cnidimoside A	*Cnidium Juponicum* whole plant [104,105] *Cnidium monnieri* fruits [60,106]	Significant inhibition of fat accumulation at 300 μM in differentiated adipocytes [60] Antitumor and antimetastatic actions at 0.1–100 μM (*in vitro*) and 20, 50 mg/kg, twice daily (in vivo) [105]
**124**	Cnidimoside B	*Cnidium Juponicum* whole plant [104] *Cnidium monnieri* fruits [60]	Significant ihibition of fat accumulation at 300 μM in differentiated adipocytes [60]
**125**	2-methyl-5-hydroxy-6-(2- butenyl-3-hydroxymethyl)-7-(*β*-d-glucopyranosyloxy)-4H- 1-benzopyran-4-one	*Cnidium monnieri* fruits [60] *Angelica archangelica* [107] *Archangelica litoralis* [107]	No reported biological activity
**126**	Hydroxycnidimoside A	*Cnidium monnieri* fruits [60,106]	Significant inhibition of fat accumulation at 300 μM in differentiated adipocytes [60]
**127**	Monnieriside B	*Cnidium monnieri* fruits [60,106]	
**128**	Monnieriside C	*Cnidium monnieri* fruits [60]	No reported biological activity
**129**	Monnieriside D		
**130**	Monnieriside E		
**131**	7,8-Secoeranthin-*β*-d-glucopyranoside (8-{(2*E*)-4-[*β*-d-glucopyranosyl)oxy]-3-methylbut-2-enyl}-5,7- dihydroxy-2-methyl-4H-l-benzopyran-4-one)	*Eranthis hyemalis* tubers [108]	No reported biological activity
**132**	2-C-Hydroxy-7,8-seroeranthipn-*β*-d-glucopyranoside (8-{(2*E*)-4-[*β*-d-glucopyranosyl)oxy]-3-methylbut-2-enyl}-5,7- dihydroxy-2-(hydroxymethyl)-4H-1-benzopyran-4-on2)		
**133**	7-[(*β*-d-glucopyranosyl)oxy]-5-hydroxy-8-[(2*E*)-4-hydroxy-3-methylbut-2-enyl]-2-methyl-4H-1-benzopyran-4-one	*Eranthis cilicica* tubers [109]	No reported biological activity
**134**	7-[(*β*-d-glucopyranosyl)oxy]-5-hydroxy-2-hydroxymethyl-8- [(2*E*)-4-hydroxy-3-methylbut-2-enyl]-4H-1-benzopyran-4-one		

**Table 12:** Phenyl ethyl chromone glycosides with their sources.

No.	Compound	Source
**135**	Ononin glucoside	*Ononis vaginalis* whole plant [110]
**136**	7-Glucosyloxy-5-hydroxy-2-[2-(4-hydroxyphenyl)ethyl]chromone	*Cucumis melo* seeds [111]
**137**	Aquilarinoside C (6,4′-dimethoxy-3′-hydroxy-2- (2-phenylethyl)chromone 7-*O*-*β*-d-glucopyranoside)	*Aquilaria sinensis* stems [112]
**138**	2-(2-phenylethyl) chromone-8-*O*-*β*-d-glucopyranoside	*Imperata cylindrical* rhizomes [113]
**139**	2-[2-(4-glucosyloxy-3-methoxyphenyl)ethyl]chromone	*Aquilaria sinensis* resinous heartwood [114]

**Table 13:** Furano-chromone glycosides with their sources and biological activities.

No.	Compound	Source	Biological Activity
**140**	Khellol glucoside (Khellinin; Khelloside)	*Ammi visnaga* fruits [117] *Eranthhis hyernalis* tubers [108]	Potent coronary vasodilator and bronchodilator [115] Hypocholesterolemic effect at 20 mg/kg per day [116]
**141**	Norkhelloside	*Cimicifuga heracleifolia* rhizomes [118]	No reported biological activity
**142**	7-[(*O*-*β*-d-glucopyranosyl-(1→6)- *β*-d-glucopyranosyl)oxy]methyl-4-hydroxy-5*H*-furo[3,2-g][1]benzopyran-5-one	*Eranthis cilicica* tubers [109]	No reported biological activity
**143**	4′-*O*-*β*-d-glucopyranosylvisamminol (Monnieriside G)	*Cnidium monnieri* fruits [60] *Saposhnikovia divaricata* roots [119]	Antitumor activity against SK-OV-3 with an IC_50_ value of 93.91 μM [120]
**144**	4′-*O*-*β*-d-glucopyranosyl-5-*O*-methylvisamminol	*Ledebouriella seseloides* roots and rhizomes [121] *Saposhnikovia divaricata* roots [119,122] *Diplolophium buchananii* aerial parts [123] *Sphallerocarpus gracilis* roots [124]	Analgesic, antipyretic, anti-inflammatory, and anti-platelet aggregation activities [125,126] Antitumor activity with against H-460 cell line with an IC_50_ value of 86.91 μM [120]
**145**	(2’*S*)-4′-*O*-*β*-d-apiofuranosyl- (1→6)-*β*-d-glucopyranosylvisamminol	*Saposhnikovia divaricata* roots [119]	Antitumor activities against PC-3 and SK-OV-3 cell lines with IC_50_ values of 48.5, 81.91 μM, respectively [120]
**146**	*prim*-*O*-glucosylcimifugin	*Ammi visnaga* fruits [127] *Angelica genuflexa* roots [128] *Eranthis hyernalis* tubers [108] *Angelica japonica* roots [129] *Cimicifuga foetida* rhizomes [130] *Diplolophium buchananii* aerial parts [123] *Saposhnikovia divaricata* roots [131]	Analgesic, antipyretic, anti-inflammatory, anti-platelet aggregation and antitumor activities [125,126,131]
**147**	Cimifugin-4′-*O*-[6″-feruloyl]-*β*-d-glucopyranoside	*Cimicifuga foetida* rhizomes [132]	No reported biological activity
**148**	Monnieriside F	*Cnidium monnieri* fruits [60]	No reported biological activity

**Table 14:** Pyrano-chromone glycosides with their sources and biological activities.

No.	Compound	Source	Biological Activity
**149**	3′-*O*-glucopyranosylhamaudol (*Sec*-*O*-glucopyranosylhamaudol)	*Angelica genuflexa* roots [128] *Angelica japonica* roots [129] *Glehnia littoralis* roots [133] *Peucedanum japonicum* roots [134] *Saposhnikovia divaricata* roots [119,122]	Antitumor activity against H-460 cell line with an IC_50_ value of 94.25 ± 1.45 μM [119]
**150**	(3’*S*)-3′-*O*-*β*-d-apiofuranosyl-(1→6)- *β*-d-glucopyranosylhamaudol	*Saposhnikovia divaricata* roots [119]	Antitumor activity against SK-OV-3 with an IC_50_ value of 86.21 ± 1.03 μM [119]
**151**	(2’S)-2′-hydroxy-7-*O*-methylallopeucenin 2′-*O*-*β*-d-glucopyranoside	*Diplolophium buchananii* aerial parts [123]	No reported biological activity

**Table 15:** Oxepino-chromone glycosides with their sources.

No.	Compound	Source
**152**	Eranthin *β*-d-glucopyranoside	*Eranthis hyemalis* tubers [108]
**153**	Eranthin *β*-d-gentiobioside	*Eranthis cilicica* tubers [109] *Eranthis hyemalis* tubers [108]
**154**	2-C-Hydroxyeranthin *β*-d-glucopyranoside	*Eranthis hyemalis* tubers [108]
**155**	9-[(*O*-*β*-d-glucopyranosyl-(1→6)-*β*-d-glucopyranosyl)oxy]methyl-8,11-dihydro-5,9-dihydroxy-2-methyl-4*H*pyrano[2,3-g][1]benzoxepin-4-one	*Eranthis cilicica* tubers [109]

**Table 16:** Pyrido-chromone glycosides with its source and biological activity.

No.	Compound	Source	Biological Activity
**156**	Schumanniofoside	*Schumanniophyton magnificum* stem bark [135]	Anti-snake venom activity at 0.01–0.16 g/kg [135]

**Table 17:** Hybrids of furanochromones with cycloartane triterpenes with their sources and biological activities.

No.	Compound	Source	Biological Activity
**157**	Cimitriteromone A	*Cimicifuga foetida* rhizomes [130]	No reported biological activity
**158**	Cimitriteromone B	*Cimicifuga foetida* rhizomes [130]	Anti-proliferative activity with an IC_50_ value of 15.73 ± 0.59 μM [130]
**159**	Cimitriteromone C	*Cimicifuga foetida* rhizomes [130]	No reported biological activity
**160**	Cimitriteromone D	*Cimicifuga foetida* rhizomes [130]	Anti-proliferative activity with an IC_50_ value of 24.21 ± 0.61 μM [130]
**161**	Cimitriteromone E	*Cimicifuga foetida* rhizomes [130]	No reported biological activity
**162**	Cimitriteromone F	*Cimicifuga foetida* rhizomes [130]	No reported biological activity
**163**	Cimitriteromone G	*Cimicifuga foetida* rhizomes [130]	No reported biological activity
**164**	Cimitriteromone H	*Cimicifuga foetida* rhizomes [136]	No reported biological activity
**165**	Cimitriteromone I	*Cimicifuga foetida* rhizomes [136]	Anti-proliferative activity with an IC_50_ value of 27.14 ± 1.38 μM [136]

**Table 18:** Chromone alkaloids aminoglycosides with their sources and biological activities.

No.	Compound	Source	Biological Activity
**168**	Kidamycin (rubiflavin B)	*Streptomyces phaeoverticillatus* var. *takatsukiensis* [138]	Antibiotic with MIC (minimum inhibitory concentration) ranges from 0.19–1.56 μg/mL and potent antitumor activity [138]
**169**	Neopluramycin	*Streptomyces pluricolorescens* [139,140]	Antibiotics and potent antitumor activity [140]
**170**	14, 16-Epoxykidamycin	*Streptomyces pluricolorescens* [141]	Antibiotic against Gram-positive bacteria with MIC ranges from 0.5 to 10 μg/mL and antitumor activity [141]
**171**	Pluramycin A	*Streptomyces pluricolorescens* [139,140]	Antibiotic and antitumor activity [140]
**172**	Rubiflavin A	*Streptomyces* species [142]	Antibiotic and antitumor activity [142]
**173**	Rubiflavin C-1		
**174**	Rubiflavin C-2		
**175**	Rubiflavin D		
**176**	Rubiflavin E		
**177**	Rubiflavin F (isokidamycin)		
**178**	PD 121,222	*Streptomyces* species [143]	Antibiotic and potent antitumor activity [143]
**179**	Hedamycin	*Streptomyces griseoruber* [144,145]	Antibiotic and potent antitumor activity against HeLa cells [144]
**180**	Ankinomycin (deangolosaminylhedamycin)	*Streptomyces* species [146]	Antibiotic against Gram-positive bacteria with MICs ranges from 0.39–1.56 μg/mL and potent antitumor activity [146]
**181**	Pluraflavin A	*Saccharothrix* species [147]	Antibiotic and potent antitumor activity [147]
**182**	Pluraflavin B		
**183**	Pluraflavin E		
**184**	Altromycin A	*Actinomycete*, AB 1246E-26 [148,149]	Antibiotic against Gram-positive bacteria and potent antitumor activity [137]
**185**	Altromycin B		
**186**	Altromycin C		
**187**	Altromycin D		
**188**	Altromycin E	*Actinomycete*, AB 1246E-26 [137]	
**189**	Altromycin F		
**190**	Altromycin G		
**191**	Altromycin H		
**192**	Altromycin I		

**Table 19:** UV Band maxima of chromones and chromanone in 3-methylpentane at 25 °C.

Chromones		Chromanones
Band system	λ_max_	λ_max_
A	360, 352, 345, 337, 324	363, 347 322(sh), 312 252, 246 219(sh), 213
B	301, 290, 283	
C	225, 246, 239, 227, 223, 216, 202	

**Table 20:** The ^13^C-NMR spectral data of compounds **1**–**14** except those which have no reported ^13^C-NMR data.

C	1	2	3	4	5	7	11	13	14
**2**	161.2	147.4	147.7	147.8	147.4	167.2	167.8	158.6	159.5
**3**	93.1	141.2	141.0	138.1	141.5	111.7	109.1	112.0	112.8
**4**	166.6	178.4	178.5	177.0	178.4	180.3	183.2	183.6	184.4
**5**	137.1	163.4	163.3	161.5	163.4	160.2	93.9	163.4	163.9
**6**	132.0	100.1	100.2	98.8	100.2	104.6	162.0	101.3	101.9
**7**	127.8	166.4	166.3	164.3	166.5	164.7	110.2	164.9	165.2
**8**	114.9	95.0	95.0	93.8	95.0	99.1	159.6	96.2	96.9
**9**	154.3	159.3	159.3	157.2	159.4	161.1	156.6	159.5	160.2
**10**	114.5	106.2	106.2	104.8	106.1	109.2	106.1	108.4	109.3
**11**	23.2	-	-	-	-	19.9	20.3	-	-
**12**	-	-	-	-	-	-	8.2	-	-
**1′**	100.1	104.6	104.4	100.6	104.2	105.0	101.7	101.6	102.1
**2′**	73.2	74.6	72.0	69.9	74.8	74.6	75.1	74.7	75.1
**3′**	77.5	77.1	73.6	70.2	77.4	77.3	78.4	77.9	77.8
**4′**	69.6	70.8	69.2	71.5	71.3	71.3	80.5	71.2	73.9
**5′**	76.7	67.1	67.1	69.7	78.5	78.7	78.1	78.4	77.5
**6′**	60.7	-	-	17.7	62.6	62.5	60.9	62.4	171.5
**OCH_3_**	-	-	-	-	-	-	-	-	53.8
**Solvent**	DMSO-*d*_6_	CD_3_OD	CD_3_OD	DMSO-*d*_6_	CD_3_OD	CD_3_OD	C_5_D_5_N	CD_3_OD	CD_3_OD
**References**	[7]	[10]	[12]	[9]	[11]	[17]	[1]	[27]	[28]

**Table 21:** The ^13^C-NMR spectral data of compounds **15**–**23** except which has no reported ^13^C-NMR data.

C	15	16	17	18	20	21	22	23
**2**	158.1	158.4	158.5	168.9	161.6	174.3	175.5	174.6
**3**	110.8	111.9	112.0	108.8	108.8	107.8	106.2	106.6
**4**	181.8	183.6	183.6	182.5	182.5	184.3	183.3	183.1
**5**	161.3	163.1	163.2	161.2	157.9	163.0	162.2	162.7
**6**	99.8	101.2	101.2	99.8	100.3	101.0	100.6	100.7
**7**	162.9	164.6	164.8	163.1	168.9	164.8	164.2	164.2
**8**	94.6	96.2	96.2	94.9	95.0	95.9	95.3	95.3
**9**	157.7	159.4	159.4	157.9	163.4	159.5	158.4	158.4
**10**	106.8	108.5	108.5	105.5	105.5	107.0	106.5	107.4
**11**	-	-	-	20.5	20.5	28.3	33.3	40.4
**12**	-	-	-	-	-	11.2	19.9	27.6
**13**	-	-	-	-	-	-	19.9	17.7
**14**	-	-	-	-	-	-	-	11.7
**1′**	99.7	101.5	101.5	100.6	99.9	101.6	101.7	101.7
**2′**	73.1	74.7	74.7	73.3	73.5	74.7	74.8	74.8
**3′**	76.2	77.9	77.9	76.6	77.6	77.8	78.5	78.5
**4′**	69.7	71.9	72.0	69.6	70.7	71.2	71.2	71.1
**5′**	74.1	75.8	76.0	66.2	76.8	78.4	79.3	79.2
**6′**	63.4	64.2	64.7	-	61.1	62.4	62.4	62.3
**1″**	119.5	127.4	127.3	-	-	-	-	-
**2″**	108.8	133.7	131.2	-	-	-	-	-
**3″**	145.6	115.8	117.0	-	-	-	-	-
**4″**	138.6	160.0	161.4	-	-	-	-	-
**5″**	145.6	115.8	117.0	-	-	-	-	-
**6″**	108.8	133.7	131.2	-	-	-	-	-
**7″**	165.9	145.1	146.9	-	-	-	-	-
**8″**	-	116.0	115.1	-	-	-	-	-
**9″**	-	168.1	168.9	-	-	-	-	-
**Solvent**	DMSO-*d_6_*	CD_3_OD	CD_3_OD	DMSO-*d_6_*	DMSO-*d_6_*	CD_3_OD	C_5_D_5_N	C_5_D_5_N
**References**	[29]	[30]	[30]	[31]	[157]	[40]	[41]	[41]

**Table 22:** The ^13^C-NMR spectral data of compounds **24**–**32** except which has no reported ^13^C-NMR data.

C	24	25	26	28	29	30	31	32
**2**	168.7	157.3	167.9	168.2	168.9	169.2	168.9	168.5
**3**	108.7	108.3	108.8	108.8	108.8	109.3	108.8	108.4
**4**	182.4	181.9	182.7	182.8	182.5	184.2	182.5	182.1
**5**	161.5	161.1	162.4	162.4	161.7	163.1	161.7	161.3
**6**	99.9 *	108.7	100.6	100.7	100.2	100.8	100.0	99.6
**7**	162.9	162.6	163.9	163.9	161.9	163.4	161.9	161.6
**8**	94.9	108.2	95.3	95.1	95.2	95.7	95.1	94.7
**9**	157.7	168.2	158.3	158.3	157.9	159.5	157.9	157.5
**10**	105.5	105.0	106.3	106.3	105.7	106.8	105.6	105.2
**11**	20.3	19.9	19.9	20.1	20.5	20.4	20.5	20.1
**1′**	99.8 *	99.3	101.9	102.0	98.8	99.6	98.5	98.1
**2′**	73.3	77.0	74.6	74.6	81.2	71.0	69.8	69.5
**3′**	76.5	75.8	78.5	78.5	70.4	82.5	82.1	70.3
**4′**	70.2	68.9	71.5	71.6	70.9	72.4	70.7	81.4
**5′**	74.1	76.0	77.4	77.5	69.4	70.9	68.7	68.5
**6′**	63.7	60.5	69.0	68.0	18.3	18.1	18.0	17.9
**1″**	-	108.7	111.2	102.7	105.0	105.9	104.8	104.4
**2″**	-	76.7	77.9	72.0	74.5	75.4	74.1	74.5
**3″**	-	79.2	80.3	72.8	77.2	77.8	76.8	77.1
**4″**	-	73.9	75.1	74.1	70.0	71.1	70.6	70.1
**5″**	-	64.1	65.8	69.8	76.7	77.7	74.8	76.7
**6″**	-	-	-	18.6	61.5	62.2	64.1	61.2
**CH_3_CO**	20.9, 170.5	-	-	-	-	-	21.1, 170.6	-
**Solvent**	DMSO-*d_6_*	CDCl_3_, DMSO-*d_6_*	C_5_D_5_N	C_5_D_5_N	DMSO-*d_6_*	DMSO-*d_6_*	DMSO-*d_6_*	DMSO-*d_6_*
**References**	[33]	[16]	[44]	[46]	[31]	[22]	[31]	[47]

* Data interchangeable.

**Table 23:** The ^13^C-NMR spectral data of compounds **33**–**41**.

C	33	34	35	36	37	38	39	40	41
**2**	168.3	168.2	168.3	168.3	157.8	154.8	154.7	168.6	168.4
**3**	108.2	108.7	108.3	108.2	110.6	120.3	125.8	108.7	108.4
**4**	181.4	182.7	181.9	181.9	181.5	183.8	183.5	182.5	182.3
**5**	161.1	162.3	161.1	161.2	157.7	163.0	163.1	158.4	185.1
**6**	99.5	100.6	99.8	99.5	108.9	100.9	100.8	109.1	108.5
**7**	161.4	163.7	162.6	162.6	160.9	164.6	164.6	161.1	155.56
**8**	94.5	94.6	94.3	94.5	93.3	95.8	95.8	93.3	97.7 *
**9**	157.3	158.3	157.4	157.4	155.4	159.4	159.5	155.9	160.3
**10**	105.0	106.3	105.1	105.1	106.0	107.4	107.6	105.1	105.0
**11**	19.9	19.9	19.8	19.8	7.4	10.2	19.3	20.5	19.9
**12**	-	-	-	-	-	-	13.4	7.9	6.9
**1′**	98.0	101.6	99.5	98.1	100.1	101.6	101.6	100.2	93.1 *
**2′**	69.5	74.4	72.9	73.0	73.1	74.7	74.7	73.8	77.3
**3′**	69.8	78.1	76.1	76.2	76.4	77.8	77.8	76.5	74.5
**4′**	80.9	71.3	69.9	69.9	69.6	71.2	71.2	79.4	69.7
**5′**	68.4	75.8	73.9	73.9	77.1	78.4	78.4	76.1	73.3
**6′**	17.9	64.5	63.4	63.4	60.6	62.4	62.4	60.7	60.5
**1″**	102.3	121.0	128.5	127.9	-	-	-	-	-
**2″**	81.4	110.4	114.8	114.9	-	-	-	-	-
**3″**	76.6	147.4	146.9	148.9	-	-	-	-	-
**4″**	69.8	140.8	147.7	149.3	-	-	-	-	-
**5″**	77.6	147.4	120.7	122.5	-	-	-	-	-
**6″**	61.0	110.4	115.9	115.8	-	-	-	-	-
**7″**	-	167.1	144.6	144.7	-	-	-	-	-
**8″**	-	-	115.7	115.8	-	-	-	-	-
**9″**	-	-	166.1	166.2	-	-	-	-	-
**1‴**	103.5	-	99.5	99.5	-	-	-	-	-
**2‴**	71.2	-	70.3	71.2	-	-	-	-	-
**3‴**	71.7	-	71.0	71.6	-	-	-	-	-
**4‴**	66.4	-	67.1	67.1	-	-	-	-	-
**5‴**	64.2	-	75.0	74.8	-	-	-	-	-
**6‴**	-	-	61.0	61.0	-	-	-	-	-
**OCH_3_**	-	-	-	55.8	-	-	-	60.1	-
**CH_3_CO**	-	-	-	-	-	-	-	-	20.7, 169.6
**Solvent**	DMSO-*d_6_*	C_5_D_5_N	DMSO-*d_6_*	DMSO-*d_6_*	DMSO-*d_6_*	CD_3_OD	CD_3_OD	DMSO-*d_6_*	DMSO-*d_6_*
**References**	[47]	[48]	[33]	[33]	[49]	[50]	[50]	[23]	[51]

* Data interchangeable.

**Table 24:** The ^13^C-NMR spectral data of compounds **43**–**52** except those which have no reported ^13^C-NMR data.

C	43	45	46	47	48	49	50	52
**2**	168.7	168.4	168.4	169.5	170.0	160.0	164.9	164.9
**3**	108.4	108.0	108.0	108.4	109.1	112.5	101.9	118.6
**4**	182.9	182.7	182.7	182.4	185.0	185.0	178.8	177.6
**5**	159.5	152.6	155.9	152.5	157.8	158.8	141.8	137.9
**6**	98.3	109.7	114.3	135.2	116.3	117.3	116.6	110.5
**7**	160.9	158.5	158.8	157.8	160.2	161.2	160.4	159.8
**8**	104.6	114.3	109.8	94.7	111.6	112.9	111.5	99.6
**9**	155.0	155.9	152.6	155.6	154.6	155.5	159.3	158.5
**10**	105.2	106.5	106.5	106.2	108.2	110.6	117.1	115.7
**11**	20.5	20.0	20.1	20.2	20.5	10.3	22.8	19.4
**12**	8.1	8.8	9.0	-	8.9	10.5	19.9	48.7
**13**	-	8.9	9.1	-	9.5	-	-	205.3
**14**	-	-	-	-	-	-	-	52.0
**15**	-	-	-	-	-	-	-	62.7
**16**	-	-	-	-	-	-	-	23.6
**1′**	100.3	104.3	104.4	100.8	105.7	106.5	100.3	102.1
**2′**	73.9	76.0	74.1	74.1	75.3	76.5	77.6	73.0
**3′**	76.5	73.9	76.3	78.0	75.6	78.5	73.6	76.3
**4′**	79.4	70.0	69.9	69.9	71.7	72.5	70.1	69.4
**5′**	76.1	73.4	77.0	77.0	77.7	76.4	76.9	77.0
**6′**	60.7	63.0	61.0	61.2	64.3	65.3	61.0	60.5
**1″**	-	-	-	-	-	173.1	-	-
**2″**	-	-	-	-	-	47.3	-	-
**3″**	-	-	-	-	-	71.4	-	-
**4″**	-	-	-	-	-	46.9	-	-
**5″**	-	-	-	-	-	176.6	-	-
**6″**	-	-	-	-	-	28.4	-	-
**OCH_3_**	60.1	-	-	56.7	-	-	-	-
**CH_3_CO**	-	20.4, 170.1	-	-	20.4, 172.5	-	-	-
**Solvent**	DMSO-*d_6_*	DMSO-*d_6_*	*	DMSO-*d_6_*	*	CD_3_OD	CDCl_3_	DMSO-*d_6_*
**References**	[23]	[51]	[24]	[36]	[24]	[52]	[53]	[57]

* The authors did not report the NMR solvent.

**Table 25:** The ^13^C-NMR spectral data of compounds **53**–**64** except those which have no reported ^13^C-NMR data.

C	53	54	55	56	57	60	61	62	64
**2**	161.0	166.3	169.9	166.6	166.5	105.9	104.7	83.5	148.0
**3**	117.2	110.7	109.3	107.6	106.9	46.2	52.9	42.2	140.5
**4**	178.1	177.4	184.0	182.2	182.5	199.2	198.9	193.7	178.9
**5**	141.3	117.8	96.0	162.0	162.0	165.3	165.2	161.5	163.6
**6**	113.0	125.0	162.9	99.5	98.8	97.5	97.5	100.0	100.2
**7**	159.9	119.2	101.0	165.1	164.8	168.4	168.4	166.6	166.2
**8**	99.7	147.4	164.7	94.5	93.6	97.0	97.0	99.1	95.3
**9**	158.8	147.3	159.3	158.1	158.2	160.4	160.2	166.2	159.3
**10**	116.1	125.4	119.0	104.3	104.2	102.0	102.4	106.7	106.6
**11**	47.3	20.1	20.3	66.1	66.0	13.8	23.7	33.2	-
**12**	202.5	-	-	-	-	-	11.8	18.2 *	-
**13**	29.8	-	-	-	-	-	-	-	-
**14**	22.3	-	-	-	-	-	-	-	-
**1’**	101.3	102.4	101.6	102.8	102.7	104.1	104.1	104.0	102.1
**2’**	73.1	74.8	74.7	73.9	73.6	75.0	75.0	74.6	72.0
**3’**	76.3	78.6	78.3	76.4	76.6	77.9	77.9	77.4	74.0
**4’**	69.5	71.2	71.4	67.7	70.1	71.0	71.0	71.2	71.5
**5’**	77.0	79.2	77.8	63.6	76.8	78.0	78.0	78.5	72.4
**6’**	60.9	62.4	62.4	-	61.3	62.5	62.5	62.5	62.5
**Solvent**	DMSO-*d_6_*	C_5_D_5_N	CD_3_OD	DMSO-*d_6_*	CD_3_OD	CD_3_OD	CD_3_OD	CD_3_OD	CD_3_OD
**References**	[57]	[58]	[59]	[31]	[60]	[54]	[50]	[63]	[65]

* Data interchangeable.

**Table 26:** The ^13^C-NMR spectral data of compounds **66**–**72**.

C	66	67	68	69	70	71	72
**2**	167.3	174.7	168.3	168.4	167.8	169.4	174.4
**3**	107.8	105.1	108.6	108.8	107.0	109.2	105.3
**4**	181.8	182.2	181.8	181.8	182.0	184.3	182.2
**5**	160.6	160.6	160.6	160.6	160.4	162.3	160.4
**6**	108.7	108.7	108.6	108.8	108.1	108.9	107.0
**7**	163.2	163.3	163.1	163.1	162.4	165.0	163.4
**8**	93.3	93.4	93.3	93.3	93.9	95.2	93.7
**9**	156.6	156.7	156.6	156.6	157.0	159.4	156.9
**10**	103.0	103.3	103.2	103.2	102.9	105.1	103.1
**11**	19.8	32.3	43.1	66.2	20.0	20.4	32.4
**12**	-	19.8	64.1	46.0	-	-	19.8
**13**	-	19.8	23.3	63.8	-	-	19.8
**14**	-	-	-	43.8	-	-	-
**15**	-	-	-	23.5	-	-	-
**1′**	73.0	73.0	72.9	72.9	70.8	75.6	70.7
**2′**	70.1	70.2	70.0	70.0	72.0	72.6	72.0
**3′**	78.9	78.9	78.8	78.8	76.7	80.1	76.6
**4′**	70.6	70.6	70.5	70.5	70.8	71.9	70.7
**5′**	81.4	81.6	81.4	81.4	82.0	80.2	81.8
**6′**	61.4	61.5	61.4	61.4	61.6	65.2	61.5
**1″**	-	-	-	-	119.9	121.6	119.9
**2″**	-	-	-	-	108.9	110.4	108.7
**3″**	-	-	-	-	145.4	146.6	145.4
**4″**	-	-	-	-	138.2	140.0	138.1
**5″**	-	-	-	-	145.4	146.6	145.4
**6″**	-	-	-	-	108.9	110.4	108.9
**7″**	-	-	-	-	164.8	168.6	164.7
**Solvent**	DMSO-*d_6_*	DMSO-*d_6_*	DMSO-*d_6_*	DMSO-*d_6_*	DMSO-*d_6_*	CD_3_OD	DMSO-*d_6_*
**References**	[69]	[69]	[72]	[72]	[71]	[73]	[69]

**Table 27:** The ^13^C-NMR spectral data of compounds **73**–**79** except those which have no reported ^13^C-NMR data.

C	73	74	75	76	79
**2**	167.7	174.9	162.3	163.1	160.6
**3**	106.3	105.3	113.2	111.0	112.4
**4**	182.0	182.2	181.9	181.8	178.5
**5**	161.1	160.9	143.1	146.1	*
**6**	108.1	106.4	127.0	128.3	126.5
**7**	163.1	163.2	160.1	161.1	160.8
**8**	93.4	93.4	119.4	121.1	100.6
**9**	157.1	157.0	161.3	157.9	159.0
**10**	103.0	103.1	115.8	114.3	114.6
**11**	19.9	32.4	48.7	48.5	47.8
**12**	-	19.78	204.4	204.6	202.4
**13**	-	19.75	29.8	30.7	29.8
**14**	-	-	23.3	23.3	22.5
**CH_3_O**	-	-	-	55.8	-
**1′**	70.7	70.7	75.5	75.6	71.4
**2′**	69.7	69.7	71.9	72.3	81.7
**3′**	77.6	77.6	80.0	79.9	78.2
**4′**	68.7	68.7	72.9	72.1	70.1
**5′**	81.8	81.7	79.8	78.5	81.1
**6′**	61.2	61.2	65.2	65.4	60.9
**1″**	119.7	119.7	133.6	128.3	105.1
**2″**	108.9	108.9	131.2	131.0	74.3
**3″**	145.5	145.4	116.8	115.3	76.1
**4″**	138.5	138.4	161.3	160.4	69.3
**5″**	145.5	145.4	116.8	115.3	76.1
**6″**	108.9	108.9	131.2	131.0	60.3
**7″**	165.3	165.4	146.2	146.2	-
**8″**	-	-	114.9	116.0	-
**9″**	-	-	169.2	169.1	-
**1‴**	119.1	119.0	-	-	-
**2‴**	108.7	108.7	-	-	-
**3‴**	145.3	145.3	-	-	-
**4‴**	138.4	138.3	-	-	-
**5‴**	145.3	145.3	-	-	-
**6‴**	108.7	108.7	-	-	-
**7‴**	164.6	164.4	-	-	-
**Solvent**	DMSO-*d_6_*	DMSO-*d_6_*	CD_3_OD	CD_3_OD	DMSO-*d_6_*
**References**	[71]	[71]	[74]	[74]	[78]

* The authors missed assigning this position.

**Table 28:** The ^13^C-NMR spectral data of compounds **80**–**89**.

C	80	81	82	83	84	85	86	87	88	89
**2**	167.0	169.3	174.4	173.0	168.2	167.6	167.2	174.8	167.7	174.5
**3**	107.4	108.7	105.0	106.2	108.3	107.8	107.5	105.4	108.0	105.6
**4**	181.9	184.3	182.2	181.9	181.9	182.2	181.9	182.4	182.2	182.4
**5**	160.4	149.4	160.4	160.4	160.3	160.8	160.5	160.7	161.0	161.0
**6**	98.4	94.5	98.5	98.7	98.3	97.6	98.3	97.8	98.0	97.9
**7**	162.5	162.8	162.8	164.2	162.7	162.2	162.6	162.3	162.4	162.6
**8**	104.3	105.2	104.1	104.7	108.4	103.9	104.0	102.8	103.9	102.1
**9**	156.1	164.6	156.5	156.6	158.9	157.1	156.3	157.2	157.1	157.2
**10**	103.5	106.3	102.0	103.3	103.8	102.8	103.5	104.0	102.0	104.0
**11**	19.6	20.3	32.7	39.1	42.1	11.0	19.7	33.1	20.1	33.1
**12**	-	-	20.0	27.0	66.5	-	-	19.8	-	20.3
**13**	-	-	19.5	11.4	45.8	-	-	20.3	-	20.1
**14**	-	-	-	17.4	64.1	-	-	-	-	-
**15**	-	-	-	-	23.6	-	-	-	-	-
**1′**	73.1	74.9	73.1	73.3	73.2	70.6	73.3	70.7	70.7	70.8
**2′**	71.0	72.8	71.2	71.2	70.3	72.5	70.0	72.5	70.1	70.2
**3′**	78.5	80.0	78.5	78.7	78.6	76.1	78.1	76.0	77.0	77.0
**4′**	70.3	71.7	70.8	70.9	71.0	70.8	70.7	71.2	68.5	68.9
**5′**	81.1	82.4	81.5	81.5	81.4	81.7	78.3	82.0	81.5	81.9
**6′**	61.3	62.9	61.8	61.7	61.5	61.5	63.8	61.8	61.0	61.3
**1″**	-	-	-	-	-	119.7	119.4	119.6	119.6	119.6
**2″** **, 6″**	-	-	-	-	-	108.8	108.5	108.7	108.9	108.9
**3″** **, 5″**	-	-	-	-	-	145.5	145.5	145.4	145.6	145.5
**4″**	-	-	-	-	-	138.8	138.3	138.3	138.7	138.7
**7″**	-	-	-	-	-	165.0	165.8	165.0	165.5	165.4
**1‴**	-	-	-	-	-	-	-	-	118.7	118.7
**2‴** **, 6‴**	-	-	-	-	-	-	-	-	108.7	108.7
**3‴** **, 5‴**	-	-	-	-	-	-	-	-	145.5	145.4
**4‴**	-	-	-	-	-	-	-	-	138.5	138.6
**7‴**	-	-	-	-	-	-	-	-	164.8	164.8
**OCH_3_**	-	56.8	-	-	-	-	-	-	-	-
**Solvent**	DMSO-*d*_6_	CD_3_OD	DMSO-*d*_6_	DMSO-*d*_6_	DMSO-*d*_6_	DMSO-*d*_6_	DMSO-*d*_6_	DMSO-*d*_6_	DMSO-*d*_6_	DMSO-*d*_6_
**References**	[86]	[87]	[158]	[158]	[72]	[71]	[91]	[69]	[71]	[71]

**Table 29:** The ^13^C-NMR spectral data of compounds **91**–**100** except which has no reported ^13^C-NMR data.

C	91	93	94	95	96	97	98	99	100
**2**	162.7	164.9	167.1	167.0	169.2	160.2	160.2	160.8	159.6
**3**	109.8	111.2	112.2	111.9	110.8	112.6	112.4	113.0	113.7
**4**	182.7	178.7	182.3	181.9	182.4	178.3	178.5	178.9	179.1
**5**	144.0	141.0	143.1	143.7	144.1	139.5	140.1	141.3	144.1
**6**	112.8	111.5	112.2	112.6	113.1	116.9	116.3	111.9	118.5
**7**	162.5	160.0	162.3	162.1	162.7	160.2	159.5	160.3	159.0
**8**	113.3	112.8	116.1	113.1	113.7	107.1	110.0	113.1	106.2
**9**	158.8	157.2	160.0	158.9	159.1	155.9	157.8	157.3	159.6
**10**	117.4	115.9	118.5	117.0	117.6	114.3	114.7	115.9	113.9
**11**	124.7	43.2	44.3	44.2	76.2	47.2	47.7	47.8	48.2
**12**	139.0	63.8	66.7	66.3	69.5	202.5	202.4	202.5	200.7
**13**	18.4	23.8	23.6	23.6	19.7	22.2	22.6	30.0	30.2
**14**	23.6	22.8	23.3	23.6	23.7	29.9	29.9	23.0	23.1
**15**	56.9	56.4	-	56.7	56.9	-	-	56.6	-
**1′**	75.1	73.0	76.0	74.6	74.9	80.2	73.5	72.9	68.1
**2′**	72.4	70.9	73.2	72.7	72.9	77.5	71.0	71.1	73.7
**3′**	80.3	78.8	80.1	80.0	80.3	74.8	78.7	79.1	73.7
**4′**	72.3	70.8	71.8	71.9	72.2	80.5	70.4	70.7	70.2
**5′**	82.9	81.8	82.7	82.4	82.6	68.3	81.5	81.8	76.6
**6′**	63.0	61.7	62.8	63.0	63.3	63.8	61.4	61.8	61.6
**2** **′-OCOCH_3_**	-	-	-	-	-	-	-	-	168.6, 20.7
**3** **′-OCOCH_3_**	-	-	-	-	-	-	-	-	169.4, 20.7
**4** **′-OCOCH_3_**	-	-	-	-	-	-	-	-	170.3, 20.7
**6** **′-OCOCH_3_**	-	-	-	-	-	-	-	-	170.6, 20.7
**Solvent**	CD_3_OD	DMSO-*d*_6_	CD_3_OD	CD_3_OD	CD_3_OD	DMSO-*d*_6_	DMSO-*d*_6_	CD_3_OD	CDCl_3_
**References**	[80]	[159]	[160]	[82]	[160]	[94]	[94]	[96]	[93]

**Table 30:** The ^13^C-NMR spectral data of compounds **103**–**110** except which has no reported ^13^C-NMR data.

C	103	104	106	107	108	109	110
**2**	165.0	165.0	167.4	167.2	167.4	167.1	167.2
**3**	111.3	111.3	112.5	112.6	112.6	111.9	111.9
**4**	178.6	178.8	182.3	182.2	182.2	181.9	182.0
**5**	141.7	141.8	144.6	144.8	144.6	144.3	144.3
**6**	111.0	111.3	112.7	112.6	112.5	112.3	112.4
**7**	159.5	159.8	162.1	162.0	162.0	161.6	161.6
**8**	110.6	110.7	111.8	111.4	111.8	111.6	111.3
**9**	157.4	157.5	159.8	159.6	159.6	159.3	159.1
**10**	115.8	115.8	117.2	117.2	117.2	116.9	116.9
**11**	43.1	43.3	44.6	44.9	44.6	44.5	44.3
**12**	64.4	63.9	65.9	66.3	66.0	66.6	66.5
**13**	23.3	23.7	23.6	23.7	23.6	23.5	23.6
**14**	22.8	22.9	23.7	23.7	23.6	23.5	23.3
**15**	56.5	56.5	57.0	57.1	57.0	56.9	57.0
**1′**	70.6	70.6	72.8	72.8	72.8	71.9	71.8
**2′**	72.6	72.3	73.4	73.7	73.9	74.0	73.7
**3′**	75.7	75.9	77.7	77.7	77.9	77.6	77.4
**4′**	70.4	70.9	72.4	72.4	72.3	72.4	72.4
**5′**	81.8	82.0	83.0	80.1	82.9	82.7	82.4
**6′**	61.5	61.6	63.1	64.4	63.1	62.9	62.7
**1″**	133.9	125.0	127.0	127.0	128.2	135.3	126.7
**2″**	128.9	130.3	133.2	131.1	130.9	128.9	130.9
**3″**	128.2	115.8	115.7	116.9	115.4	129.7	116.6
**4″**	130.4	159.6	160.0	161.3	163.2	131.3	160.7
**5″**	128.2	115.8	115.7	116.9	115.4	129.7	116.6
**6″**	128.9	130.3	133.2	131.1	130.9	128.9	130.9
**7″**	144.1	144.4	145.0	146.6	146.1	146.1	146.4
**8″**	117.8	114.0	115.7	114.6	115.6	118.1	114.2
**9″**	165.0	165.4	167.6	168.0	167.8	167.2	167.8
**OCH_3_**	-	-	-	-	55.9	-	-
**COCH_3_**	-	-	-	173.0, 20.9	-	-	-
**Solvent**	DMSO-*d*_6_	DMSO-*d*_6_	CD_3_OD	CD_3_OD	CD_3_OD	CD_3_OD	CD_3_OD
**References**	[99]	[159]	[80]	[100]	[100]	[82]	[82]

**Table 31:** The ^13^C-NMR spectral data of compounds **111**–**122** except which has no reported ^13^C-NMR data.

C	111	112	113	114	115	116	117	119	120	121	122
**2**	167.2	167.6	169.7	160.3	163.0	160.1	163.1	160.6	164.0	167.6	166.8
**3**	112.1	112.2	110.5	1125	113.8	112.5	111.0	112.5	112.4	112.2	112.2
**4**	182.2	182.3	182.4	178.6	182.1	178.4	181.8	178.6	182.0	182.3	182.3
**5**	143.5	144.6	144.8	141.0	144.7	140.4	146.1	141.8	144.9	144.6	144.8
**6**	116.3	112.6	112.7	115.8	112.2	115.7	128.3	111.5	113.1	112.6	112.7
**7**	161.3	162.0	162.1	159.l	162.1	159.4	161.1	159.7	162.3	161.0	162.2
**8**	110.0	111.9	111.8	110.2	112.0	110.7	121.1	110.8	111.6	111.9	111.9
**9**	160.4	159.6	159.4	158.3	159.6	157.9	157.9	157.4	159.5	159.6	159.6
**10**	117.0	117.3	117.5	114.8	117.1	114.8	114.3	115.6	117.6	117.2	117.3
**11**	44.3	44.6	75.7	48.1	49.1	47.8	48.5	47.9	98.6	44.6	44.6
**12**	66.7	66.8	69.2	202.4	204.7	202.3	204.6	202.1	203.7	66.8	66.8
**13**	23.5	23.6	19.7	30.4	29.9	29.6	30.7	29.6	23.7	23.7	24.0
**14**	23.5	23.6	23.7	22.7	23.7	22.6	23.3	22.7	25.0	23.6	23.5
**15**	-	57.1	57.1	-	57.1	-	55.8	55.5	57.2	57.1	57.1
**1′**	73.0	72.1	72.8	70.2	72.1	73.3	75.6	70.4	72.6	72.6	72.6
**2′**	74.0	74.0	74.1	72.3	73.9	70.8	72.3	72.2	74.3	74.1	73.6
**3′**	77.9	77.9	77.8	76.0	77.9	78.5	79.9	75.8	78.0	77.8	77.6
**4′**	72.1	72.7	72.3	70.2	72.6	70.4	72.1	70.6	71.9	78.3	78.1
**5′**	82.8	82.9	83.0	81.8	82.8	78.4	78.5	81.9	83.3	82.9	82.9
**6′**	63.1	63.1	63.1	61.8	63.3	64.8	65.4	61.5	63.5	63.0	63.0
**1″**	128.3	127.5	135.7	125 I	127.0	125.0	128.3	125.5	135.7	129.8	129.7
**2″**	131.0	115.1	129.2	1301	130.8	130.3	131.0	111.1	129.3	130.8	132.6
**3″**	116.7	149.9	130.1	115.8	116.7	115.7	115.3	147.9	130.1	118.0	116.7
**4″**	161.2	146.6	131.6	159.6	161.3	159.7	160.4	149.2	131.6	163.8	159.7
**5″**	116.7	116.6	130.1	115.8	116.7	115.7	115.3	115.5	130.1	-	-
**6″**	131.0	123.0	129.2	1301	130.8	130.3	131.0	122.9	129.3	-	-
**7″**	146.2	147.1	146.4	144.4	145.3	144.9	146.2	144.6	146.3	146.0	144.7
**8″**	115.8	114.5	118.4	114.1	115.1	114.0	116.0	114.2	118.5	116.5	117.6
**9″**	168.0	168.2	167.4	165.4	168.1	166.7	169.1	165.4	167.4	167.8	167.7
**OCH_3_**	56.1	-	-	-	-	-	-	56.3	-	-	-
**1‴**	-	-	-	-	-	-	-	-	-	101.9	101.7
**2‴**	-	-	-	-	-	-	-	-	-	74.8	74.9
**3‴**	-	-	-	-	-	-	-	-	-	72.1	72.6
**4‴**	-	-	-	-	-	-	-	-	-	71.3	71.2
**5‴**	-	-	-	-	-	-	-	-	-	78.0	77.9
**6‴**	-	-	-	-	-	-	-	-	-	62.5	62.4
**Solvent**	CD_3_OD	CD_3_OD	CD_3_OD	DMSO-*d*_6_	DMSO-*d*_6_	DMSO-*d*_6_	CD_3_OD	DMSO-*d*_6_	CD_3_OD	CD_3_OD	CD_3_OD
**References**	[80]	[82]	[160]	[161]	[162]	[163]	[74]	[164]	[81]	[160]	[160]

**Table 32:** The ^13^C-NMR spectral data of compounds **123**–**129** except which has no reported ^13^C-NMR data.

C	123	124	126	127	128	129
**2**	167.7	168.3	171.3	170.7	167.7	169.9
**3**	108.0	108.5	105.6	105.5	108.3	108.4
**4**	182.2	182.4	184.3	183.0	182.7	182.4
**5**	158.8	157.8	160.2	158.1	159.5	159.6
**6**	110.4	111.1	112.5	112.8	107.4	105.2
**7**	162.3	163.1	163.8	161.0	162.9	163.1
**8**	93.2	90.5	94.4	93.0	92.8	92.9
**9**	156.1	156.6	157.8	156.1	156.7	156.4
**10**	103.3	104.4	106.8	105.6	103.4	103.8
**11**	19.9	19.9	61.6	60.0	18.8	60.0
**OCH_3_**	-	56.5	-	-	-	-
**1′**	20.5	20.5	22.1	20.5	26.4	26.4
**2′**	126.9	126.4	128.7	124.6	79.9	79.8
**3′**	131.8	132.3	132.9	134.1	143.7	143.7
**4′**	66.1	66.1	68.4	61.0	114.2	114.2
**5′**	21.2	21.3	21.9	20.0	15.2	15.2
**1″**	101.5	101.6	102.7	100.1	99.4	99.4
**2″**	73.6	73.6	75.3	73.2	73.6	73.6
**3″**	76.9	77.0	78.3	76.7	76.3	76.3
**4″**	70.2	70.2	71.8	67.7	70.2	70.2
**5″**	77.0	77.0	77.9	77.0	76.7	76.7
**6″**	61.1	61.1	62.9	60.1	61.2	61.2
**Solvent**	DMSO-*d_6_*	DMSO-*d_6_*	CD_3_OD	CD_3_OD	CD_3_OD	CD_3_OD
**References**	[104]	[104]	[106]	[60]	[60]	[60]

**Table 33:** The ^13^C-NMR spectral data of compounds **130**–**139**.

C	130	131	132	133	134	135	136	137	138	139
**2**	170.7	167.6	170.7	168.3	171.4	168.9	165.0	171.0	171.5	171.8
**3**	105.5	107.4	104.9	108.1	105.5	113.9	*	110.4	110.7	110.7
**4**	183.0	181.8	181.9	182.5	182.5	176.6	*	179.3	180.4	180.6
**5**	158.8	158.8	158.9	159.5	159.5	132.6	*	106.6	119.2	126.2
**6**	111.4	97.6	97.8	98.3	98.4	111.4	99.3	150.7	126.2	126.6
**7**	162.1	159.9	160.1	160.6	160.8	158.2	162.9	153.1	122.1	135.6
**8**	93.3	107.5	107.4	108.1	108.2	103.6	94.8	106.9	147.8	119.3
**9**	156.3	153.8	153.5	154.4	154.1	163.4	*	149.8	149.0	158.0
**10**	105.7	104.3	104.8	105.0	105.4	112.7	*	116.2	125.0	124.3
**11**	60.0	19.5	59.3	20.2	59.5	127.9	121.6	102.2	141.4	136.2
**1′**	28.2	20.3	20.3	21.0	20.9	131.9	128.5	74.3	129.5	114.0
**2′**	74.6	120.5	120.4	121.1	121.0	115.7	115.7	78.1	129.6	150.7
**3′**	147.9	134.9	134.9	135.6	135.7	157.1	161.5	71.5	127.4	146.4
**4′**	109.5	65.9	65.8	66.5	66.5	115.7	115.7	78.0	129.6	118.1
**5′**	16.6	13.1	13.1	13.8	13.7	131.9	128.5	62.8	129.5	122.0
**6′**	-	-	-	-	-	39.3	39.1	-	33.8	33.6
**7′**	-	-	-	-	-	86.3	37.5	-	37.0	37.1
**8′**	-	-	-	-	-	127.9	121.6	102.2	141.4	136.2
**1″**	101.2	100.1	100.1	100.7	100.7	101.8	100.3	131.5	103.0	102.9
**2″**	73.6	72.9	72.9	73.5	73.5	74.6	*	133.0	75.0	74.9
**3″**	76.5	76.7	76.7	76.7	76.7	78.4	*	116.3	78.2	77.8
**4″**	69.8	69.2	69.2	69.8	69.8	71.2	*	164.1	71.3	71.3
**5″**	77.1	76.1	76.1	77.3	77.3	78.2	*	116.3	78.2	78.1
**6″**	61.1	60.2	60.2	60.8	60.8	62.5	*	120.9	62.5	62.4
**7″**	-	-	-	-	-	-	-	33.9	-	-
**8″**	-	-	-	-	-	-	-	37.1	-	-
**OCH_3_**	-	-	-	-	-	-	-	6-OCH_3_ 56.3 4″-OCH_3_ 55.1	-	56.6
**Solvent**	CD_3_OD	DMSO-*d_6_*	DMSO-*d_6_*	DMSO-*d_6_*	DMSO-*d_6_*	CD_3_OD	DMSO-*d_6_*	DMSO-*d_6_*	CD_3_OD	CD_3_OD
**References**	[60]	[108]	[108]	[109]	[109]	[110]	[111]	[112]	[113]	[114]

* The authors missed assigning these positions.

**Table 34:** The ^13^C-NMR spectral data of compounds **140**–**148**.

C	140	141	142	143	144	145	146	147	148
**2**	147.2	146.9	147.3	89.9	89.0	92.9	92.1	92.3	88.2
**3**	106.1	105.0	104.9	24.3	28.3	28.1	27.9	28.0	30.1
**4**	153.7	160.5	159.2	165.5	164.5	160.3	165.3	156.3	158.5
**5**	177.2	185.9	184.7	176.9	176.4	184.7	176.6	176.6	182.9
**6**	109.7	107.8	107.2	111.9	109.8	109.5	110.9	111.4	107.2
**7**	163.7	169.0	168.7	163.3	164.0	169.7	162.7	162.6	166.7
**9**	95.8	92.0	92.0	94.8	95.2	90.4	94.0	94.2	88.8
**10**	157.8	156.1	154.5	159.9	160.1	168.3	159.7	165.4	166.6
**11**	117.0	114.3	113.4	118.7	117.2	111.3	118.1	118.5	108.7
**12**	152.8	106.9	106.3	112.3	111.4	106.6	112.5	112.8	102.9
**13**	155.6	155.7	155.2	156.4	157.7	157.9	156.1	159.9	158.6
**14**	65.7	67.8	66.3	21.4	20.7	20.8	66.4	66.8	66.1
**4-OCH_3_**	61.9	-	-	-	*	-	60.8	61.0	-
**1′**	103.0	104.4	104.4	77.3	74.3	79.7	70.8	70.8	143.7
**2′**	74.0	75.0	74.2	23.4	24.1	24.3	26.0	26.2	115.5
**3′**	77.2	77.9	77.6	22.3	23.5	23.0	25.6	25.8	15.7
**4′**	70.6	71.6	70.9	-	-	-	-	-	-
**5′**	77.1	78.0	76.7	-	-	-	-	-	-
**6′**	61.7	68.7	69.5	-	-	-	-	-	-
**1″**	-	111.1	103.3	98.9	100.1	99.5	104.0	104.4	102.8
**2″**	-	77.2	74.4	75.1	74.6	75.6	74.8	75.0	73.7
**3″**	-	80.6	77.4	78.8	78.6	78.5	78.4	78.3	76.7
**4″**	-	74.9	70.9	71.3	71.4	72.0	71.4	71.3	70.3
**5″**	-	65.5	77.7	77.1	78.2	76.8	78.1	75.7	76.9
**6″**	-	-	61.9	62.4	63.1	68.5	62.6	64.5	61.4
**1‴**	-	-	-	-	-	111.2	-	126.5	-
**2‴**	-	-	-	-	-	78.6	-	111.4	-
**3‴**	-	-	-	-	-	81.0	-	149.0	-
**4‴**	-	-	-	-	-	75.6	-	151.2	-
**5‴**	-	-	-	-	-	66.2	-	116.8	-
**6‴**	-	-	-	-	-	-	-	123.9	-
**7‴**	-	-	-	-	-	-	-	145.9	-
**8‴**	-	-	-	-	-	-	-	115.1	-
**9‴**	-	-	-	-	-	-	-	167.8	-
**3‴-OCH_3_**	-	-	-	-	-	-	-	55.9	-
**Solvent**	DMSO-*d*_6,_ C_6_D_6_	CD_3_OD	DMSO-*d*_6_	CDCl_3_, CD_3_OD	CDCl_3_, CD_3_OD	CD_3_OD	C_5_D_5_N	C_5_D_5_N	CD_3_OD
**References**	[117]	[118]	[109]	[122]	[122]	[119]	[121]	[132]	[60]

* The authors missed assigning this position.

**Table 35:** The ^13^C-NMR spectral data of compounds **149**–**155**.

C	149	150	151	152	153	154	155
**2**	167.4	169.5	162.7	167.6	167.6	170.9	168.7
**3**	108.7	108.0	111.1	107.5	107.4	105.6	108.6
**4**	182.7	184.2	175.3	181.9	181.8	182.5	182.6
**5**	160.0	160.9	160.7 *	158.6	158.5	159.2	160.3
**6**	104.1	105.0	104.9	103.1	103.1	103.8	102.5
**7**	159.6	160.4	152.7 *	163.5	163.3	164.1	164.3
**8**	94.9	95.8	91.1	109.8	109.9	110.5	105.8
**9**	156.3	157.7	157.8	152.9	152.7	153.1	155.0
**10**	104.4	105.0	107.7	105.5	105.4	106.5	106.1
**2′**	78.4	79.3	76.6	69.6	69.6	70.2	74.2
**3′**	74.3	75.0	72.4	135.2	135.0	135.8	73.5
**4′**	22.3	22.7	22.4	124.7	124.7	125.2	132.7
**5′**	-	-	-	20.6	20.3	20.9	117.3
**1″**	102.4	102.0	100.4	101.2	100.9	101.9	103.9
**2″**	74.9	74.9	**	72.8	72.6	73.3	73.7
**3″**	78.4	78.1	76.9	76.8	76.1	76.8	76.5
**4″**	71.8	71.9	70.2	70.1	69.4	70.1	70.9
**5″**	78.4	75.0	76.9	76.6	75.2	76.6	76.0
**6″**	63.0	68.8	61.3	60.6	67.7	61.6	68.5
**1‴**	-	111.0	-	-	102.7	-	103.4
**2‴**	-	77.9	-	-	72.8	-	73.7
**3‴**	-	80.5	-	-	76.1	-	76.9
**4‴**	-	75.0	-	-	69.4	-	70.0
**5‴**	-	65.7	-	-	75.9	-	77.0
**6‴**	-	-	-	-	60.4	-	61.1
**2a**	20.1	20.4	19.0	19.4	19.2	59.7	20.1
**2’a**	22.3	22.4	21.2	-	-	-	-
**2’b**	25.7	26.1	25.2	-	-	-	-
**3’a**	-	-	-	69.6	69.6	70.2	73.0
**7-OCH_3_**	-	-	56.0	-	-	-	-
**Solvent**	CDCl_3_	CD_3_OD	DMSO-*d*_6_	DMSO-*d*_6_	DMSO-*d*_6_	DMSO-*d*_6_	DMSO-*d*_6_
**References**	[121]	[119]	[123]	[108]	[108]	[108]	[109]

* Interchangeable data. ** The authors missed the assignment of this position.

**Table 36:** The ^13^C-NMR spectral data of compounds **157**–**165**.

C	157	158	159	160	161	162	163	164	165
**Chromone** **moiety**									
**2′**	92.4	92.2	92.2	92.2	92.2	92.2	92.2	92.2	92.2
**3′**	27.9	27.8	27.8	27.8	27.8	27.8	27.8	27.8	27.8
**4′**	156.3	156.2	156.2	156.2	156.2	156.2	156.2	156.2	156.1
**5′**	176.2	176.2	176.2	176.2	176.2	176.2	176.3	176.3	176.3
**6′**	111.5	110.9	111.1	110.9	110.9	110.8	111.1	111.2	111.1
**7′**	162.1	162.5	162.4	162.4	162.7	162.5	162.6	162.6	162.5
**9′**	94.1	94.0	94.0	94.0	94.1	94.0	94.0	94.1	94.0
**10′**	165.4	165.2	165.2	165.2	165.2	165.2	165.2	165.2	165.2
**11′**	118.5	118.4	118.4	118.4	118.4	118.4	118.4	118.4	118.4
**12′**	112.9	112.8	112.8	112.5	112.8	112.8	112.8	112.8	112.7
**13′**	159.9	159.8	159.8	159.7	159.8	159.7	159.8	159.9	159.8
**14′**	66.3	66.3	66.5	66.2	66.4	66.2	66.5	66.3	66.5
**4-OCH_3_**	60.9	60.9	60.9	60.8	60.9	60.9	60.9	60.9	60.8
**1″**	70.6	70.6	70.6	70.6	70.6	70.6	70.6	70.7	70.6
**2″**	26.0	26.1	26.1	26.1	26.1	26.1	26.1	26.2	26.1
**3″**	25.6	25.7	25.7	25.6	25.7	25.7	25.6	25.6	25.7
**Glu-1**	101.7	104.1	104.2	104.1	104.1	104.0	104.2	103.9	104.3
**Glu-2**	74.5	74.9	74.8	74.8	74.9	74.9	74.9	74.8	74.9
**Glu-3**	79.1	78.3	78.1	78.4	78.4	78.4	78.4	78.2	78.1
**Glu-4**	69.3	71.2	71.4	71.4	71.5	71.3	71.5	72.0	71.9
**Glu-5**	78.4	76.9	75.4	77.1	77.1	76.7	77.0	76.8	76.7
**Glu-6**	62.4	63.5	64.2	62.7	62.7	62.8	62.6	62.9	62.7
**Triterpene** **moiety**									
**1**	31.8	31.9	31.9	32.3	30.3	32.0	27.4	31.9	31.9
**2**	29.8	29.8	30.0	30.0	29.5	30.0	29.8	29.6	29.8
**3**	87.8	88.0	88.3	88.4	88.1	88.3	88.3	88.0	88.0
**4**	41.1	40.3	41.2	41.2	41.2	41.2	40.7	41.1	41.1
**5**	47.1	47.0	47.4	47.4	42.6	47.4	43.8	47.0	46.9
**6**	20.5	20.4	20.8	20.9	21.7	20.8	22.0	20.4	20.3
**7**	25.6	25.8	25.9	26.4	113.2	26.3	114.0	25.8	25.7
**8**	46.0	45.9	47.1	49.0	149.1	47.3	148.6	45.8	46.4
**9**	19.8	19.9	19.2	19.9	21.1	19.5	27.6	20.0	20.5
**10**	26.5	26.6	26.6	26.5	28.2	26.4	29.1	26.6	27.0
**11**	36.7	36.6	26.3	26.4	25.4	26.1	63.2	36.7	36.4
**12**	77.2	76.8	31.4	34.0	33.9	33.3	48.3	77.2	76.9
**13**	48.8	48.7	42.2	41.8	41.2	46.6	45.4	48.7	48.8
**14**	47.2	47.4	45.2	46.5	49.8	45.1	48.1	47.8	47.8
**15**	43.4	43.4	50.8	82.5	80.4	42.8	45.2	43.8	45.6
**16**	70.7	72.8	218.6	103.0	103.3	72.5	114.5	71.1	74.6
**17**	55.7	55.6	60.4	60.5	60.3	51.4	61.0	56.8	52.4
**18**	13.5	13.5	18.8	20.3	22.5	20.5	20.7	13.5	12.9
**19**	29.9	30.7	30.0	30.7	28.2	30.1	18.7	29.8	30.0
**20**	26.9	25.4	29.1	27.6	27.5	34.2	23.7	26.0	24.6
**21**	21.1	20.8	19.9	21.4	21.6	17.3	19.6	21.1	25.7
**22**	41.5	38.6	40.3	33.6	33.6	86.2	37.8	41.9	106.0
**23**	104.2	102.9	173.4	74.2	74.4	109.6	71.7	101.9	152.9
**24**	83.1	212.4	-	80.5	80.4	77.3	88.5	77.7	75.9
**25**	79.1	35.0	-	76.4	76.5	83.5	76.4	81.3	78.2
**26**	19.4	19.2	-	21.6	21.6	27.3	23.2	23.7	22.3
**27**	29.8	19.7	-	24.1	24.1	24.5	20.2	24.9	23.1
**28**	19.4	19.4	19.6	11.8	18.2	19.4	27.4	19.5	20.7
**29**	25.6	25.6	25.6	25.5	25.6	25.6	25.8	25.6	25.6
**30**	15.2	15.2	15.2	15.5	14.2	15.3	14.5	15.2	15.2
**Xyl-1**	107.4	107.5	107.5	107.4	107.4	107.4	107.4	107.5	107.5
**Xyl-2**	75.5	75.5	75.5	75.5	75.5	75.5	75.5	75.5	75.5
**Xyl-3**	78.5	78.6	78.5	78.5	78.5	78.5	78.5	78.5	78.5
**Xyl-4**	71.1	71.0	71.1	71.1	71.2	71.1	71.1	71.2	71.1
**Xyl-5**	67.0	67.0	67.1	67.0	67.0	67.0	67.0	67.0	67.0
**COCH_3_**	170.4 21.5	170.4 21.5	-	171.0 21.1	171.0 21.1	-	-	170.5 21.6	170.6 21.1
**Solvent**	C_5_D_5_N	C_5_D_5_N	C_5_D_5_N	C_5_D_5_N	C_5_D_5_N	C_5_D_5_N	C_5_D_5_N	C_5_D_5_N	C_5_D_5_N
**References**	[130]	[130]	[130]	[130]	[130]	[130]	[130]	[136]	[136]

**Table 37:** The ^13^C-NMR spectral data of compounds **166** and **167**.

C	166	167
**1**	98.1	98.1
**3**	154.5	154.5
**4**	105.6	105.6
**5**	28.7	28.6
**6**	30.0	29.9
**7**	75.1	75.0
**8**	133.3	133.3
**9**	43.9	43.8
**10**	121.1	121.1
**11**	168.6	168.5 ^a^
**1′**	99.8	99.7
**2′**	74.8	74.7 ^b^
**3′**	77.9	77.8 ^c^
**4′**	71.6	71.2 ^d^
**5′**	78.5	78.4 ^e^
**6′**	62.7	62.4 ^f^
**2″**	167.5	168.1 ^a^
**3″**	118.2	118.6
**4″**	181.6	181.8
**5″**	163.5	163.1
**6″**	100.2	101.1
**7″**	166.2	164.9
**8″**	94.7	95.7
**9″**	159.2	158.7
**10″**	104.9	106.6
**-CH_3_**	18.9	19.0
**1‴**	-	101.6
**2‴**	-	74.8 ^b^
**3‴**	-	77.9 ^c^
**4‴**	-	71.6 ^d^
**5‴**	-	78.5 ^e^
**6‴**	-	62.7 ^f^
**NMR**	CD_3_OD	CD_3_OD
**References**	[43]	[43]

^a−f^ Values with the same superscript are interchangeable.

**Table 38:** The ^13^C-NMR spectral data of compounds **168**–**180** except those which have no reported ^13^C-NMR data.

C	168	169	170	171	172	178	179	180
**2**	163.7	159.3	168.0	159.6	167.5	170.2	166.3	166.3
**3**	108.7	108.8	109.7	109.9	110.0	110.8	110.0	110.0
**4**	179.2	179.5	178.9	178.8	179.0	179.0	178.7	178.8
**4a**	125.8	126.2	125.9 *	126.4	125.9 *	125.8 *	125.8 *	126.5
**5**	149.6	149.6	149.9	150.0	149.8	150.1	149.7	149.9
**6**	125.4	125.4	125.8	125.9	125.9 *	125.8 *	125.9	126.0
**6a**	137.0	137.2	137.0	137.0	137.4	137.2	137.3	136.3
**7**	183.0	183.3	183.2	183.5	183.3	183.1	183.1	181.5
**7a**	125.8	127.3	126.3 *	126.5	126.4 *	126.1 *	126.2 *	130.7
**8**	140.0	140.6	139.7	140.0	140.1	139.9	140.2	119.3
**9**	133.0	132.6	132.9	132.2	133.1	133.3	133.1	133.2
**10**	138.4	138.5	137.8	137.0	138.5	138.6	138.6	140.7
**11**	159.7	163.9	159.7	167.6	159.9	159.9	159.8	159.5
**11a**	116.0	115.8	116.1	116.0	116.2	116.0	116.1	116.1
**12**	188.1	188.0	187.9	187.7	188.1	188.3	188.0	187.7
**12a**	118.9	118.9	119.1	119.1	119.2	118.9	119.2	119.8
**12b**	155.7	155.8	156.0	156.0	156.2	155.8	156.1	156.2
**13**	24.0	24.1	24.3	24.2	24.1	24.2	24.1	24.1
**14**	127.2	125.9	57.6	60.3	59.1	75.6	57.7	57.7
**15**	12.1	15.0	13.8	14.9	14.5	23.5	14.5	14.4
**16**	134.2	134.2	62.0	61.7	61.6	71.7 *	63.9	63.9
**17**	14.9	12.1	14.1	123.3	123.3	127.4	55.4	55.4
**18**	-	-	-	134.1	134.0	130.2	51.8	51.8
**19**	-	-	-	14.4	13.8	13.5	17.2	17.2
**2′**	77.3	77.7	77.2	77.6	77.3	77.2	77.3	67.9
**3′**	71.9	71.8	71.3	70.6	71.9	71.7 *	71.9	71.0
**4′**	67.4	67.5	67.8	68.1	67.4	67.3 *	67.4	57.7
**5′**	28.3	41.0	28.8	38.8	28.4	28.4	28.3	35.0
**6′**	75.2	75.4	74.8	74.0	75.2	75.0	75.2	68.2
**7′**	18.9	18.9	18.8	18.5	18.9	18.9	18.9	17.5
**8′**	-	-	-	-	-	-	-	13.2
**4′-N(CH_3_)_2_**	40.4	40.5	40.3	40.4	40.4	40.3	40.4	37.1
**2″**	67.2	69.8	67.5	69.8	67.2	67.4 *	67.3	-
**3″**	70.8	76.4	70.3	74.5	70.9	70.7	70.9	-
**4″**	57.4	57.6	57.6	59.1	57.3	57.9	57.3	-
**5″**	33.6	28.6	33.3	30.4	33.6	33.3	33.7	-
**6″**	69.5	64.9	69.4	65.7	69.7	69.6	69.6	-
**7″**	17.6	15.0	17.4	15.1	17.7	17.6	17.6	-
**8″**	12.3	13.7	13.1	13.9	12.3	12.6	12.3	-
**3″-COCH_3_**	-	170.6 21.3	-	171.0 21.5	-	-	-	-
**4″-N(CH_3_)_2_**	36.8	39.3	36.9	38.8	36.8	36.8	36.8	-
**Solvent**	CDCl_3_	CDCl_3_	CDCl_3_	CDCl_3_	CDCl_3_	CDCl_3_	CDCl_3_	CDCl_3_
**References**	[165]	[140]	[141]	[140]	[142]	[143]	[165]	[146]

* Interchangeable values.

**Table 39:** The ^13^C-NMR spectral data of compounds **181**–**183**.

C	181	182	183
**2**	168.4	176.4	176.5
**3**	112.0	111.1	111.3
**4**	180.3	181.3	178.4
**4a**	126.1	126.0	124.7
**5**	150.4	150.4	149.6
**6**	121.3	121.0	121.2
**6a**	138.3	138.1	139.0
**7**	182.7	182.7	182.4
**7a**	133.2	133.2	133.2
**8**	120.1	120.0	119.8
**9**	135.4	135.5	135.6
**10**	137.4	137.4	137.0
**11**	161.1	161.0	161.3
**11a**	118.1	118.0	118.0
**12**	189.1	189.2	189.2
**12a**	122.0	121.9	121.6
**12b**	157.7	157.4	156.9
**13**	70.8	70.8	175.3
**14**	61.2	77.7	77.7
**15**	63.7	72.6	72.6
**16**	20.2	23.9	23.9
**17**	13.7	17.0	17.1
**1′**	98.9	98.9	-
**2′**	37.2	37.2	-
**3′**	58.3	58.4	-
**4′**	71.2	71.1	-
**5′**	70.4	70.4	-
**6′**	17.2	17.2	-
**7′**	24.6	24.5	-
**1″**	70.2	70.1	70.3
**2″**	28.0	27.7	27.4
**3″**	65.2	65.1	64.9
**4″**	75.1	75.0	75.2
**5″**	72.6	72.6	72.3
**6″**	18.1	18.2	18.3
**3″-N(CH_3_)_2_**	43.2 41.7	43.3 41.4	42.5 42.5
**1‴**	101.4	101.4	101.4
**2‴**	33.4	33.4	33.4
**3‴**	66.6	66.6	66.6
**4‴**	72.0	72.0	72.0
**5‴**	69.5	69.5	69.5
**6‴**	17.5	17.5	17.5
**Solvent**	CD_3_OD	CD_3_OD	CD_3_OD
**References**	[147]	[147]	[147]

**Table 40:** The ^13^C-NMR spectral data of compounds **184**–**192**.

C	184	185	186	187	188	189	190	191	192
**2**	167.5	167.4	167.0	167.0	165.7	165.7	167.5	169.3	169.3
**3**	111.1	110.9	110.9	110.8	111.3	111.3	111.1	109.1	109.1
**4**	180.2	180.0	179.4	179.4	178.5	178.6	180.2	182.4	182.5
**4a**	126.6	126.4	126.5	126.4	126.3	126.3	126.6	113.3	113.3
**5**	149.2	149.1	149.4	149.2	148.2	148.2	149.3	166.7	166.7
**6**	122.5	122.4	122.9	122.8	124.1	124.0	122.5	110.7	110.7
**6a**	137.2	137.1	137.0	136.9	136.9	136.9	137.2	139.8	139.9
**7**	181.2	181.0	181.2	181.2	181.5	181.4	181.2	180.8	180.8
**7a**	130.3	130.2	130.4	130.3	130.5	130.5	130.5	130.6	130.4
**8**	119.8	119.7	119.7	119.7	119.5	119.5	119.8	119.4	119.5
**9**	133.6	133.7	133.4	133.6	133.3	133.5	133.7	132.4	132.8
**10**	141.3	141.1	141.0	140.9	141.0	140.9	140.7	140.2	140.9
**11**	159.1	159.2	159.3	158.9	159.3	159.3	159.4	159.1	159.1
**11a**	115.8	115.6	115.9	115.7	115.9	115.9	115.9	115.6	115.4
**12**	186.9	186.9	187.1	186.9	187.5	187.4	186.9	186.2	186.3
**12a**	121.8	121.6	121.6	121.6	120.8	120.8	121.8	112.3	112.4
**12b**	156.8	156.7	156.6	156.5	156.1	156.1	156.8	156.6	156.6
**13**	80.9	80.0	79.0	78.9	48.3	48.2	80.9	-	-
**14**	59.8	59.7	59.8	59.7	59.8	59.7	59.8	60.0	60.0
**15**	19.7	19.6	19.8	19.7	20.0	19.9	19.6	19.6	19.7
**16**	62.7	62.5	62.5	62.5	62.5	62.5	62.7	62.7	62.7
**17**	13.4	13.3	13.4	13.3	13.5	13.4	13.3	13.2	13.2
**18**	170.5	170.4	170.9	170.9	170.4	170.4	170.5	-	-
**19**	52.6	52.5	52.4	52.3	52.3	52.3	52.6	-	-
**2′**	73.8	73.8	74.9	74.9	74.3	74.2	73.8	-	-
**3′**	68.9	68.9	68.5	68.2	68.8	68.8	69.0	-	-
**4′**	80.2	80.1	74.8	74.9	81.5	81.4	80.2	-	-
**5′**	67.9	67.9	26.1	26.0	68.0	67.9	68.0	-	-
**6′**	73.7	73.6	70.3	70.2	73.9	74.0	73.7	-	-
**7′**	14.1	14.0	14.7	14.7	14.7	14.6	14.0	-	-
**4′-OCH_3_**	57.9	57.8	55.6	55.5	57.1	57.1	58.0	-	-
**2″**	70.3	70.7	70.2	70.8	70.3	70.8	70.0	70.0	70.7
**3″**	77.7	82.6	77.8	82.7	77.8	82.8	76.0	77.3	82.8
**4″**	54.9	58.1	55.1	58.1	54.9	58.1	51.7	56.1	58.1
**5″**	40.3	44.7	40.4	44.7	40.4	44.8	44.9	38.5	44.8
**6″**	62.1	62.2	62.3	62.2	62.2	62.3	62.3	63.6	62.3
**7″**	14.7	13.5	14.8	13.5	14.7	13.6	14.1	15.4	13.6
**8″**	24.1	14.0	23.8	13.9	24.2	14.1	32.6	22.6	14.0
**4″-N(CH_3_)_n_**	27.9	40.3	27.8	40.3	28.1	40.4	-	27.2	40.3
**1‴**	93.4	94.4	93.7	94.5	93.4	94.5	93.3	94.5	94.5
**2‴**	30.8	31.1	30.9	31.1	30.9	31.1	30.8	31.1	31.1
**3‴**	74.8	74.9	74.8	74.9	74.9	75.0	74.8	75.0	75.0
**4‴**	72.1	72.1	72.1	72.0	72.2	72.2	72.2	71.5	72.2
**5‴**	65.4	65.0	65.7	65.0	65.4	65.1	65.4	66.6	65.0
**6‴**	17.7	17.6	17.7	17.6	17.8	17.7	17.8	17.5	17.7
**3‴-OCH_3_**	55.9	56.1	56.1	56.1	55.9	56.2	56.0	56.4	56.1
**Solvent**	CDCl_3_	CDCl_3_	CDCl_3_	CDCl_3_	CDCl_3_	CDCl_3_	CDCl_3_	CDCl_3_	CDCl_3_
**References**	[149]	[149]	[149]	[149]	[137]	[137]	[137]	[137]	[137]

## Data Availability

Not applicable.

## Abbreviations

All	Allose
Api	Apiose
Ara	Arabinose
Caf	Caffeic acid
Cin	Cinnamic acid
Cou	Coumaric acid
Fer	Ferulic acid
Gall	Gallic acid
Glu	Glucose
Rha	Rhamnose
Xyl	Xylose

## References

[1] Herath H.M.T.B., Jacob M., Wilson A.D., Abbas H.K., Nanayakkara N.P.D. (2013). New secondary metabolites from bioactive extracts of the fungus *Armillaria tabescens*. Nat. Prod. Res..

[2] Choi S., Park Y.-I., Lee S.-K., Kim J.-E., Chung M.-H. (2002). Aloesin inhibits hyperpigmentation induced by UV radiation. Clin. Exp. Dermatol..

[3] Machado N.F.L., Marques M.P.M. (2010). Bioactive Chromone Derivatives-Structural Diversity. Curr. Bioact. Compd..

[4] Daniel M. (2006). Medicinal Plants: Chemistry and Properties.

[5] Mander L., Liu H.-W. (2010). Comprehensive Natural Products II: Chemistry and Biology.

[6] Dewick P.M. (2002). Medicinal Natural Products: A Biosynthetic Approach.

[7] Daily A., Seligmann O., Nonnenmacher G., Fessler B., Wong S., Wagner H. (1988). New chromone, coumarin, and coumestan derivatives from *Mutisia acuminata* var. *hirsuta*. Planta Med..

[8] Tschesche R., Delhvi S., Sepulveda S., Breitmaier E. (1979). Eucryphin, a new chromone rhamnoside from the bark of *Eucryphia cordifolia*. Phytochemistry.

[9] Li Z., Li D., Owen N.L., Len Z., Cao Z., Yi Y. (1996). Structure determination of a new chromone glycoside by 2D INADEQUATE NMR and molecular modeling. Magn. Reson. Chem..

[10] Feng Z., Wang Y., Zhang P. (2005). Chemical constituents of *Rhododendron ovatum* Planch. Yaoxue Xuebao.

[11] Kuo L.-M.Y., Zhang L.-J., Huang H.-T., Lin Z.-H., Liaw C.-C., Cheng H.-L., Lee K.-H., Morris-Natschke S.L., Kuo Y.-H., Ho H.-O. (2013). Antioxidant Lignans and Chromone Glycosides from *Eurya japonica*. J. Nat. Prod..

[12] Chen G., Jin H.Z., Li X.F., Zhang Q., Shen Y.H., Yan S.K., Zhang W.D. (2008). A new chromone glycoside from *Rhododendron spinuliferum*. Arch. Pharm. Res..

[13] Han L.K., Ninomiya H., Taniguchi M., Baba K., Kimura Y., Okuda H. (1998). Norepinephrine-augmenting lipolytic effectors from *Astilbe thunbergii* rhizomes. J. Nat. Prod..

[14] Sumiyoshi M., Kimura Y. (2010). Enhancing effects of a chromone glycoside, eucryphin, isolated from Astilbe rhizomes on burn wound repair and its mechanism. Phytomedicine.

[15] Liao Y.Y. (2014). A new chromone glycoside from leaves of *Salix matsudana*. Chinese Tradit. Herb. Drugs.

[16] Tane P., Ayafor J.F., Sondengam B.L., Connolly J.D. (1990). Chromone glycosides from *Schumanniophyton magnificum*. Phytochemistry.

[17] Kumar V., Gupta M., Gandhi S.G., Bharate S.S., Kumar A., Vishwakarma R.A., Bharate S.B. (2017). Anti-inflammatory chromone alkaloids and glycoside from *Dysoxylum binectariferum*. Tetrahedron Lett..

[18] Al-Taweel A., Alqasoumi S., Alam P., Abdel-Kader M. (2013). Densitometric-high-performance thin-layer chromatographic estimation of diosmin, hesperidin, and ascorbic acid in pharmaceutical formulations. J. Planar Chromatogr. Mod. TLC.

[19] Fawzy G.A., Al-Taweel A.M., Perveen S., Khan S.I., Al-Omary F.A. (2017). Bioactivity and chemical characterization of *Acalypha fruticosa* Forssk. growing in Saudi Arabia. Saudi Pharm. J..

[20] Sueyoshi E., Yu Q., Matsunami K., Otsuka H. (2008). Staphylosides A and B: Two new chromone diglucosides from leaves of *Staphylea bumalda* DC. Heterocycles.

[21] Elgorashi E.E., Coombes P.H., Mulholland D.A., Van Staden J. (2007). Isoeugenitol, a cyclooxygenase-1 inhibitor from *Gethyllis ciliaris*. South African J. Bot..

[22] Han L., Zheng F., Zhang Y., Liu E., Li W., Xia M., Wang T., Gao X. (2015). Triglyceride accumulation inhibitory effects of new chromone glycosides from *Drynaria fortunei*. Nat. Prod. Res..

[23] Isaka M., Haritakun R., Supothina S., Choowong W., Mongkolsamrit S. (2014). N-Hydroxypyridone alkaloids, chromone derivatives, and tetrahydroxanthones from the scale-insect pathogenic fungus *Orbiocrella* sp. BCC 33248. Tetrahedron.

[24] Ma L., Ma S., Wei F., Lin R., But P.P., Lee S.H., Lee S.F. (2003). Uncinoside A and B, two new antiviral chromone glycosides from *Selaginella uncinata*. Chem. Pharm. Bull. (Tokyo).

[25] Arakawa Y., Chiji H., Izawa M. (1983). Structural elucidation of two new chromones isolated from glasswort (*Salicornia europaea* L.). Agric. Biol. Chem..

[26] Chen X.-Q., Zan K., Liu H., Yang J., Lai M.-X., Wang Q. (2009). Triterpenes and flavonoids from *Ilex hainanensis* Merr. (Aquifoliaceae). Biochem. Syst. Ecol..

[27] Simon A., Chulia A.J., Kaouadji M., Delage C. (1994). Quercetin 3-[triacetylarabinosyl(1→6)galactoside] and chromones from *Calluna vulgaris*. Phytochemistry.

[28] Cui C.B., Tezuka Y., Kikuchi T., Nakano H., Tamaoki T., Park J.H. (1992). Constituents of a fern, *Davallia mariessi* Moore. IV. Isolation and structures of a novel norcarotane sesquiterpene glycoside, a chromone glucuronide, and two epicatechin glycosides. Chem. Pharm. Bull. (Tokyo).

[29] Li X., Yu M., Meng D., Li Z., Zhang L. (2007). A new chromone glycoside from *Polygonum capitatum*. Fitoterapia.

[30] Murata T., Selenge E., Suganuma K., Asai Y., Batkhuu J., Yoshizaki F. (2013). Chromone acyl glucosides and an ayanin glucoside from *Dasiphora parvifolia*. Phytochem. Lett..

[31] Yu J., Song X., Wang D., Wang X., Wang X. (2017). Five new chromone glycosides from *Scindapsus officinalis* (Roxb.) Schott. Fitoterapia.

[32] Cisowski W. (1986). 2-Methyl-5,7-dihydroxychromone glucoside from *Ammi visnaga* Lamarck. Pol. J. Chem..

[33] Wang Y.-B., Huang R., Zhang H.-B., Li L. (2006). Chromone glycosides from *Knoxia corymbosa*. J. Asian Nat. Prod. Res..

[34] Ghosal S., Kumar Y., Singh S., Ahad K. (1983). Biflorin, a chromone-C-glucoside from *Pancratium biflorum*. Phytochemistry.

[35] Ghosal S., Singh S., Bhagat M.P., Kumar Y. (1980). Three chromones from bulbs of *Pancratium biflorum*. Phytochemistry.

[36] Ibrahim S., Mohamed G., Shaala L., Youssef D. (2014). Non-alkaloidal compounds from the bulbs of the Egyptian plant *Pancratium maritimum*. Z. Naturforsch. C..

[37] Kozowska V., Zheleva A., Pangarova T. (1981). 7-(β-d-Glucopyranosyloxy)-5-hydroxy-2-methyl chromone from *Peucedanum austriacum*. Proceedings of the International Conference of Chemistry and Biotechnology of Biologically Active Natural Products, Budapest, Hungary, 15–19 August 1983.

[38] Gujral V.K., Gupta S.R., Verma K.S. (1979). A new chromone glucoside from *Tecomella undulata*. Phytochemistry.

[39] Brown R.T., Blackstock W.P., Chapple C.L. (1975). Isolation of 5,7-dihydroxy-2-methylchromone and its 7-*O*-glycosides from *Adina rubescens*. J. Chem. Soc. Perkin Trans..

[40] Peng B., Bai R.-F., Li P., Han X.-Y., Wang H., Zhu C.-C., Zeng Z.-P., Chai X.-Y. (2016). Two new glycosides from *Dryopteris fragrans* with anti-inflammatory activities. J. Asian Nat. Prod. Res..

[41] Chen X.-Q., Li Y., Cheng X., Wang K., He J., Pan Z.-H., Li M.-M., Peng L.-Y., Xu G., Zhao Q.-S. (2010). Polycyclic Polyprenylated Acylphloroglucinols and Chromone O-Glucosides from *Hypericum henryi* subsp. uraloides. Chem. Biodivers..

[42] An R.B., Jeong G.S., Beom J.-S., Sohn D.H., Kim Y.C. (2009). Chromone glycosides and hepatoprotective constituents of *Hypericum erectum*. Arch. Pharm. Res..

[43] Itoh A., Tanahashi T., Nagakura N., Nishi T. (2003). Two chromone-secoiridoid glycosides and three indole alkaloid glycosides from *Neonauclea sessilifolia*. Phytochemistry.

[44] Abe F., Yamauchi T. (1993). Megastigmanes and flavonoids from the leaves of *Scorodocarpus borneensis*. Phytochemistry.

[45] Gujral V.K., Gupta S.R., Verma K.S. (1979). Structure of undulatoside-B, a new chromone glycoside from *Tecomella undulata*. Indian J. Chem. Sect. B Org. Chem. Incl. Med. Chem..

[46] Yoshimitsu H., Nishida M., Hashimoto F., Tanaka M., Sakata Y., Okawa M., Nohara T. (2007). Chromone and flavonol glycosides from *Delphinium hybridum* cv. “Belladonna Casablanca”. J. Nat. Med..

[47] Shang Z.-P., Meng J.-J., Zhao Q.-C., Su M.-Z., Luo Z., Yang L., Tan J.-J. (2013). Two new chromone glycosides from *Drynaria fortunei*. Fitoterapia.

[48] Tanaka N., Jia Y., Niwa K., Imabayashi K., Tatano Y., Yagi H., Kashiwada Y. (2018). Phloroglucinol derivatives and a chromone glucoside from the leaves of *Myrtus communis*. Tetrahedron.

[49] Yi J., Zhang G., Li B. (2002). Studies on the chemical constituents of *Pseudotsuga sinensis*. Yao Xue Xue Bao.

[50] Tanaka N., Kashiwada Y., Nakano T., Shibata H., Higuchi T., Sekiya M., Ikeshiro Y., Takaishi Y. (2009). Chromone and chromanone glucosides from *Hypericum sikokumontanum* and their anti-Helicobacter pylori activities. Phytochemistry.

[51] Huneck S., Jakupovic J., Follmann G. (1992). The final structures of the lichen chromones galapagin, lobodirin, mollin, and roccellin. Zeitschrift Naturforsch. B.

[52] Li X., Niu L.-T., Meng W.-T., Zhu L.-J., Zhang X., Yao X.-S. (2020). Matteuinterins A-C, three new glycosides from the rhizomes of *Matteuccia intermedia*. J. Asian Nat. Prod. Res..

[53] Hu L., Chen N.-N., Hu Q., Yang C., Yang Q.-S., Wang F.-F. (2014). An Unusual Piceatannol Dimer from *Rheum austral* D. Don with Antioxidant Activity. Molecules.

[54] Zhao H., Wang Z., Cheng J., Li R., Wang Z. (2009). New chromone glucoside from roots of rumex gmelini. Tianran Chanwu Yanjiu Yu Kaifa.

[55] Singh J. (1981). Photochemical investigations on *Cassia multijuga* leaves. Isolation and characterization of new chromone glycosides. Pol. J. Chem..

[56] Singh J. (1982). Two chromone glycosides from *Cassia multijuga*. Phytochemistry.

[57] Kashiwada Y., Nonaka G.I., Nishioka I. (1990). Chromone glucosides from rhubarb. Phytochemistry.

[58] Mou L.-Y., Wei M., Wu H.-Y., Hu L.-J., Li J.-L., Li G.-P. (2020). *O*-beta-d-Glucopyranosyl-2-methylchromone, a new chromone glycoside from the Tibetan medicine plant of *Swertia punicea* Hemsl. Nat. Prod. Res..

[59] Ahmad V., Ullah F., Hussain J., Farooq U., Zubair M., Khan M., Choudhary M. (2004). Tyrosinase inhibitors from Rhododendron collettianum and their structure-activity relationship (SAR) studies. Chem. Pharm. Bull. (Tokyo).

[60] Kim S.B., Ahn J.H., Han S.B., Hwang B.Y., Kim S.Y., Lee M.K. (2012). Anti-adipogenic chromone glycosides from *Cnidium monnieri* fruits in 3T3-L1 cells. Bioorganic Med. Chem. Lett..

[61] Liang H., Zhao Y.Y., Zhang R.Y. (1998). A new chromone glycoside from *Bupleurum chinense*. Chinese Chem. Lett..

[62] Lu T.S., Yi Y.H., Mao S.L., Zhou D.Z., Xu Q.Z., Tang H.F., Zhang S.Y. (2001). A new chromone glycoside from *Cassia siamea* Lam. Chinese Chem. Lett..

[63] Tanaka Y., Honma D., Tamura M., Yanagida A., Zhao P., Shoji T., Tagashira M., Shibusawa Y., Kanda T. (2012). New chromanone and acylphloroglucinol glycosides from the bracts of hops. Phytochem. Lett..

[64] Barash I., Karr A.L., Strobel G.A. (1975). Isolation and characterization of stemphylin, a chromone glucoside from *Stemphylium botrysum*. Plant Physiol..

[65] Do Nascimento B.O., da Silva Neto O.C., Teodoro M.T.F., de Oliveira Silva E., Guedes M.L.S., David J.M. (2020). Macrolobin: A new unusual C-glycoside chromone from *Macrolobium latifolium* and its anticholinesterase and antimicrobial activities. Phytochem. Lett..

[66] Iswaldi I., Arráez-Román D., Rodríguez-Medina I., Beltrán-Debón R., Joven J., Segura-Carretero A., Fernández-Gutiérrez A. (2011). Identification of phenolic compounds in aqueous and ethanolic rooibos extracts (*Aspalathus linearis*) by HPLC-ESI-MS (TOF/IT). Anal. Bioanal. Chem..

[67] Lee H.-H., Shin J.-S., Lee W.-S., Ryu B., Sik Jang D., Lee K.-T. (2016). Biflorin, Isolated from the Flower Buds of *Syzygium aromaticum* L., Suppresses LPS-Induced Inflammatory Mediators via STAT1 Inactivation in Macrophages and Protects Mice from Endotoxin Shock. J. Nat. Product..

[68] Ryu B., Woo J.-H., Kim H.M., Choi J.-H., Jang D.S. (2016). A new acetophenone glycoside from the flower buds of *Syzygium aromaticum* (cloves). Fitoterapia.

[69] Satake T., Kamiya K., Saiki Y., Hama T., Fujimoto Y., Endang H., Umar M. (1998). Chromone C-glycosides from *Baeckea frutescens*. Phytochemistry.

[70] Kamiya K., Satake T. (2010). Chemical constituents of *Baeckea frutescens* leaves inhibit copper-induced low-density lipoprotein oxidation. Fitoterapia.

[71] Ito H., Kasajima N., Tokuda H., Nishino H., Yoshida T. (2004). Dimeric Flavonol Glycoside and Galloylated C-Glucosylchromones from *Kunzea ambigua*. J. Nat. Prod..

[72] Wang S., Feng K., Han L., Fang X., Zhang Y., Yu H., Pang X. (2019). Glycosidic compounds from *Cassia obtusifolia* seeds and their inhibitory effects on OATs, OCTs and OATPs. Phytochem. Lett..

[73] Han A.-R., Paik Y.-S. (2012). Antioxidant and prolyl endopeptidase inhibitory capacities of chromone C-glucosides from the clove buds (*Syzygium aromaticum*). J. Appl. Biol. Chem..

[74] Rehman N.U., Hussain H., Khiat M., Al-Riyami S.A., Csuk R., Khan H.Y., Abbas G., Al-Thani G.S., Green I.R., Al-Harrasi A. (2016). ChemInform Abstract: Aloeverasides A and B: Two Bioactive C-Glucosyl Chromones from Aloe vera Resin. Helv. Chim..

[75] Rehman N.U., Al-Riyami S.A., Hussain H., Ali A., Khan A.L., Al-Harrasi A. (2019). Secondary metabolites from the resins of *Aloe vera* and Commiphora mukul mitigate lipid peroxidation. Acta Pharm..

[76] Kahramanoglu I., Chen C., Chen J., Wan C. (2019). Chemical constituents, antimicrobial activity, and food preservative characteristics of *Aloe vera* gel. Agronomy.

[77] Singh M., Singh J. (1984). Chemical examination of the seeds of *Cassia spectabilis*. Zeitschrift Naturforsch. B.

[78] Agrawal A., Singh J. (1988). Glycosides of two xanthones and a chromone from roots of *Chrozophora prostrata*. Phytochemistry.

[79] Jong-Anurakkun N., Bhandari M.R., Hong G., Kawabata J. (2008). α-Glucosidase inhibitor from Chinese aloes. Fitoterapia.

[80] Lv L., Yang Q.-Y., Zhao Y., Yao C.-S., Sun Y., Yang E.-J., Song K.-S., Mook-Jung I., Fang W.-S. (2008). BACE1 (beta-secretase) inhibitory chromone glycosides from *Aloe vera* and *Aloe nobilis*. Planta Med..

[81] Kim J.H., Yoon J.Y., Yang S.Y., Choi S.K., Kwon S.J., Cho I.S., Jeong M.H., Ho Kim Y., Choi G.S. (2017). Tyrosinase inhibitory components from *Aloe vera* and their antiviral activity. J. Enzyme Inhib. Med. Chem..

[82] Okamura N., Hine N., Harada S., Fujioka T., Mihashi K., Yagi A. (1996). Three chromone components from *Aloe vera* leaves. Phytochemistry.

[83] Wen J., Shi H.-M., Tu P.-F. (2006). Chemical constituents of *Abrus mollis* Hance. Biochem. Syst. Ecol..

[84] Goodger J.Q.D., Seneratne S.L., Nicolle D., Woodrow I.E. (2016). Foliar Essential Oil Glands of Eucalyptus Subgenus Eucalyptus (Myrtaceae) Are a Rich Source of Flavonoids and Related Non-Volatile Constituents. PLoS ONE.

[85] Al-Sayed E., Hamid H.A., Abu El Einin H.M. (2014). Molluscicidal and antischistosomal activities of methanol extracts and isolated compounds from *Eucalyptus globulus* and *Melaleuca styphelioides*. Pharmaceut. Biol..

[86] Zhang Y., Chen Y. (1997). Isobiflorin, a chromone C-glucoside from cloves (*Eugenia caryophyllata*). Phytochemistry.

[87] Gao H.Y., Wang H.Y., Li G.Y., Du X.W., Zhang X.T., Han Y., Huang J., Li X.X., Wang J.H. (2014). Constituents from Zhuyeqing Liquor and their inhibitory effects on nitric oxide production. Phytochem. Lett..

[88] Gao W.-N., Luo J.-G., Kong L.-Y. (2009). Quality evaluation of *Hypericum japonicum* by using high-performance liquid chromatography coupled with photodiode array detector and electrospray ionization tandem mass spectrometry. Biomed. Chromatogr..

[89] Luo G., Zhou M., Ye Q., Mi J., Fang D., Zhang G., Luo Y. (2015). Phenolic derivatives from *Hypericum japonicum*. Nat. Prod. Commun..

[90] Santos S.A.O., Villaverde J.J., Freire C.S.R., Domingues R.M.M., Neto C.P., Silvestre A.J.D. (2012). Phenolic composition and antioxidant activity of *Eucalyptus grandis*, *E. urograndis* (*E. grandis* × *E. urophylla*) and *E. maidenii* bark extracts. Ind. Crops Prod..

[91] Tanaka T., Orii Y., Nonaka G., Nishioka I. (1993). Tannins and related compounds. CXXIII. Chromone, acetophenone and phenylpropanoid glycosides and their galloyl and/or hexahydroxydiphenoyl esters from the leaves of *Syzygium aromaticum* Merr. et Perry. Chem. Pharm. Bull. (Tokyo).

[92] Yuan A., Kang S., Qin L., Ruan B., Fan Y. (1994). Chemical constituents of the leaves of Chinese aloe (*Aloe vera* var. *chinensis*). Zhongcaoyao.

[93] Che Q.-M., Akao T., Hattori M., Kobashi K., Namba T. (1990). Metabolism of aloesin and related cmpounds by human intestinal bacteria: A bacterial cleavage of the C-glucosyl bond and the subsequent reduction of the acetonyl side chain. Chem. Pharm. Bull..

[94] Park M.K., Park J.H., Shin Y.G., Kim W.Y., Lee J.H., Kim K.H. (1996). Neoaloesin A: A new C-glucofuranosyl chromosome from *Aloe barbadensis*. Planta Med..

[95] Lucini L., Pellizzoni M., Pellegrino R., Molinari G.P., Colla G. (2015). Phytochemical constituents and in vitro radical scavenging activity of different Aloe species. Food Chem..

[96] Bisrat D., Dagne E., Van Wyk B.-E., Viljoen A. (2000). Chromones and anthrones from *Aloe marlothii* and *Aloe rupestris*. Phytochemistry.

[97] Conner J.M., Gray A.I., Reynolds T., Waterman P.G. (1990). Anthrone and chromone components of *Aloe cremnophila* and *A. jacksonii* leaf exudates. Phytochemistry.

[98] Wang W., Cuyckens F., Van den Heuvel H., Apers S., Pieters L., Steenkamp V., Stewart M.J., Luyckx V.A., Claeys M. (2003). Structural characterization of chromone C-glucosides in a toxic herbal remedy. Rapid Commun. Mass Spectrom..

[99] Hutter J.A., Salman M., Stavinoha W.B., Satsangi N., Williams R.F., Streeper R.T., Weintraub S.T. (1996). Antiinflammatory C-Glucosyl Chromone from *Aloe barbadensis*. J. Nat. Prod..

[100] Zhong J.S., Huang Y.Y., Zhang T.H., Liu Y.P., Ding W.J., Wu X.F., Xie Z.Y., Luo H.B., Wan J.Z. (2015). Natural phosphodiesterase-4 inhibitors from the leaf skin of *Aloe barbadensis* Miller. Fitoterapia.

[101] Lee S., Do S.G., Kim S.Y., Kim J., Jin Y., Lee C.H. (2012). Mass spectrometry-based metabolite profiling and antioxidant activity of *Aloe vera* (*Aloe barbadensis* Miller) in different growth stages. J. Agric. Food Chem..

[102] Rehman N.U., Hussain H., Khiat M., Khan H.Y., Abbas G., Green I.R., Al-Harrasi A. (2017). Bioactive chemical constituents from the resin of *Aloe vera*. Zeitschrift für Naturforsch. B.

[103] Yagi A., Kanbara T., Morinobut N. (1987). Inhibition of Mushroom-Tyrosinase by Aloe Extract. Planta Med..

[104] Baba K., Kawanishi H., Taniguchi M., Kozawa M. (1993). Chromone glucosides from *Cnidium japonicum*. Phytochemistry.

[105] Kimura Y., Sumiyoshi M., Taniguchi M., Baba K. (2008). Antitumor actions of a chromone glucoside cnidimoside A isolated from *Cnidium japonicum*. J. Nat. Med..

[106] Zhao J., Zhou M., Liu Y., Zhang G., Luo Y. (2011). Chromones and coumarins from the dried fructus of *Cnidium monnieri*. Fitoterapia.

[107] Cisowski W., Grimshaw J. (1988). Glucoside of chromone from *Angelica archangelica* L. and *Archangelica litoralis* Fries. herbs. Pol. J. Chem..

[108] Kopp B., Kubelka E., Reich C., Robien W., Kubelka W. (1991). 4H-Chromenone Glycosides from *Eranthhis hyernalis* (L.) SALISBU. Helv. Chim. Acta.

[109] Kuroda M., Uchida S., Watanabe K., Mimaki Y. (2009). Chromones from the tubers of *Eranthis cilicica* and their antioxidant activity. Phytochemistry.

[110] Bernhardt M., Shaker K.H., Elgamal M.H.A., Seifert K. (2000). The New Bishomoflavone Ononin and Its Glucoside from Ononis vaginalis. Zeitschrift für Naturforsch. C.

[111] Ibrahim S.R.M. (2010). New 2-(2-Phenylethyl)chromone Derivatives from the Seeds of *Cucumis melo* L var. reticulatus. Nat. Prod. Commun..

[112] Quan L., Zhang J., Wu Y. (2017). One Novel 2-(2-Phenylethyl)Chromone Glycoside from *Aquilaria sinensis*. Chem. Nat. Compd..

[113] Liu X., Zhang B., Yang L., Yu G., Wang Z. (2013). Two new chromones and a new flavone glycoside from *Imperata cylindrical*. Zhongguo Tianran Yaowu.

[114] Shao H., Mei W.-L., Kong F.-D., Dong W.-H., Li W., Zhu G.-P., Dai H.-F. (2017). A new 2-(2-phenylethyl)chromone glycoside in Chinese agarwood “Qi-Nan” from *Aquilaria sinensis*. J. Asian Nat. Prod. Res..

[115] Zaher A., Boufellous M., Ouhssine M., Bourkhiss B. (2019). Phytochemical Screening Of An Umbelliferae: *Ammi visnaga* L. (Lam.) In The Region Of Sidi Slimane- North-West Of Morocco. J. Mater. Environ. Sci..

[116] Stevens T.J., Jones B.W., Vidmar T.J., Moran J.J., Manni D.S., Day C.E. (1985). Hypocholesterolemic effect of khellin and khelloside in female cynomolgus monkeys. Arzneimittelforschung.

[117] Günaydin K., Beyazit N. (2004). The chemical investigations on the ripe fruits of *Ammi visnaga* (Lam.) Lamarck growing in Turkey. Nat. Prod. Res..

[118] Liu Y.R., Wu Z.J., Li C.T., Xi F.M., Sun L.N., Chen W.S. (2013). Heracleifolinosides A-F, new triterpene glycosides from *cimicifuga heracleifolia*, and their inhibitory activities against hypoxia and reoxygenation. Planta Med..

[119] Ma S.Y., Shi L.G., Gu Z.B., Wu Y.L., Wei L.B., Wei Q.Q., Gao X.L., Liao N. (2018). Two New Chromone Glycosides from the Roots of *Saposhnikovia divaricata*. Chem. Biodivers..

[120] Jiang Y., Guo F., Chen L., Xu L., Zhang W., Liu B. (2019). The antitumor activity of naturally occurring chromones: A review. Fitoterapia.

[121] Sasaki H., Taguchi H., Endo T., Yosioka I. (1982). The constituents of *Ledebouriella seseloides* Wolff. I. Structures of three new chromones. Chem. Pharm. Bull..

[122] Urbagarova B.M., Shults E.E., Taraskin V.V., Radnaeva L.D., Petrova T.N., Rybalova T.V., Frolova T.S., Pokrovskii A.G., Ganbaatar J. (2020). Chromones and coumarins from *Saposhnikovia divaricata* (Turcz.) Schischk. Growing in Buryatia and Mongolia and their cytotoxicity. J. Ethnopharmacol..

[123] Lemmich J. (1995). Monoterpene, chromone and coumarin glucosides of *Diplolophium buchananii*. Phytochemistry.

[124] Shao Z., Zhang Y., Jiang K., CHEN J., Zuo B. (2003). Chemical constituents from the roots of *Sphallerocarpus gracilis*. Nat. Prod. Res. Dev..

[125] Yan Z., Yang X., Wu J., Su H., Chen C., Chen Y. (2011). Qualitative and quantitative analysis of chemical constituents in traditional Chinese medicinal formula Tong-Xie-Yao-Fang by high-performance liquid chromatography/diode array detection/electrospray ionization tandem mass spectrometry. Anal. Chim. Acta.

[126] Semwal R.B., Semwal D.K., Combrinck S., Viljoen A. (2020). Health benefits of chromones: Common ingredients of our daily diet. Phytochem. Rev..

[127] Khalil N., Bishr M., Desouky S., Salama O. (2020). *Ammi visnaga* L., a potential medicinal plant: A review. Molecules.

[128] An R.-B., Park B.-Y., Kim J.-H., Kwon O.-K., Lee J.-K., Min B.-S., Ahn K.-S., Oh S.-R., Lee H.-K. (2005). Coumarins and chromones from *Angelica genuflexa*. Nat. Prod. Sci..

[129] Baba K., Hata K., Kimura Y., Matsuyama Y., Kozawa M. (1981). Chemical studies of *Angelica japonica* A. Gray. I. On the constituents of the ethyl acetate extract of the root. Chem. Pharm. Bull. (Tokyo).

[130] Shi Q.-Q., Lu J., Peng X.-R., Li D.-S., Zhou L., Qiu M.-H. (2018). Cimitriteromone A–G, Macromolecular Triterpenoid–Chromone Hybrids from the Rhizomes of *Cimicifuga foetida*. J. Org. Chem..

[131] Sun J., Su X., Zhang Z., Hu D., Hou G., Zhao F., Sun J., Cong W., Wang C., Li H. (2021). Separation of three chromones from *Saposhnikovia divaricata* using macroporous resins followed by preparative high-performance liquid chromatography. J. Sep. Sci..

[132] Lu L., Chen J.C., Li Y., Qing C., Wang Y.Y., Nian Y., Qiu M.H. (2012). Studies on the constituents of *Cimicifuga foetida* collected in guizhou province and their cytotoxic activities. Chem. Pharm. Bull..

[133] Wang H., Xu Y., Yuan Z. (2011). Isolation and identification of chemical constituents of roots of *Glehnia littoralis*. J. Shenyang Pharm. Univ..

[134] Zheng M., Jin W., Son K.-H., Chang H.-W., Kim H.-P., Bae K.-H., Kang S.-S. (2005). The constituents isolated from *Peucedanum japonicum* Thunb. and their cyclooxygenase (COX) inhibitory activity. Korean J. Med. Crop Sci..

[135] Akunyili D.N., Akubue P.I. (1986). Schumanniofoside, the antisnake venom principle from the stem bark of *Schumanniophyton magnificum* Harms. J. Ethnopharmacol..

[136] Shi Q.-Q., Gao Y., Lu J., Zhou L., Qiu M.-H. (2020). Two new triterpenoid-chromone hybrids from the rhizomes of *Actaea cimicifuga* L.(syn. *Cimicifuga foetida* L.) and their cytotoxic activities. Nat. Prod. Res..

[137] Brill G.M., Jackson M., Whittern D.N., Buko A.M., Hill P., Chen R.H., Mcalpine J.B. (1994). Altromycins E, F, G, H and I; additional novel components of the altromycin complex. J. Antibiot. (Tokyo).

[138] Kanda N. (1971). A New Antitumor Antibiotic, Kidamycin. I Isolation, Purification and Properties of Kidamycin. J. Antibiot. (Tokyo).

[139] Kondo S., Wakashiro T., Hamada M., Maeda K., Takeuchi T., Umezawa H. (1970). Isolation and characterization of a new antibiotic, neopluramycin. J. Antibiot. (Tokyo).

[140] Kondo S., Miyamoto M., Naganawa H., Takeuchi T., Umezawa H. (1977). Structures of pluramycin A and neopluramycin. J. Antibiot. (Tokyo).

[141] Byrne K.M., Gonda S.K., Hilton B.D. (1985). Largomycin FII chromophore component 4, a new pluramycin antibiotic. J. Antibiot. (Tokyo).

[142] Nadig H., Sèquin U. (1987). Isolation and Structure Elucidation of Some Components of the Antitumor Antibiotic Mixture ‘Rubiflavin'. Helv. Chim. Acta.

[143] Nadig H., Séquin U., Bunge R.H., Hurley T.R., Murphey D.B., French J.C. (1985). Isolation and structure of a new antibiotic related to rubiflavin A. Helv. Chim. Acta.

[144] Schmitz H., Crook K.E., Bush J.A. (1966). Hedamycin, a new antitumor antibiotic. I. Production, isolation, and characterization. Antimicrob. Agents Chemother..

[145] Séquin U., Bedford C.T., Chung S.K., Scott A.I. (1977). The structure of the antibiotic hedamycin. I. Chemical, physical and spectral properties. Helv. Chim. Acta.

[146] Sato Y., Watabe H.-O., Nakazawa T., Shomura T., Yamamoto H., Sezaki M., Kondo S. (1989). Ankinomycin, a potent antitumor antibiotic. J. Antibiot. (Tokyo).

[147] Vertesy L., Barbone F.P., Cashmen E., Decker H., Ehrlich K., Jordan B., Knauf M., Schummer D., Segeth M.P., Wink J. (2001). Pluraflavins, potent antitumor antibiotics from *Saccharothrix* sp. DSM 12931. J. Antibiot. (Tokyo).

[148] Jackson M., Karwowski J.P., Theriault R.J., Hardy D.J., Swanson S.J., Barlow G.J., Tillis P.M., McAlpine J.B. (1990). Altromycins, novel pluramycin-like antibiotics I. Taxonomy of the producing organism, fermentation and antibacterial activity. J. Antibiot. (Tokyo).

[149] Brill G.M., Mcalpine J.B., Whittern D.N., Buko A.M. (1990). Altromycins, novel pluramycin-like antibiotics II. Isolation and elucidation of structure. J. Antibiot. (Tokyo).

[150] Gallivan J.B. (1970). Spectroscopic studies on some chromones. Can. J. Chem..

[151] Griffiths P.J.F., Ellis G.P. (1972). Benzopyrones—VI: The ultraviolet absorption spectra of chromone and 2-substituted chromones. Spectrochim. Acta Part A Mol. Spectrosc..

[152] Staunton J., Barton D., Ollis W.D. (1979). In Comprehensive Organic Chemistry.

[153] Nchinda A. (2001). Chemical Studies of Selected Chromone Derivatives.

[154] Dey P.M. (2012). Methods in Plant Biochemistry.

[155] Markham K.R., Mabry T.J. (1975). Ultraviolet-Visible and Proton Magnetic Resonance Spectroscopy of Flavonoids. The Flavonoids.

[156] Murray R.D.H., McCabe P.H. (1969). Infrared studies of chromones—I: Carbonyl and hydroxyl regions. Tetrahedron.

[157] Liu S., Zhu T., Qiu Y., Qi W., Wu H., Cai B., Lin J. (2019). Chromones and tannins from the fruit of *Euscaphis japonica* var. *wupingensis*. BioResources.

[158] Wu Q.-L., Wang S.-P., Du L.-J., Zhang S.-M., Yang J.-S., Xiao P.-G. (1998). Chromone glycosides and flavonoids from Hypericum japonicum. Phytochemistry.

[159] Speranza G., Dadà G., Lunazzi L., Phytochemistry P.G. (1986). A C-glucosylated 5-methylchromone from *Kenya aloe*. Phytochemistry.

[160] Okamura N., Hine N., Tateyama Y., Nakazawa M., Fujioka T., Mihashi K. (1998). Five chromones from *Aloe vera* leaves. Phytochemistry.

[161] Speranza G., Gramatica P., Dadá G., Manitto P. (1985). Aloeresin C, a bitter C, O-diglucoside from Cape Aloe. Phytochemistry.

[162] Rauwald H.W., Maucher R., Dannhardt G., Kuchta K. (2021). Dihydroisocoumarins, Naphthalenes, and Further Polyketides from Aloe vera and A. plicatilis: Isolation, Identification and Their 5-LOX/COX-1 Inhibiting Potency. Molecules.

[163] Van Heerden F.R., Viljoen A.M., Van Wyk B.E. (2000). 6′-*O*-Coumaroylaloesin from Aloe castanea—A taxonomic marker for Aloe section Anguialoe. Phytochemistry.

[164] Holzapfel C.W., Wessels P.L., Van Wyk B.E., Marais W., Portwig M. (1997). Chromone and aloin derivatives from *Aloe broomii*, *A. Africana* and A. speciosa. Phytochemistry.

[165] Séquin U., Furukawa M. (1978). The structure of the antibiotic hedamycin—III: 13C NMR spectra of hedamycin and kidamycin. Tetrahedron.

